# Intramolecular Vibrational Energy Transfer in the Precatalyst [Mn(ppy)(CO)_4_] Tracked by Dual-Frequency 2D Infrared Spectroscopy

**DOI:** 10.1021/acs.jpca.6c01573

**Published:** 2026-05-18

**Authors:** Stefan Flesch, Barbara Procacci, Sabina Gurung, Shweta Choudhary, Ian J. S. Fairlamb, Jason M. Lynam, Neil T. Hunt

**Affiliations:** Department of Chemistry, 8748 The University of York, Heslington YO10 5DD, York, U.K.

## Abstract

The thermal dissociation of carbon monoxide is a fundamental entry step to many catalytic cycles involving metal carbonyl (MCO) complexes as it reveals a vacant coordination site at the metal center, enabling substrate coordination. Overcoming the dissociation barrier requires sufficient accumulation of energy in vibrational modes with displacement vectors along the reaction coordinate (M-CO distance). Hence, understanding the energy transfer to and from these vibrations is essential in developing a detailed understanding of a precatalyst’s activation pathway. Here, the intramolecular vibrational energy redistribution (IVR) within the heteroleptic metal carbonyl complex [Mn­(ppy)­(CO)_4_] (**1**, ppy = cyclometalated 2-phenylpyridine) in dichloromethane solution has been studied using dual-frequency, two-dimensional infrared spectroscopy. The responses of three vibrational modes localized on the ppy-ligand were monitored following the photoexcitation of carbonyl ligand stretching modes. A rise of signal strength by a factor of 4.7 within the first 35 ps, followed by a decay within ca. 150 ps exemplify the IVR from the metal carbonyl to the organic moiety, and the intermolecular energy transfer (IET) to the solvent, respectively. Moreover, pronounced changes of the spectral shape within the first 10 ps indicate the population of distinct vibrationally excited states. Direct anharmonic coupling between the pumped and probed modes gives rise to the initial spectral features, which include an uncommon, negative anharmonic coupling. These features do not decay single-exponentially as expected, but rise during the first 23 ps instead. This finding is assigned to the population of low-frequency modes (<250 cm^–1^), which are predicted to have a similar coupling pattern toward the measured bands as the CO stretching modes, based on density functional theory (DFT) calculations. The stronger signals predominant at late waiting times have uniform anharmonic shifts of ca. −2.5 cm^–1^ arising from the coupling to medium-frequency modes (250–1200 cm^–1^), which are strongly localized on the ppy-ligand. Due to the distinct signal positions, the time-dependent populations of low- and medium-frequency modes can be evaluated independently. Rate constants for the rise of their populations were found to be 1/52 ps^–1^ and 1/58 ps^–1^, respectively, while the rate constants of depopulation via IET to the solvent are 1/19 ps^–1^ and 1/25 ps^–1^.

## Graphical abstract

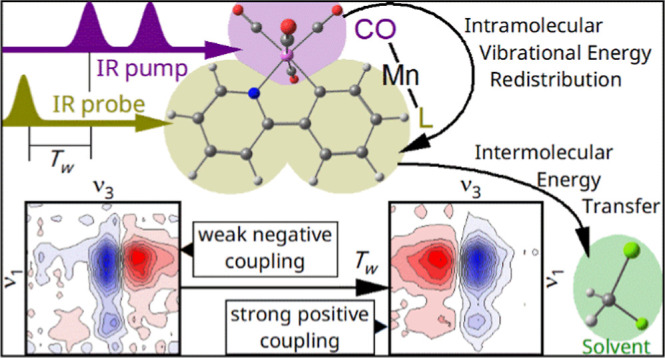

## Introduction

Over the last five decades, transition metal carbonyl complexes have been proven to be readily available and flexible catalysts in a multitude of applications, e.g. carbonylations,^1–4^ hydroformylations^3–5^ or acetic acid synthesis.^3,6^ In particular, complexes of 3d-transition metals have moved into the focus of interest of synthetic chemists due to their higher natural abundance as compared to 4d- and 5d-metals.^3,7^ Under the superordinate aim of extending the capability and improving the efficiency of these catalytic species, the underlying reaction mechanisms have been subjected to tremendous research efforts into understanding (thermal) precatalyst activation, steady-state catalysis and catalyst deactivation.^8–13^ In this context, time-resolved infrared (TRIR) spectroscopy has been employed widely as a tool enabling insight into relevant reaction steps and intermediates occurring on time-scales spanning femtoseconds to hours.^13–25^ Metal carbonyl (MCO) complexes in particular represent ideal targets for infrared (IR) spectroscopy due to the high absorption cross-section of carbonyl stretching modes, their resonance frequencies being located in a spectral window mostly free of absorption bands of common solvents and their sensitivity to the properties of the attached metal center and the residual ligand sphere.^11^

Prominent and well-investigated examples are the low-valent manganese catalysts [Mn_2_(CO)_10_], **2**, and [MnBr­(CO)_5_], **3**, which can be applied in borylations,^26^ hydroborations,^27^ hydrosilylations,^28–31^ and a variety of radical reactions initiated by halide-abstraction from organic substrates.^32–37^ Arguably, the most intriguing reactions are C–H bond activation/C–C bond formation sequences catalyzed by **3**,^38–41^ e.g. the coupling of 2-phenylpyridine (ppyH) and phenyl acetylene^42^ (see Scheme 1). Mechanistic studies^15,43–48^ revealed this reaction to proceed via the chelated complex [Mn­(ppy)­(CO)_4_] (ppy = cyclometalated 2-phenylpyridine), **1**, which by itself is a stable compound and well-suited catalyst precursor for C–C coupling reactions. Like many MCO complexes, **1** and **3** require either photochemical or thermal activation to release a carbonyl ligand and provide a vacant binding site for coordination of a substrate. Hence, the cleavage of metal–carbonyl bonds represents an important and thoroughly investigated, albeit still hardly understood, reaction step.

**Scheme 1:**
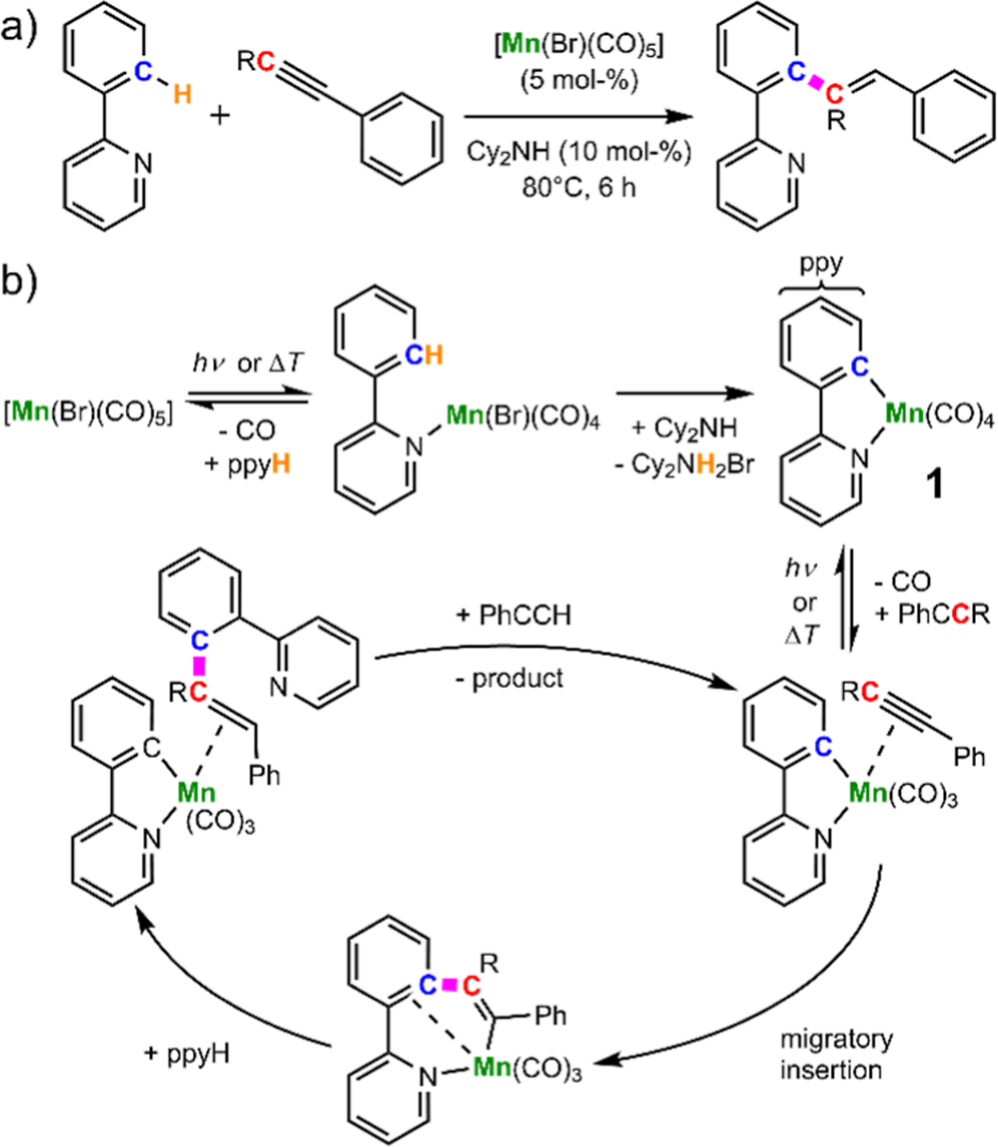
(a) Example C–H Bond Functionalization Reaction Catalyzed by [Mn­(Br)­(CO)_5_] and (b) Key Steps of the Underlying Catalytic Cycle Discovered by TRIR Measurements, Including the Intermediate [Mn­(ppy)­(CO)_4_][Bibr ref15]

While photochemical dissociation occurs on the potential energy surface of an excited electronic state that is repulsive along the M–CO bond axis,^49,50^ thermal dissociation takes place on the electronic ground state surface via the population of highly excited vibrational states.^51,52^ Thus, details of the energy dissipation within a MCO complex directly influence its thermal CO release and consequently its capabilities as a precatalyst. For this reason, we anticipate that conclusions about the catalytic activity of a MCO complex can be derived from knowledge of its vibrational structure and dynamics. Such a (vibrational) structure–property correlation is, however, hitherto unknown, and will require detailed, quantitative studies of a variety of model systems.

Two-dimensional infrared (2DIR) and IR-pump/IR-probe spectroscopy are optimal tools in this regard, as they allow direct experimental access to the coupling of vibrational modes and their relaxation dynamics. Recently, such experiments were conducted on the manganese-based (pre)­catalysts **1** ^53^ and **2** ^54^ in various solvents and revealed a fundamental difference in the relaxation of their MCO modes. In both complexes, the spectro-temporal evolution is well described double-exponentially, whereby the faster component, which was assigned to the intramolecular vibrational energy redistribution (IVR) between the carbonyl modes, varies between the different 2DIR signals but proceeds in general within a few picoseconds. However, the time constant of the second component, which is the intermolecular energy transfer (IET) to the solvent bath, amounts to only 30–60 ps in the case of complex **1**,^53^ but to 80–200 ps in the case of complex **2**,^54^ depending on the solvent. Considering that the same functional group is excited in both cases, a difference in vibrational lifetime of a factor of ca. three seems surprising and prompted us to search for the underlying reason. Obviously, there are three major differences between the two complexes: while **1** is a heteroleptic and mononuclear Mn­(I) complex, **2** is a homoleptic and dinuclear Mn(0) complex. Literature reports of studies on various other metal carbonyl complexes suggest the presence/absence of an organic ligand (note: different to CO), rather than that of additional metal centers or the oxidation state, to be responsible for the drastic change in vibrational lifetime. In general, metal carbonyl complexes bearing at least one organic ligand^55–58^ relax much faster than their (quasi-)­homoleptic relatives,^55,59–61^ regardless of the oxidation state or the number of the metal atoms. Despite the large number of examples underlining this correlation between structure and vibrational lifetime, a more detailed explanation for this tendency is lacking. Clearly, organic ligands (L) in heteroleptic L-MCO complexes like **1** seem to take part in the relaxation mechanism of the carbonyl stretching modes. Hence, we hypothesize that these complexes first undergo a vibrational energy transfer to the organic backbone, which then transfers the energy into the solvent, and that this pathway is faster than the direct IET from MCO groups. In contrast, the latter is supposedly the predominant path in (quasi-) homoleptic complexes.

Vibrational energy transfer in organic^62–66^ and metal–organic^56,67–71^ molecules has been investigated previously by means of dual-frequency, relaxation-assisted 2DIR spectroscopy (RA 2DIR).^72,73^ This method employs excitation (pump) and measuring (probe) pulses of different center frequencies, in contrast to conventional, single-frequency 2DIR spectroscopy. This way, the response of a vibrational reporter mode localized on a functional group of the molecule of interest can be tracked after excitation of a mode localized at a different, spatially separated group. In such an experiment, two types of signals are typically observed: First, cross-peaks arising from direct anharmonic coupling of the pumped and probed modes. Since the spatial and frequency separation results in a weak coupling of the two modes, these signals are usually small in intensity. Second, cross-peaks, which result from energy transfer from the pumped mode into low-frequency modes with stronger coupling to the reporter mode. This increased coupling leads to a rise of the cross-peak intensity up to the energy transport time, *T*_w,max_, after which the peaks decline again due to IET to the solvent. The ratio of initial and maximal cross-peak intensity is referred to as amplification factor, γ.^73^

In this study, we conducted dual-frequency 2DIR and IR-pump/IR-probe measurements of **1** in dichloromethane (CH_2_Cl_2_) solution to verify our hypothesis. To this end, we excited the complex using pump pulses resonant with its MCO stretching modes (1920–2010 cm^–1^) and probed the response of vibrational modes centered on the ppy-moiety (1450–1620 cm^–1^). Combining a kinetic evaluation and anharmonic couplings extracted from these 2DIR and pump/probe data with results from complementary single-frequency experiments in both spectral regions and anharmonic frequency calculations based on density functional theory (DFT), we derive a novel model of the vibrational relaxation mechanism of **1**.

## Methods

### Experimental Methods

Compound **1** was prepared as reported by reaction of 2-phenylpyridine with benzylmanganese­(I) pentacarbonyl.^74^ The synthesis of **1** is summarized in the Supporting Information, SI., Section 1. For the measurements, a solution of **1** in dichloromethane (CH_2_Cl_2_) under air, having a concentration of ca. 3.3 mM, was placed into a transmission cell (Harrick) equipped with two CaF_2_ windows and an optical path length of 100 μm set by a PTFE spacer. The stationary FTIR absorption spectrum was recorded on a Vertex 80 spectrometer (Bruker) with a N_2_-purged sample compartment, a spectral resolution of 1 cm^–1^, averaged over 128 scans and referenced against the neat solvent. Single- and dual-frequency 2DIR spectroscopy employed an ultrafast spectrometer as described elsewhere.^53^ Details are also provided in the Supporting Information document. The center frequencies of the pump and probe pulse were set to either 1550 cm^–1^ or 2000 cm^–1^ depending on the considered spectral region. 2DIR spectra were recorded under a (ZZYY) polarization scheme (here, each letter represents the relative polarization of the two pump pulses, the probe pulse and the emitted field, respectively, in this order. “*Y*” is perpendicular to “*Z*”.) Pump/probe experiments were performed under both (ZZYY) and (ZZZZ) polarization. The spectra shown represent magic angle conditions calculated from measurements under both polarizations.

### Computational Methods

Quantum chemical calculations were conducted using the program Gaussian 16.^75^ The geometry of complex **1** was optimized and the harmonic and anharmonic vibrational frequencies and coupling constants were calculated for the optimized structures. The absence of negative eigenvalues of the Hessian matrix confirmed these structures to be true minima of the potential energy surface. Both geometry optimization and frequency calculation were conducted with every combination of the density functionals BP86,^76,77^ PBE^78,79^ and BLYP^76,80,81^ and the basis sets def2-SVP^82–84^ and def2-TZVP,^83–85^ with and without empirical dispersion correction D3 ^86^ with Becke–Johnson damping (BJ),^87^ and with and without the implicit solvation model CPCM^88,89^ to model CH_2_Cl_2_ solution. While the exact results of the calculations depend on the computational model employed, general trends can be extracted (see Supporting Information, Section 6.1 for details). We believe these tendencies to be more reliable than the output of a single calculation.

In more detail, density fitting^90,91^ using the auxiliary basis set W06 ^83,84^ was employed. The numerical integration grid was set to “superfine” in order to facilitate a more accurate description of low-frequency modes.^92^ The density of integration points in the CPCM was set to 25.0. All other settings equaled the program’s default values. Based on previous optimization investigations,^93^ the results shown in the following sections refer to the calculation with BP86/def2-SVP without CPCM and without D3BJ. Moreover, this computational model in particular emphasizes the trends identified by comparison of the various models.

## Results and Discussion

### Stationary FTIR Spectrum

The stationary absorption spectrum of **1** in CH_2_Cl_2_ solution in the mid-infrared (mIR) region is shown in Figure 1. The spectrum can be subdivided into two parts, which span from 1900 cm^–1^ to 2100 cm^–1^ and from 1450 cm^–1^ to 1620 cm^–1^. The former region comprises four distinct, broad absorption bands at 2075, 1990, 1976, and 1932 cm^–1^ with extinction coefficients from 1200 M^–1^cm^–1^ to 4000 M^–1^cm^–1^ and full-peak width at half-maximum (fwhm) values of 4.5 cm^–1^ (band at 2075 cm^–1^) or 15–17 cm^–1^ (other bands). These bands originate from the combinations of the four CO stretching vibrations of complex **1**, which is discussed in detail in ref 53 For simplicity, these bands will be referred to throughout this work as **CO1–4** in decreasing order of frequency.

**Figure 1:**
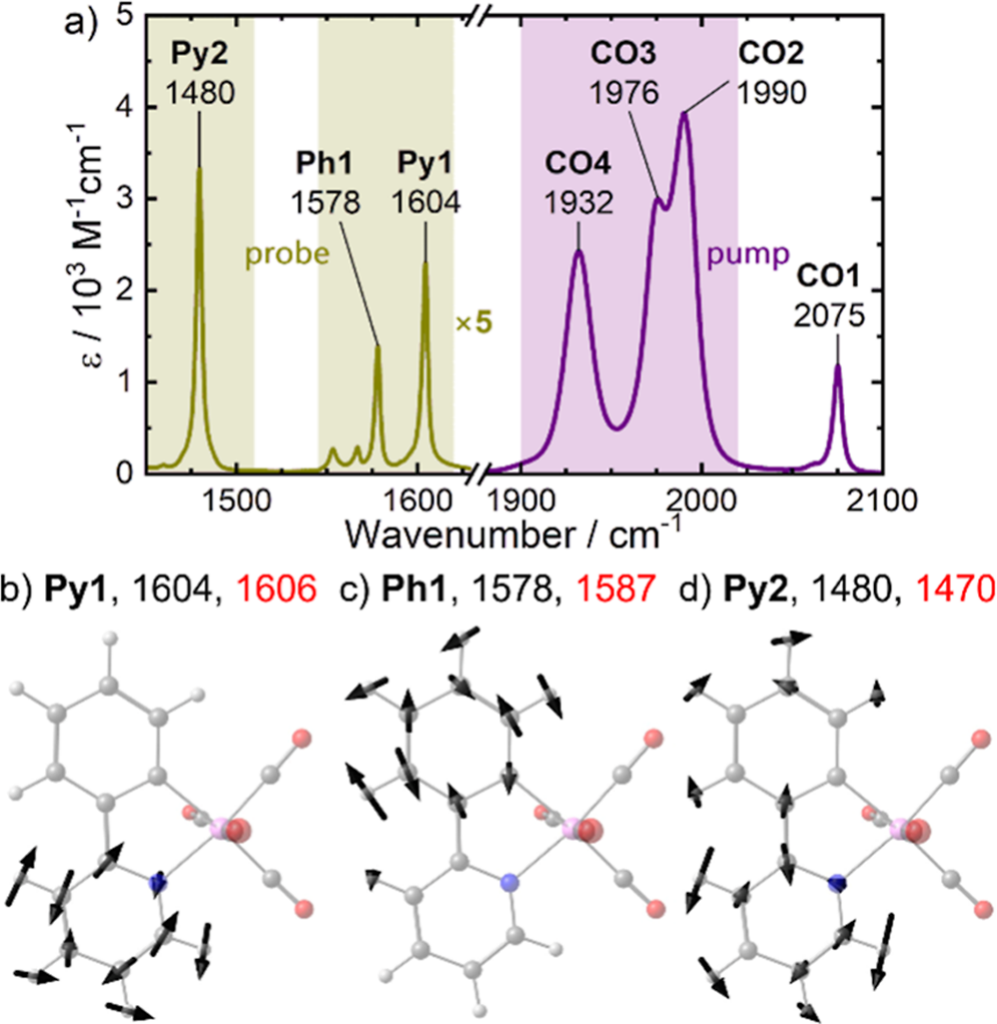
(a) Stationary FTIR absorption spectrum of **1** in room-temperature CH_2_Cl_2_ solution. The bands shown in violet correspond to the CO stretching vibrations of the complex, those shown in yellow to ppy-centered vibrations. Note that the extinction coefficients of the latter are scaled up by a factor of 5. The violet area emphasizes the considered pump (excitation) range, the yellow areas the probe (measurement) ranges. (b)—(d) Ball-and-stick model of the DFT-optimized structure of **1** (H—white, C—gray, N—blue, O—red, Mn—pink) with displacement vectors (black) of the three modes probed in this study. Black and red numbers indicate experimental and calculated resonance frequencies in cm^–1^, respectively, and bold characters represent a shorthand notation used throughout this work.

The second region is dominated by three sharp (fwhm = 3.5 cm^–1^) bands peaking at 1604, 1578, and 1480 cm^–1^, with extinction coefficients of 460, 280, and 670 M^–1^cm^–1^, respectively. According to frequency calculations based on DFT, these bands correspond to aromatic C=C stretching vibrations at the pyridinyl and phenyl moiety, and an in-plane C–H bending vibration at the pyridinyl ring, respectively (c.f. Figure 1b–d). These are equivalent to the ^o^ *D*_6_ and ^o^ *D*_7_ modes in the systematic Mulliken/Herzberg labeling scheme^94–96^ for ortho-disubstituted benzene rings described by Tuttle and Wright.^97^ Due to their localization on the ppy-ligand and the fact that there are no other absorption bands of comparable strength in the mIR region, these three bands are the most promising reporters to track the IVR to this ligand. Here, they will be referred to as **Py1**, **Ph1** and **Py2** to emphasize their localization on the respective functional groups.

### Single-Frequency CO-Pump/CO-Probe Spectroscopy

The vibrational relaxation of the CO stretching modes of **1** in various solvents has been reported previously.^53^ The time evolution of the transient signals was well-described by double-exponential fitting functions, where the first process was assigned to IVR between the MCO modes. The rate constants were found to fall in the range <1/10 ps^–1^ depending on the transition (2DIR peak) considered. The second process manifested itself in a collective decay of all transient MCO signals with rate constants between 1/30 ps^–1^ and 1/60 ps^–1^, depending on the solvent, and, thus, was assigned to the depopulation of the MCO modes.

Here, we performed CO-pump/CO-probe experiments on **1** using otherwise identical settings to those of the dual-frequency experiments described in the following sections, i.e. in the same solvent, CH_2_Cl_2_, and using identical pump pulses, to ensure quantitative comparability. Pump/probe spectra are equivalent to a projection of 2DIR spectra onto the probe wavenumber axis. Such experiments allow the efficient collection of data with a higher point density in the waiting time domain at the cost of losing the resolution along the pump wavenumber axis. Transient spectra and kinetic traces obtained this way are shown in Figure 2. Fully consistent with the findings from the previous work,^53^ the kinetic traces needed, in general, to be fitted to double-exponential functions (eq 3). The second, common rate constant that describes the relaxation of the collective CO stretching modes (**COn**, *n* = 1,2,3,4) was found to be equivalent to 1/(58.8 ± 0.2 ps).

**Figure 2:**
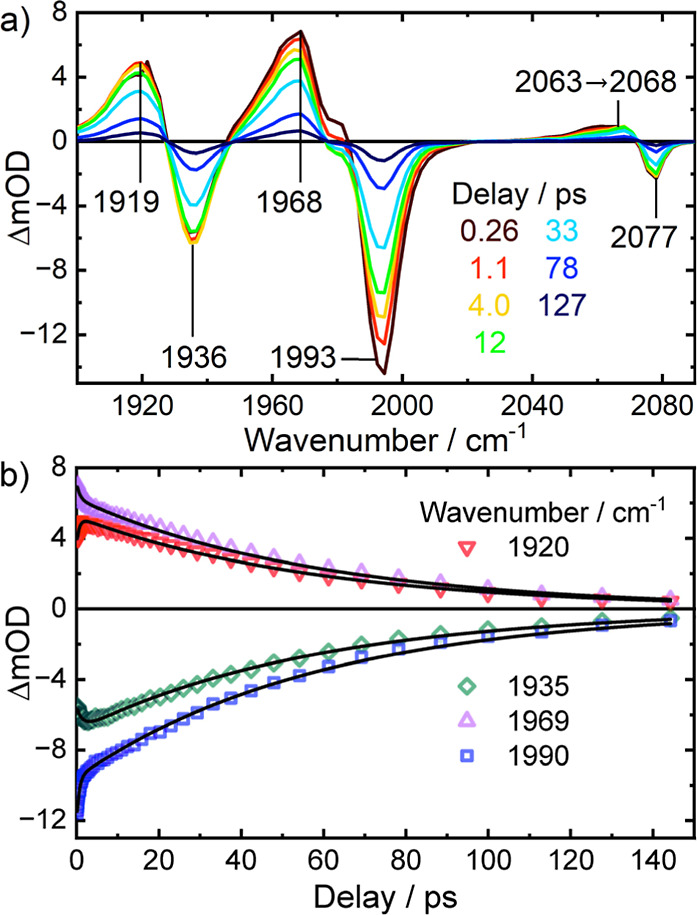
CO-pump/CO-probe (a) transient spectra and (b) kinetic traces of **1** in CH_2_Cl_2_ solution, recorded at selected delays/wavenumbers as given in the legends. Both pump and probe pulses were centered at 2000 cm^–1^. Black numbers in (a) indicate peak positions in cm^–1^. Symbols in (b) are experimental data and black lines show the best fits to double-exponential functions with a global decay rate.

### Dual-Frequency 2D Infrared Spectroscopy

Example 2D CO-stretch pump/ppy-mode probe infrared spectra recorded at two selected waiting times, *T*_w_ = 0.25 and 30 ps, are shown in Figure 3a,b. Selected probe spectra (slices through the 2DIR spectra with constant pump wavenumber, ${\mathrm{\nu ̃}}_{1}$ ) are displayed in Figure 3c and the time evolution of the 2DIR signal strength is shown in Figure 3d. (All 2DIR spectra are given in the Supporting Information, Figure S1.). As Figure 3a demonstrates, there are small, but nonzero ground state bleaching (GSB) signals of the **Py1**, **Ph1** and **Py2** modes present even at the earliest accessible waiting times. Indeed, these signals build up during the duration of the pump pulse itself. They appear at pump wavenumbers equivalent to those of the stationary absorption bands of the **CO2–4** modes of the complex, indicating their origin to be the excitation of those modes. Positive features, also called transient absorptions (TA), are observed at the early waiting times, too, albeit these are less pronounced. Interestingly, the spectral position of the early transient absorptions depends on the excitation wavenumber, as summarized in Table 1. Pumping the **CO3** and **CO2** modes (1960–2010 cm^–1^) gives rise to an up-shifted TA relative to the GSB of the **Py2** mode. By fitting the probe spectra, we obtain a value of +2.1 cm^–1^ for the anharmonic frequency shift. The TA of the **Py1** mode spectrally almost coincides with the GSB but it is broader such that it extends to both positive and negative frequencies relative to the GSB. In contrast, the (fitted) center frequencies of these two modes are ca. 0 (**Py2**) and −0.8 cm^–1^ (**Py1**) shifted with respect to the GSB signals when exciting the **CO4** mode (1920–1940 cm^–1^). The TA of **Ph1** is down-shifted by ca. −2.5 cm^–1^ for all pump frequencies. At later waiting times, the signal strengths of the GSB and TA of all three signals increase in a similar manner over the course of the first 30–40 ps after excitation, and subsequently decline again within the next ca. 150 ps (Figure 3d). The change of signal strengths is accompanied by a change in spectral shape. While the positively shifted TA of **Py2** after pumping **CO2/3** seemingly disappears within the first 10 ps, a new TA, downshifted compared to the GSB signal, rises.

**Figure 3:**
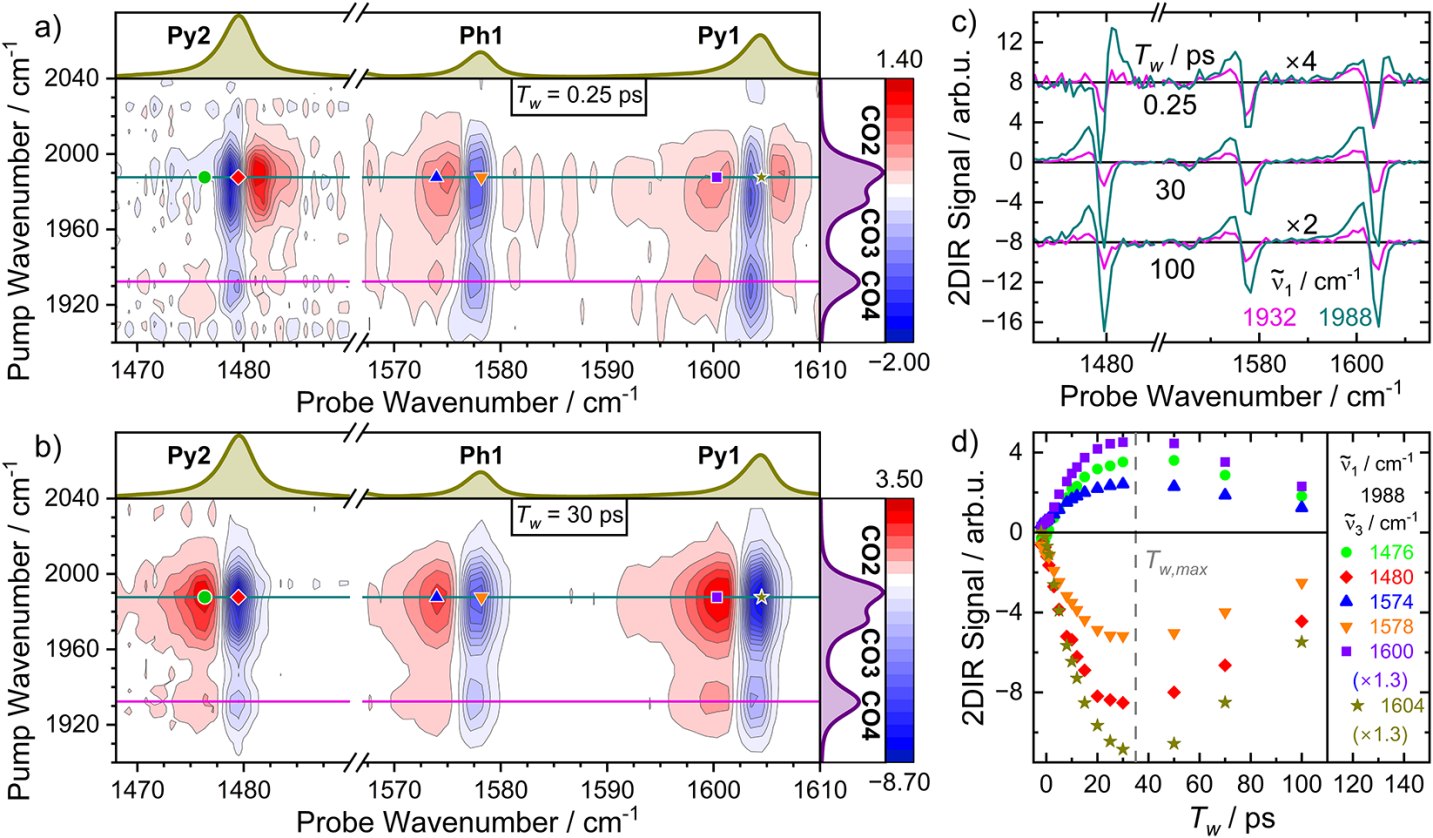
(a) and (b) Dual-frequency 2DIR spectra of **1** in CH_2_Cl_2_ recorded at waiting times of 0.25 and 30 ps, respectively. The violet and yellow spectra attached to the right and upper borders show the stationary spectrum of **1** in the displayed pump and probe region. (c) Slices though the 2DIR spectra at waiting times and pump wavenumbers as given in the legends, and marked in (a) and (b) as colored lines. (d) Dependence of the 2DIR signal strength on the waiting time at a pump wavenumber of 1988 cm^–1^ and probe wavenumbers as given in the legend, and marked in (a) and (b) as colored symbols. The gray, vertical line marks the energy transport time.

**Table 1:** Frequency Shifts of the Considered Modes, Extracted from the 2DIR Spectra at Selected Waiting Times and Excitation Frequencies by Fitting the Transient Signals Using Two Lorentzian Functions (unless Mentioned Otherwise)

*T*_w_/ps	${\mathrm{\nu ̃}}_{1}$ /cm^–1^	predominant component	Δ*ν̃*(Py2)/cm^–1^	Δ*ν̃*(Ph1)/cm^–1^	Δ*ν̃*(Py1)/cm^–1^
0.25	1970–2000	*S*_A_	+2.1 ± 0.3	–2.1 ± 0.5	–0.2 ± 0.2^b^
0.25	1928–1936	*S*_A_ & *S*_B_	≈0	–2.5 ± 0.3^a^	–0.8 ± 0.2^b^
30	all	*S*_B_	–2.4 ± 0.4	–3.1 ± 0.3	–2.6 ± 0.4
100	all	*S*_B_	–2.7 ± 0.6	–2.8 ± 0.2	–2.5 ± 0.5

a Using two Gaussian functions.

b Using two pseudo-Voigt functions.

At late waiting times (Figure 3b), the TAs of all three modes have a similar total signal strength to their corresponding bleaching signal and appear −2.4 – −3.1 cm^–1^ lower in wavenumber (c.f. Table 1). Moreover, the shape of the probe spectra is now independent of the excitation frequency, i.e. all probe spectra at late waiting times (>ca. 10 ps) share the same shape, but are scaled by a factor dependent on *T*_w_ and ${\mathrm{\nu ̃}}_{1}$ .

The spectral changes proceeding during the first few picoseconds imply the presence of two distinct spectral components, which originate from two distinct states of complex **1**. We will call the observed transient spectra *S*_A_ and *S*_B_ in the following, where *S*_A_ is present at early *T*_w_ and characterized by the coupling pattern with a positive spectral shift of the **Py2** band and the broad TA of the **Py1** mode insignificantly shifted relative to its GSB signal. In contrast, *S*_B_ is predominant at later waiting times and characterized by negative anharmonic frequency shifts of −2.4 – −3.1 cm^–1^ of all three signals. Although the spectra directly after **CO4** (1920–1940 cm^–1^) excitation are notably distinct from those at higher excitation frequencies, i.e. **CO2/3** excitation, which could be interpreted as a third component, we assume this to be due to of a superposition of the spectra *S*_A_ and *S*_B_. This interpretation is supported by a singular value decomposition (SVD) of all probe spectra (of all *T*_w_ and ${\mathrm{\nu ̃}}_{1}$ ), which yielded only two singular vectors that could be differentiated from experimental noise and artifacts (see Figure S2).

It should be mentioned that all anharmonic frequency shifts, Δν̃, obtained here by fitting of the probe spectra are smaller than the band widths of the underlying transitions (fwhm of ca. 3.5 cm^–1^ in all three cases). It is well-known that superpositions of TAs and GSB signals with a separation of less than the bandwidth of the transition lead, first, to a large mutual cancelation of the positive and negative signal contribution.^98,99^ For such small peak separations, the signal amplitude is approximately proportional to Δν̃.^100^ Second, the apparent separation, i.e. the spacing between the maximum of the TA and the minimum of the GSB, is similar to the bandwidth. Although peak fitting allows, in theory, an extraction of the real peak separation from strongly overlapping signals, this approach is prone to experimental noise. While the signal-to-noise ratio at late waiting times is sufficient to determine anharmonic frequency shifts as precisely as the resolution of the experiment (ca. 1 cm^–1^), the low signal strength at early waiting times hamper an accurate determination. Thus, the values given in Table 1 for *T*_w_ = 0.25 ps merely reveal the sign of the anharmonic frequency shifts and that their absolute values are smaller than the 3.5 cm^–1^, the bandwidth of the **Py1/2** and **Ph1** bands.

### Kinetic Evaluation

The energy transport time and amplification factor of the considered cross-peaks can be extracted from fits of the 2DIR kinetic traces using double-exponential functions. They amount to *T*_w,max_ = (35.0 ± 0.7) ps and γ = 4.7 ± 0.7, respectively. While similar transport times were previously reported for ligand-to-ligand energy transfers in other metal complexes, the amplification factor found here is comparably small.^67,100^ This can be attributed to the short distance between the pumped and probed functional group, resulting in relatively strong cross-peaks due to direct coupling.

In order to gain more detailed insight into the kinetics of the system, we attempted to explore the time-dependence of spectra *S*_A_ and *S*_B_ independently of each other. Since the signals of both spectra strongly overlap, we applied the following procedure of deconvolution: First, $S_{A}\left({\mathrm{\nu ̃}}_{3}\right)$ , was estimated by averaging the probe spectra in the pump frequency range 1975 cm^–1^ ≤ ${\mathrm{\nu ̃}}_{1}$ ≤ 2000 cm^–1^ and at *T*_w_ = 0.25 ps, since the contribution of *S*_B_ to those spectra appears to be negligible. To reduce the effect of experimental noise, *S*_A_ was fitted as a superposition of six Lorentzian functions (Table S1 and Figure S3). As discussed above, a SVD of the full data set indicates a composition of only these two spectral components. Thus, we can set up a system of linear equations (eq 1), which described each point of the 2DIR data set $S\left({\mathrm{\nu ̃}}_{1},{\mathrm{\nu ̃}}_{3},T_{w}\right)$ as a weighed sum of the signal strengths of *S*_A_ and *S*_B_ at this probe frequency, ${\mathrm{\nu ̃}}_{3}$

1 $$S\left({\mathrm{\nu ̃}}_{1},{\mathrm{\nu ̃}}_{3},T_{w}\right)=S_{A}\left({\mathrm{\nu ̃}}_{3}\right)c_{A}\left({\mathrm{\nu ̃}}_{1},T_{w}\right)+{S_{B}\left({\mathrm{\nu ̃}}_{3}\right)c}_{B}\left({\mathrm{\nu ̃}}_{1},T_{w}\right)$$

with the coefficients $c_{A}\left({\mathrm{\nu ̃}}_{1},T_{w}\right)$ and $c_{B}\left({\mathrm{\nu ̃}}_{1},T_{w}\right)$ being the time- and pump frequency-dependent weighting factors of *S*_A_ and *S*_B_, respectively. These coefficients are proportional to the population of the states of **1** that give rise to the spectra *S*_A_ and *S*_B_ and, thus, contain information about the kinetics of the system. Using eq 1, *S*_B_, *c*_A_ and *c*_B_ were calculated in an iterative, numerical least-squares-deviation optimization with the fixed, fitted *S*_A_. In detail, the deviation of the model w.r.t. the experimental data was determined at the beginning of each iteration step. Subsequently, each entry of *S*_B_, *c*_A_ and *c*_B_ was displaced in both directions, the accuracy of the model with the new parameter evaluated and a numerical gradient calculated. Then, all parameters were changed according to this gradient to decrease the deviation from the experimental data in the next iteration step. In doing so, both *S*_A_ and *S*_B_ were kept square-normalized. Subsequently, *S*_B_ was fitted using Lorentzian functions as well. The raw and fitted spectra and their time-dependent coefficients at selected excitation frequencies obtained this way are displayed in Figure 4. In addition to the 2DIR measurements, we recorded complementary dual-frequency CO-pump/ppy-probe experiments (see Figure S4) and applied the same procedure to those. The time-dependent coefficients obtained from these measurements are also shown in Figure 4b,c.

**Figure 4:**
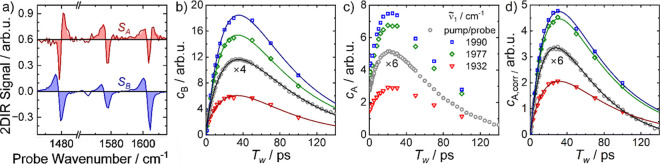
(a) Estimated, square-normalized spectra *S*_A_ (red) and *S*_B_ (blue) obtained using the fitting procedure described in the main text. Solid lines are raw spectra, filled areas show fits using six Lorentzian functions. (b) and (c) Time-dependent coefficients *c*_B_ and *c*_A_ of the spectra *S*_B_ and *S*_A_, respectively, describing their contribution to the 2DIR spectra at pump frequencies or to pump/probe spectra as stated in the legend of panel (c). (d) Coefficients *c*_A,corr_ after subtraction of an exponential decay function with *k* = 1/58.8 ps^–1^ from *c*_A_. Symbols in (b)-(d) represent data points derived from eq 1 and lines double-exponential fit functions.

The time-dependence of *c*_B_ is consistent with the assignment of the spectral features *S*_B_ to energy transfer peaks. The coefficient starts with a value of ca. 0 at *T*_w_ = 0 and increases up to the aforementioned energy transport time, before decaying to the baseline. The evolution can be described well by a double-exponential function with averaged rise and decay rates of 1/59 ps^–1^ and 1/25 ps^–1^, respectively. Example rate constants are given in Table 2 (see Table S2 for other pump frequencies). It is well-known that energy transfer in metal complexes is often limited by the long lifetime of the vibrationally excited state of the donor ligand.^56,62,67^ Here, this quality manifests itself in the rate constant of the rise process being equivalent to the lifetime of the collective, excited **COn** modes of 1/58.8 ps^–1^. Previous studies have employed a convolution of an exponential decay function (eq 2) and a double-exponential rise-and-decay function (eq 3) in order to extract the energy transfer rate independently of the decline in population of the donor state, see eq 4. In this approach, the single-exponential function has a rate constant, *k*_1_, equivalent to the relaxation rate of the **COn** modes and was fixed to 1/58.8 ps^–1^ here. The rate constants of the double-exponential function, *k*_2_ and *k*_3_, describe the rise and decay of the energy transfer peaks, respectively, in the fictive case that the lifetime of the **COn** modes was infinitely short.

2 $$m\left(T_{w}\right)=A_{1}\cdot \exp\left(-k_{1}\cdot T_{w}\right)$$

3 $$d\left(T_{w}\right)=A_{2}\cdot \exp\left(-k_{2}{\cdot T}_{w}\right)+A_{3}\cdot \exp\left(-k_{3}\cdot T_{w}\right)$$

4 $$c\left(T_{w}\right)=\left(d{\left(T_{w}\right)}^{*}m\left(T_{w}\right)\right)=$$

$$\frac{A_{1}A_{2}}{k_{2}-k_{1}}\cdot \left(\exp\left(-k_{1}{\cdot T}_{w}\right)-\exp\left(-k_{2}{\cdot T}_{w}\right)\right)+\frac{A_{1}A_{3}}{k_{3}-k_{1}}\cdot \left(\exp\left(-k_{1}{\cdot T}_{w}\right)-\exp\left(-k_{3}{\cdot T}_{w}\right)\right)$$

**Table 2:** Rate Constants of Double-Exponential Rise-Decay Functions Optimized to Describe the Time-dependent Coefficients, *c*_A,corr_ and *c*_B_, in Dependence on the Pump Frequency

*ν̃*_1_/cm^-1^ mode	1/*k*_1_ (*c*_A,corr_)/ps	1/*k*_2_ (*c*_A,corr_)/ps	1/*k*_1_ (*c*_B_)/ps	1/*k*_2_ (*c*_B_)/ps
1990, **CO2**	52 ± 5	19 ± 2	58 ± 3	23 ± 1
1977, **CO3**	51 ± 5	20 ± 2	62 ± 4	21 ± 1
1932, **CO4**	54 ± 12	20 ± 4	50 ± 12	27 ± 7
pump/probe	46 ± 3	19 ± 1	56 ± 1	25 ± 1

Applying this fit function on the coefficient *c*_B_ returns values of *k*_2_ larger (for many pump frequencies significantly larger) than 1/0.1 ps^–1^, which exceeds the time resolution of the experiment. Thus, we conclude that the modes that couple to **Py1/2** and **Ph1** and which are responsible for spectrum *S*_B_ are instantaneously populated upon depopulation of the **COn** modes, i.e. without any intermediate state. This behavior was expected, since **Py1/2** and **Ph1** are delocalized over the pyridinyl and phenyl ring, respectively, and the spatial distance between both rings and the CO ligands is small, so that a direct energy transfer is likely. Moreover, energy transfers within the ppy ligand itself are, similarly to the IVRs between the **COn** modes,^53^ likely much faster than energy transfer to the ppy ligand. Thus, the excess energy will be distributed quickly between the ppy-centered modes, so that only this internally thermalized state can be observed, but no other intermediate states.

*c*_A_, on the other hand, shows an unexpected behavior. The coefficient has a nonzero value at *T*_w_ = 0, representing that spectrum *S*_A_ builds up within the duration of the pump pulse. This implies that *S*_A_ originates from the direct coupling of the **Py1/2** and **Ph1** modes to the excited MCO modes. However, if that was the only contributing factor, spectrum *S*_A_ would be expected to decay strictly following a single-exponential function with the same rate constant as was obtained from the CO-pump/CO-probe experiments. Instead, our fitting procedure yields an initial rise of spectrum *S*_A_ up to a maximum at *T*_w_ ≈ 23 ps. We verified the validity of this finding by repeating the fitting procedure while enforcing a single- or double-exponential evolution of *c*_A_ (and a double-exponential one for *c*_B_), as shown in the Supporting Information, Section 4.4. Indeed, we found a systematic inaccuracy in the models with single-exponential *c*_A_ in the probe range around 1481 cm^–1^, i.e. where *S*_A_ is most prominent. Thus, we conclude that the initial rise of signals strength of *S*_A_ is a real effect. This indicates that at least one other state, in addition to **COn**, contributes to *S*_A_ (see Supporting Information, Section 4.3 for a more detailed discussion).

In order to discover the kinetics of this/these other state(s), the contribution of **COn** to *c*_A_, a single-exponential function with *k* = 1/58.8 ps^–1^, needs to be subtracted. The resulting “corrected” coefficients, *c*_A,corr_, are displayed in Figure 4d. They peak at *T*_w_ ≈ 31 ps and follow a double-exponential rise-decay function, with rate constants as listed in Table 2 and S2. Here, the rate constants of the rise are in the range 1/50–1/60 ps^–1^, which is, within the error of the fits, equivalent to the relaxation rate of the **COn** modes. The rate constants of the decay are consistently 1/19 ps^–1^, i.e. slightly faster than for *c*_B_. Like in the case of *c*_B_, we attempted describing *c*_A,corr_ as a convolution of an exponential decay function with *k*_1_ = 1/58.8 ps^–1^ and a double-exponential rise-and-decay function according to eqs 2–4. Again, this approach results in overparametrization and indicates a direct energy transfer into the modes responsible for *S*_A_.

### Single-Frequency ppy-Pump/ppy-Probe Spectroscopy

Next, the nature of the underlying vibrationally excited states of **1** were examined. In this context, a question arises whether the probed modes **Py1/2** and **Ph1** are excited into their *v* = 1 states during the energy relaxation (*v*: vibrational quantum number). In general, significant populations of modes as high in energy as these are considered unlikely.^62,67,73^ To answer this question, we performed complementary single-frequency 2DIR and pump/probe measurements in the spectral region from 1450 cm^–1^ to 1620 cm^–1^ (see Section 5 in the Supporting Information for details). Indeed, these experiments corroborate the assumption that the population of the probed ppy-modes after **COn** excitation is minor. The vibrational relaxation of the **Py1** and **Py2** mode were found to proceed with double-exponential rate constants of *k*_1_ = 1/1.7 ps^–1^, 1/0.5 ps^–1^ (IVR) and *k*_2_ = 1/13 ps^–1^, 1/4.1 ps^–1^ (IET), respectively (Figure S6c). Since their relaxation is substantially quicker than that of both spectral components *S*_A_ and *S*_B_, the excited states of **Py1** and **Py2** cannot contribute significantly to either of them. In contrast, the relaxation of **Ph1** can be described single-exponentially with a rate constant of 1/21 ps^–1^, which is close to the relaxation rates of *S*_A_ and *S*_B_. However, probe spectra after **Ph1** excitation show transient absorptions of distinctly different line widths and peak position as compared to both spectral contributions *S*_A_ and *S*_B_ (Figure S6d). Thus, a notable population of the excited state of **Ph1** can be ruled out, too.

Interestingly, these experiments show that the relaxation of **1** after excitation of **Py1**, **Ph1** and **Py2** proceeds with very different rates. This finding implies that different sets of modes of lower frequency are populated depending on which of the three modes is pumped. However, a more detailed investigation of this finding exceeds the scope of the current work. Still, the match of the relaxation rates of *S*_A_ and *S*_B_ (**COn** excitation) and the single-frequency 2DIR spectra after **Ph1** excitation suggest that a substantial part of the energy of the excited **COn** modes is transferred to a similar subset of vibrational modes that gets populated from the excited **Ph1** state.

### DFT Anharmonic Frequency Calculations

Since all other anharmonic coupling constants of the complex are not known experimentally, we turned to anharmonic frequency calculations based on DFT to inform further analysis. Although it has been shown that such calculations return accurate coupling constants for the CO stretching modes of **1** even when a low-level DFT model is employed,^53^ their accuracy for other pairs of vibrational modes has yet to be examined. In a first step, the predicted and experimental couplings between the pumped **COn** and the probed **Py1/2** and **Ph1** modes are considered. The calculated frequency shifts, which are the negative of the off-diagonal anharmonicity constants, are listed in Table 3.

**Table 3:** Calculated Anharmonic Frequency Shifts between the Metal Carbonyl Stretching Modes and High-Frequency Pyridinyl- and Phenyl-Ring Modes of **1** in cm^–1^ (BP86/def2-SVP)^a^

Δ*ν̃*/cm^–1^	**Py2** (1480)	**Ph1** (1578)	**Py1** (1604)
**CO2** (1990)	+0.097	–0.020	+0.089
**CO3** (1976)	+0.027	–0.046	+0.022
**CO4** (1932)	+0.018	–0.069	–0.006

a Numbers in parentheses are the experimental frequencies of the fundamental transitions in cm^–1^.

In fact, the signs of computed shifts reproduce the experimental findings: excitation of any **COn** mode is predicted to shift the **Py2** transition positively in frequency and the **Ph1** negatively. **Py1** is predicted to shift predominately positively, but less than **Py2**, and to experience a down-shift upon excitation of **CO4**. Moreover, all shifts are in absolute numbers smaller than the line widths of the probed transitions, so that the apparent shifts in a difference spectrum should be dictated by exactly those widths, as experimentally found.

As discussed in the previous section, the contribution of the spectrum characterized by this coupling pattern, called *S*_A_ here, to the 2DIR spectra seems to increase during the first 23 ps, which means that at least one state other than the **COn** states has to contribute to it. This is possible because any coupling smaller than the line width of the transition measured will cause a cross peak pair with an apparent separation equal to this line width. Hence, every state which shifts **Py1/2** and **Ph1** by a small amount into the same directions as the **COn** modes will give rise to a transient spectrum very similar to that of the excited **COn** modes (*S*_A_)–potentially even indistinguishable, if the signal-to-noise ratio is low. Furthermore, if the magnitudes of the couplings between this state and **Py1/2** and **Ph1** are larger than in the case of **COn**, it will cause stronger cross peaks, although its population might be small. This is because the amplitude of cross peaks scales nearly proportionally with the anharmonic coupling in the limit of weak couplings.

Figure 5 provides a graphical summary of the calculated anharmonic couplings of **Py1**, **Ph1** and **Py2**, respectively, with all other modes of **1** (ignoring C–H stretching modes; values are listed in Table S4). As the figure demonstrates, there is indeed a group of vibrational modes that is predicted to have couplings with the same signs but greater magnitudes toward the measured modes: The low-frequency modes with frequencies below 250 cm^–1^ (**LF**). Even though the accuracy of the theoretical description of low-frequency modes is in general questionable,^92^ we want to point out here that the results just described are broadly consistent over different computational models (different density functionals, basis sets, presence or absence of dispersion correction and solvation model; see Supporting Information, Section 6.1 for a detailed comparison).

**Figure 5:**
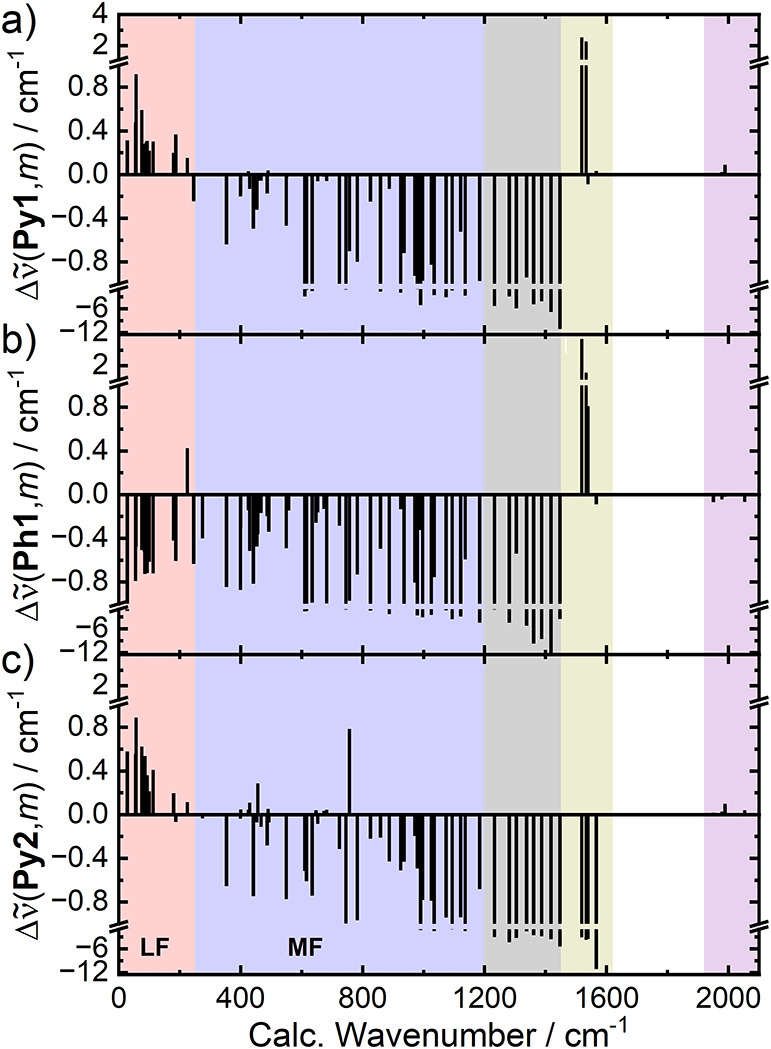
Calculated anharmonic frequency shifts of (a) the **Py1** band, (b) the **Ph1** band and (c) the **Py2** band upon excitation of other vibrational modes, *m*, of **1** (BP86/def2-SVP). These are equivalent to the negative coupling constants between *m* and either **Py1/2** or **Ph1**, respectively. Each bar represents a mode, *m*, its position on the abscissa its calculated fundamental frequency and its height the predicted anharmonic shift. Note that the scale of the ordinates changes at each indicated break. Colored areas emphasize groups of vibrational modes: violet—pumped **COn** modes, yellow—probed modes **Py1/2** & **Ph1**, gray—ppy-modes with strong coupling toward **Py1/2** & **Ph1**, blue—“medium-frequency” modes, red—“low-frequency” modes.

At this point, it might seem counterintuitive that the relaxation of the **LF** modes proceeds with a rate of only 1/19 ps^–1^, as their spectral overlap with the continuum of solvent modes should facilitate a faster energy transfer (see following section). However, it has to be kept in mind that the thermal energy of the solvent bath at room-temperature also enables an energy back transfer to the **LF** modes of **1**. The extracted decay constant of 1/19 ps^–1^ is therefore better viewed as the rate of a thermal equilibration process.

A similar assignment of the predominant spectral component, *S*_B_, to a small group of modes is unfeasible. Nearly all modes absorbing between 250 cm^–1^ and 1200 cm^–1^ (medium-frequency, **MF**) are predicted to cause negative shifts of all three measured bands of a few wavenumbers and, therefore, could possibly contribute to *S*_B_. Solely modes in the range 1200–1450 cm^–1^ are found to exhibit couplings notably larger than 3 cm^–1^ in most cases and, thus are unlikely to contribute to *S*_B_. This finding fulfils the expectation, that modes high in frequency are not significantly populated at late waiting times, as discussed in the previous section.

Although the calculations do not allow determination of the energy of the modes giving rise to *S*_B_ more precisely, they do allow implications regarding their spatial localization to be deduced. To illustrate this, to every vibrational mode *m* we assigned a fractional localization parameter ϕ_*m*_(ppy), which quantifies the contribution of the atoms of the ppy-ligand, *i*, to the total vibration. This parameter is calculated from the elements of the displacement vector, *d*, via

$$\phi_{m}\left(ppy\right)=\overset{N\left(ppy\right)}{\underset{i}{\sum }}{d_{x}\left(m,i\right)}^{2}+{d_{y}\left(m,i\right)}^{2}+{d_{z}\left(m,i\right)}^{2}$$ 5

where *N*(ppy) is the number of atoms of the ppy ligand. (The localization factor on the MCO moiety is accordingly ϕ_*m*_(MCO) = 1 – ϕ_*m*_(ppy).) The relation of ϕ_*m*_(ppy) and the couplings to the **Py1/2** and **Ph1** modes is depicted in Figure 6. As a general observation, modes with ϕ­(ppy) < 95%, i.e. with at least a small contribution of the atoms of the MCO moiety, exhibit a similar coupling pattern as the **COn**, albeit with higher absolute values. This includes the **LF** modes, all of which are strongly delocalized, as well as **MF** modes with high contribution of MCO-bending and M-(CO)-stretching modes, which absorb at frequencies in the range 400–700 cm^–1^. On the other hand, only modes essentially fully ppy-localized are predicted to give rise to anharmonic shifts of negative sign and appreciable magnitude to all three probed modes.

**Figure 6:**
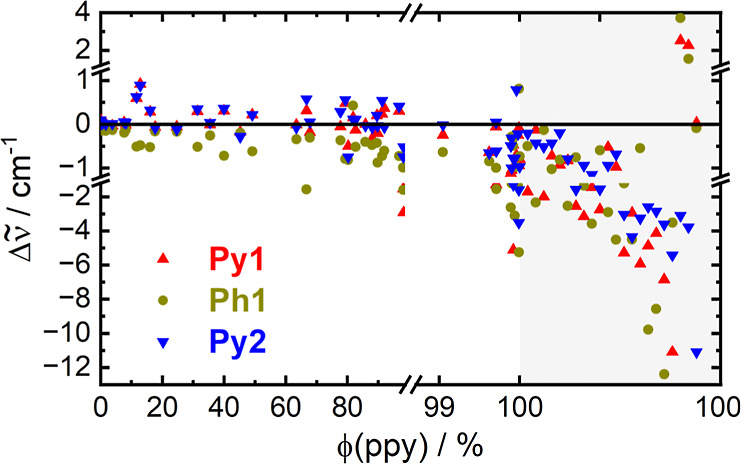
Calculated anharmonic frequency shifts of the **Py1/2** and **Ph1** bands (see legend) as a function of the ppy-localization factor defined according to eq 5 (BP86/def2-SVP). All data points in the gray area correspond to a localization factor of 100% and are only horizontally offset for the sake of comprehensibility.

Hence, only these ppy-localized modes will contribute to the 2DIR spectra in a manner consistent with the frequency shifts extracted from *S*_B_ (ca. −2.5 cm^–1^ shift of **Py1/2** and **Ph1**). In reverse, to explain the anharmonic frequency down-shifts of all three measured signals and an amplification factor of 4.7, in spite of the fact that the population of the underlying modes is always small because their rate of decline is greater than the rate of their rise, we need to assume that energy is indeed transferred from the MCO-groups to the ppy-ligand.

### Density of Acceptor States

Now that the intramolecular energy transfer CO → ppy at Mn within complex **1** has been proven, it remains to address the connection between this process and the higher vibrational relaxation rate as compared to the homoleptic complex [Mn_2_(CO)_10_], **2**. Even though fast population of **LF** modes seems to be an essential part of the relaxation, it cannot be held responsible for the increase in rate in **1**, since **2** has, according to our DFT calculations, more vibrational modes with frequencies <250 cm^–1^ (13 in **1** vs 20 in **2**). A reasonable explanation can be given based on Fermi’s Golden Rule, which states that the decay rate of an excited state is proportional to the density of states at the transition frequency and the square of the average coupling constant between donor and acceptor states.^101^ Even though this rule was originally formulated for energy transfer into a continuum of states, it has been shown to be a reasonably accurate approximation in systems with discrete states, given that the spacing of these states is small enough.^102^ Moreover, the donor and the acceptor state are not required to be exactly in resonance in the case of a solute, because an energy mismatch within the thermal energy can be balanced out by energy transfer to or from the solvent bath and low-frequency modes.^103,104^ Hence, the more states within a complex that have similar energies to the CO stretching modes, the faster the depopulation of these will be. For the following argument, we define arbitrarily a range of +50 cm^–1^ above the highest energy CO stretching mode and −100 cm^–1^ below the lowest one (using the calculated values) as being “close” to these modes. There are no fundamental modes in this range in either of these complexes. However, combination modes and overtones need to be considered, too. Here, to exemplify the difference in the density of states in both complexes, we only count states of a sum of all vibrational quantum numbers, ∑*v*, of two and three. The former are first overtones and combinations of two fundamental transitions, the latter second overtones, combinations of three fundamental transitions or of a first overtone and another fundamental transition.^105,106^

In **2**, the next modes lower in energy than the CO stretching modes are the MCO bending modes in the range from 690 cm^–1^ downward. As a consequence, there are no states with ∑*v* = 2 in the considered range either. The number of states with ∑*v* = 3 amounts to 596. In contrast, in **1**, there are 277 and 6854 states with a ∑*v* of two and three in this range, respectively, due to the ppy-centered modes having fundamental frequencies from 1605 cm^–1^ downward. Thus, our results point toward the increased relaxation rate of **1** compared to **2** as being attributable to the much higher density of acceptor states within the thermally accessible energy range around the MCO donor modes. This effect overcompensates the fact that the coupling between each MCO- and ppy-centered state in the isolated complex **1** is small due to localization on different ligands separated by the central manganese atom.

## Conclusion

The essential results and findings of this study are summarized in Scheme 2. In brief, we investigated the intramolecular vibrational energy redistribution in the precatalyst complex [Mn­(ppy)­(CO)_4_], **1**, from the CO-stretching modes (**COn**, *n* = 2–4), absorbing between 1920 cm^–1^ and 2010 cm^–1^, to the 2-phenylpyridine ligand (ppy) using dual-frequency 2DIR spectroscopy. Signals of three modes consisting of C=C and C=N stretching and C–H bending motions (**Py1**, **Ph1** and **Py2**) in the range from 1470 cm^–1^ to 1610 cm^–1^ were employed as reporters. Despite their only moderate extinction coefficients, transient signals of excellent quality could be obtained.

**Scheme 2:**
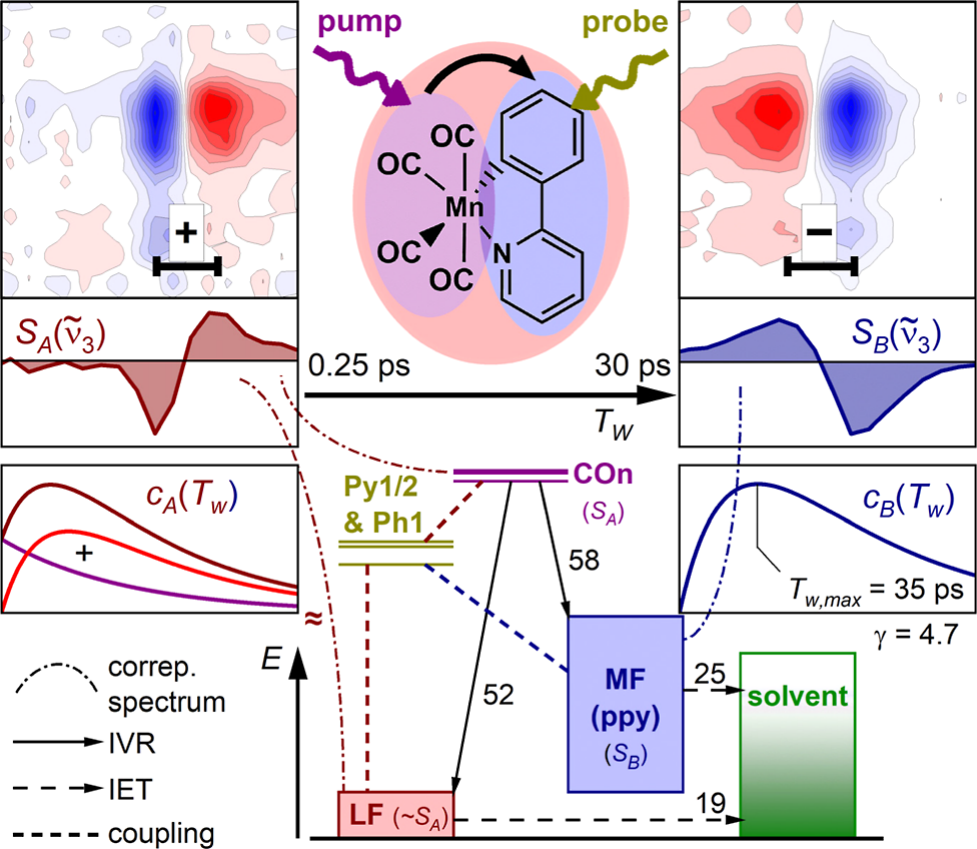
Graphical Summary of the Experiments Performed, the Spectra Obtained and the Mechanism Postulated in this Work. The Black Numbers Attached to the Arrows Refer to Time Constants in ps

The shape of the 2DIR spectra at early waiting times reflects the direct anharmonic coupling between the pumped and probed bands, which are strongly dependent on the probed but not on the pumped mode. In particular, the **Py2** band exhibits an uncommon, characteristic positive shift in frequency. At longer waiting times, these signals are outcompeted by a stronger, new set of transient absorptions with a frequency down-shift of −2.5 cm^–1^ common to all three ppy-centered modes. This second set of signals originate from coupling to other modes of the ppy-ligand lower in frequency, which are excited due to IVR from the CO modes. The energy transport time for the transfer CO → ppy in this complex was found to be (35.0 ± 0.7) ps, with an amplification factor of 4.7 ± 0.7.

Further analysis of the 2DIR spectra reveals that the transient absorptions originating from the initial coupling pattern do not decrease strictly exponentially with the rate of the depopulation of the CO stretching modes (1/58.8 ps^–1^), but instead increase initially. This finding is explained by a high population of low-frequency modes (**LF**, ν̃ < 250 cm^–1^) at early waiting times, which give rise to similar transient absorptions as the CO stretching modes. This assignment is supported by anharmonic frequency calculations based on DFT. In contrast, the experimental anharmonic frequency shifts observed at late waiting times are consistent with the DFT-predicted couplings to variety of modes with resonance frequency in the range 250–1200 cm^–1^ (medium-frequency, **MF**). These are predicted to exhibit an even stronger coupling, but with common, negative frequency shifts toward the three probed bands, if they do not involve a contribution of the metal carbonyl moiety >5%. Population of these modes results in more intense cross peaks because of their stronger coupling to the probed modes, so that their contribution is predominant at late waiting times. Hence, the difference in anharmonic couplings to the measured modes in this complex allows an independent evaluation of the population of two sets of vibrational modes. A kinetic analysis shows the populations of both **LF** and **MF** modes to be limited by the lifetime of the CO donor modes. We conclude that the energy transfer proceeds in a direct fashion, since no indications of intermediate states are found. This implies that the thermalization within the ppy-ligand via IVR between the ppy-centered modes is much faster than the IVR from the CO modes. The specific relaxation rate of the **LF** modes via IET to the solvent is found to be 1/19 ps^–1^. The remaining modes relax with a collective, average rate of 1/25 ps^–1^.

Referring back to the role of this complex in heterogeneous catalysis, the question arises whether the findings presented here allow for conclusions regarding its great catalytic applicability. This work highlighted the difference in the relaxation dynamics of homo- and heteroleptic metal carbonyl complexes. Yet, it represents only a single case study and, hence, it cannot on its own reveal a—most likely convoluted—correlation between vibrational structure and catalytic activity. For this reason, we anticipate that this work will inspire further research efforts in this field, which will ultimately provide the basis for extraction of a possible relation between energy distribution and catalytic activity.

## Supplementary Material



## Data Availability

The data underlying this study are openly available in Research Data York at https://doi.org/10.15124/336746d1-5756-4989-bd27-90667feba4c5.

## Notes

The synthesis of **1**, the 2DIR spectrometer, visualizations of all recorded dual-frequency 2DIR spectra, detailed results of spectral and kinetic fits and DFT calculations, DFT optimized geometries and single-frequency 2DIR and pump/probe spectra of **1** (PDF)

## †

Faculty of Science and Engineering, The University of Manchester, Oxford Rd, M13 9PL, Manchester, UK

## Abbreviations

**1**: [Mn­(ppy)­(CO)_4_]
**2**: [Mn_2_(CO)_10_]
2DIR: two-dimensional infrared
**3**: [MnBr­(CO)_5_]
**A**: hypothetical state
BJ: Becke–Johnson damping
BLYP: Becke’s 1988 exchange functional + the correlation functional of Lee, Yang, and Parr
BP86: Becke’s 1988 exchange functional + Perdew’s 1986 correlation functional
*c*_A_/*c*_A,corr_/*c*_B_: time-dependent coefficients of spectral components *S*_A_/*S*_B_
**COn**: carbonyl stretching modes of 1 with *n* = 1–4
CPCM: conductor-like polarizable continuum model
D3: Grimme’s empirical dispersion correction (version 3)
def2-SVP: redefinition of Ahlrich’s double-ζ basis set with polarization functions
def2-TZVP: redefinition of Ahlrich’s triple-ζ basis set with polarization functions
DFT: density functional theory
eq: equation
ϕ: localization factor
fwhm: full width at half-maximum
γ: amplification factor
GSB: ground state bleaching
IET: intermolecular energy transfer
IR: infrared
IVR: intramolecular vibrational energy redistribution
L: organic ligand(s)
**LF**: low-frequency
*m*: vibrational mode
MCO: metal carbonyl
**MF**: medium-frequency
mIR: mid-infrared
PBE: 1996 functional of Perdew, Burke and Ernzerhof
ppy: cyclometalated 2-phenylpyridine
ppyH: 2-phenylpyridine
PTFE: polytetrafluoroethylene
**Ph1**: phenyl C=C stretching mode of 1 (1587 cm^–1^)
**Py1**: pyridinyl C=C stretching mode of 1 (1604 cm^–1^)
**Py2**: pyridinyl C–H bending mode of 1 (1480 cm^–1^)
RA: relaxation assisted
*S*_A_/*S*_B_: spectral components
SI: Supporting Information
SVD: singular value decomposition
TA: transient absorption
TRIR: time-resolved infrared
*T*_w,max_: energy transport time
*v*: vibrational quantum number
W06: Ahlrich’s auxiliary basis set for density fitting
*Y*: plane of polarization perpendicular to Z
*Z*: plane of polarization

## References

[1] Peng J.-B., Geng H.-Q., Wu X.-F. (2019). The Chemistry of CO: Carbonylation. Chem.

[2] Das D., Bhanage B. M. (2020). Double Carbonylation
Reactions: Overview and Recent
Advances. Adv. Synth. Catal..

[3] Tomar V., Kumar P., Nemiwal M., Joshi R. K. (2023). Review
on Catalytic
Significance of 3d-Transition Metal-Carbonyl Complexes for General
and Selective Organic Reactions. Inorg. Chem.
Commun..

[4] Beller M., Cornils B., Frohning C. D., Kohlpaintner C. W. (1995). Progress
in Hydroformylation and Carbonylation. J. Mol.
Catal. A: Chem..

[5] Franke R., Selent D., Börner A. (2012). Applied Hydroformylation. Chem. Rev..

[6] Budiman A. W., Nam J. S., Park J. H., Mukti R. I., Chang T. S., Bae J. W., Choi M. J. (2016). Review of Acetic Acid Synthesis from
Various Feedstocks Through Different Catalytic Processes. Catal. Surv. Asia.

[7] Askes S. H. C., Reddy G. U., Wyrwa R., Bonnet S., Schiller A. (2017). Red Light-Triggered
CO Release from Mn_2_(CO)_10_ Using Triplet Sensitization
in Polymer Nonwoven Fabrics. J. Am. Chem. Soc..

[8] Lu-Jie L., He Y., Yang Y., Guo J., Lu Z., Wang C., Zhu S., Zhu S.-F. (2023). Recent
Advances in Mn, Fe, Co, and Ni-Catalyzed Organic
Reactions. CCS Chem..

[9] Vogiatzis K. D., Polynski M. V., Kirkland J. K., Townsend J., Hashemi A., Liu C., Pidko E. A. (2019). Computational
Approach to Molecular Catalysis by 3d
Transition Metals: Challenges and Opportunities. Chem. Rev..

[10] Wrighton M. (1974). Photochemistry
of Metal Carbonyls. Chem. Rev..

[11] Turner J. J., George M. W., Poliakoff M., Perutz R. N. (2022). Photochemistry of
Transition Metal Carbonyls. Chem. Soc. Rev..

[12] Ulloa O. A., Huynh M. T., Richers C. P., Bertke J. A., Nilges M. J., Hammes-Schiffer S., Rauchfuss T. B. (2016). Mechanism of H_2_ Production
by Models for the [NiFe]-Hydrogenases: Role of Reduced Hydrides. J. Am. Chem. Soc..

[13] Fairlamb I. J. S., Lynam J. M. (2024). Unveiling Mechanistic Complexity in Manganese-Catalyzed
C–H Bond Functionalization Using IR Spectroscopy Over 16 Orders
of Magnitude in Time. Acc. Chem. Res..

[14] Hammarback L. A., Bishop A. L., Jordan C., Athavan G., Eastwood J. B., Burden T. J., Bray J. T. W., Clarke F., Robinson A., Krieger J.-P., Whitwood A., Clark I. P., Towrie M., Lynam J. M., Fairlamb I. J. S. (2022). Manganese-Mediated
C–H Bond
Activation of Fluorinated Aromatics and the Ortho-Fluorine Effect:
Kinetic Analysis by In Situ Infrared Spectroscopic Analysis and Time-Resolved
Methods. ACS Catal..

[15] Hammarback L. A., Robinson A., Lynam J. M., Fairlamb I. J. S. (2019). Mechanistic
Insight
into Catalytic Redox-Neutral C–H Bond Activation Involving
Manganese­(I) Carbonyls: Catalyst Activation, Turnover, and Deactivation
Pathways Reveal an Intricate Network of Steps. J. Am. Chem. Soc..

[16] George M. W., Dougherty T. P., Heilweil E. J. (1996). UV Photochemistry of [CpFe­(CO)_2_]_2_ (Cp = η^5^-C_5_H_5_) Studied by Picosecond Time-Resolved Infrared Spectroscopy. J. Phys. Chem..

[17] Lomont J. P., Nguyen S. C., Harris C. B. (2013). Insights into the Photochemical Disproportionation
of Transition Metal Dimers on the Picosecond Time Scale. J. Phys. Chem. A.

[18] Marhenke J., Massick S. M., Ford P. C. (2007). Time-Resolved Infrared Study of Reactive
Species Produced by Flash Photolysis of the Hydroformylation Catalyst
Precursor Co_2_(CO)_6_(PMePh_2_)_2_. Inorg. Chim. Acta.

[19] Peters J., George M. W., Turner J. J. (1995). Photochemistry
of [CpMo­(CO)_3_]_2_. Direct Detection and Kinetics
of the Radical CpMo­(CO)_3_ in *n*-Heptane
Solution at Room Temperature
by Fast Time-Resolved Infrared Spectroscopy. Organometallics.

[20] Moskovich S., Reuvenov D., Schultz R. H. (2006). Microsecond UV Flash Photolysis of
Co_2_(CO)_8_ in Solution: Wavelength Dependence
of the Co­(CO)_4_/Co_2_(CO)_7_ Branching
Ratio. Chem. Phys. Lett..

[21] Watkins W. C., Jaeger T., Kidd C. E., Fortier S., Baird M. C., Kiss G., Roper G. C., Hoff C. D. (1992). An Investigation
of the Homolytic Dissociation of [η^5^-C_5_Me_5_Cr­(CO)_3_]_2_ and Related Complexes.
The Role of Ligand Substitution on the Solution Thermochemistry of
Metal-Metal Bond Cleavage. J. Am. Chem. Soc..

[22] Sun X. Z., Nikiforov S. M., Dedieu A., George M. W. (2001). Photochemistry of
[CpMo­(CO)_3_]_2_ (Cp = η^5^-C_5_H_5_) and [Cp*Fe­(CO)_2_]_2_ (Cp*
= η^5^-C_5_Me_5_) in Supercritical
CO_2_: A Fast Time-Resolved Infrared Spectroscopic Study. Organometallics.

[23] Owrutsky J. C., Baronavski A. P. (1996). Ultrafast Infrared Study of the Ultraviolet Photodissociation
of Mn_2_(CO)_10_. J. Chem.
Phys..

[24] Steinhurst D. A., Baronavski A. P., Owrutsky J. C. (2002). Transient Infrared Spectroscopy of
Mn_2_(CO)_10_ with 400 nm Excitation. Chem. Phys. Lett..

[25] Eastwood J. B., Burden T. J., Hammarback L. A., Horbaczewskyj C., Tanner T. F. N., Clark I. P., Greetham G., Towrie M., Fairlamb I. J. S., Lynam J. M. (2024). The Importance of
Understanding (Pre)­Catalyst
Activation in Versatile C–H Bond Functionalisations Catalysed
by [Mn_2_(CO)_10_]. Chem.
Sci..

[26] Firth J. D., Hammarback L. A., Burden T. J., Eastwood J. B., Donald J. R., Horbaczewskyj C. S., McRobie M. T., Tramaseur A., Clark I. P., Towrie M., Robinson A., Krieger J.-P., Lynam J. M., Fairlamb I. J. S. (2021). Light-
and Manganese-Initiated Borylation
of Aryl Diazonium Salts: Mechanistic Insight on the Ultrafast Time-Scale
Revealed by Time-Resolved Spectroscopic Analysis. Chem. Eur J..

[27] Szymańska A., Nowicki M., De S., Krupa B., Szyling J., Ležaić K., Moret M.-E., Walkowiak J. (2025). Catalytic
Hydroboration of Ketones Using Mn­(CO)_5_Br and Mn_2_(CO)_10_. Adv. Synth. Catal..

[28] Li T., Albert S. C. (2023). Chan; Shan-Shui
Meng. Recent Progress of Decacarbonyldimanganese
Catalysis. Chem. Synth..

[29] González T., García J. J. (2021). Catalytic CO2 Hydrosilylation with [Mn­(CO)_5_Br] under Mild Reaction Conditions. Polyhedron.

[30] Vivien A., Veyre L., Mirgalet R., Camp C., Thieuleux C. (2023). MnBr­(CO)_5_: A Commercially Available Highly
Active Catalyst for Olefin
Hydrosilylation under Ambient Air and Green Conditions. Green Chem..

[31] Schlichter P. W. (2021). Christophe.
The Rise of Manganese-Catalyzed Reduction Reactions. Synthesis.

[32] Gilbert B. C., Kalz W., Lindsay C. I., McGrail P. T., Parsons A. F., Whittaker D. T. E. (1999). Radical Cyclisations Promoted by Dimanganese Decacarbonyl:
A New and Flexible Approach to 5-Membered N-Heterocycles. Tetrahedron Lett..

[33] Weng W.-Z., Liang H., Liu R.-Z., Ji Y.-X., Zhang B. (2019). Visible-Light-Promoted
Manganese-Catalyzed Atom Transfer Radical Cyclization of Unactivated
Alkyl Iodides. Org. Lett..

[34] McMahon C. M., Renn M. S., Alexanian E. J. (2016). Manganese-Catalyzed
Carboacylations
of Alkenes with Alkyl Iodides. Org. Lett..

[35] Friestad G. K., Qin J. (2001). Intermolecular Alkyl
Radical Addition to Chiral N-Acylhydrazones
Mediated by Manganese Carbonyl. J. Am. Chem.
Soc..

[36] Gilbert B.
C., Kalz W., Lindsay C. I., McGrail P. T., Parsons A. F., Whittaker D. T. E. (2000). Initiation
of Radical Cyclisation Reactions Using Dimanganese
Decacarbonyl. A Flexible Approach to Preparing 5-Membered Rings. Perkin 1 (2000).

[37] Wang L., Lear J. M., Rafferty S. M., Fosu S. C., Nagib D. A. (2018). Ketyl Radical
Reactivity via Atom Transfer Catalysis. Science.

[38] Liu W., Ackermann L. (2016). Manganese-Catalyzed
C–H Activation. ACS Catal..

[39] Burden T. J., Eastwood J. B., Fairlamb I. J. S., Lynam J. M. (2022). Manganese-Catalyzed
C-H Bond Activation and Functionalization, From Mechanism to Applications. Handbook of CH-Functionalization.

[40] Hu Y., Zhou B., Wang C. (2018). Inert C–H
Bond Transformations
Enabled by Organometallic Manganese Catalysis. Acc. Chem. Res..

[41] Cano R., Mackey K., McGlacken G. P. (2018). Recent
Advances in Manganese-Catalysed
C–H Activation: Scope and Mechanism. Catal. Sci. Technol..

[42] Zhou B., Chen H., Wang C. (2013). Mn-Catalyzed Aromatic C–H
Alkenylation with Terminal Alkynes. J. Am. Chem.
Soc..

[43] Eastwood J.
B., Hammarback L. A., Burden T. J., Clark I. P., Towrie M., Robinson A., Fairlamb I. J. S., Lynam J. M. (2023). Understanding Precatalyst
Activation and Speciation in Manganese-Catalyzed C–H Bond Functionalization
Reactions. Organometallics.

[44] Hammarback L. A., Robinson A., Lynam J. M., Fairlamb I. J. S. (2019). Delineating the
Critical Role of Acid Additives in Mn-Catalysed C–H Bond Functionalisation
Processes. Chem. Commun..

[45] Hammarback L. A., Eastwood J. B., Burden T. J., Pearce C. J., Clark I. P., Towrie M., Robinson A., Fairlamb I. J. S., Lynam J. M. (2022). A Comprehensive
Understanding of Carbon–Carbon Bond Formation by Alkyne Migratory
Insertion into Manganacycles. Chem. Sci..

[46] Hammarback L. A., Clark I. P., Sazanovich I. V., Towrie M., Robinson A., Clarke F., Meyer S., Fairlamb I. J. S., Lynam J. M. (2018). Mapping
out the Key Carbon–Carbon Bond-Forming Steps in Mn-Catalysed
C–H Functionalization. Nat. Catal..

[47] Hammarback L. A., Aucott B. J., Bray J. T. W., Clark I. P., Towrie M., Robinson A., Fairlamb I. J. S., Lynam J. M. (2021). Direct Observation
of the Microscopic Reverse of the Ubiquitous Concerted Metalation
Deprotonation Step in C–H Bond Activation Catalysis. J. Am. Chem. Soc..

[48] Eastwood J. B., Hammarback L. A., McRobie M. T., Clark I. P., Towrie M., Fairlamb I. J. S., Lynam J. M. (2020). Time-Resolved Infra-Red Spectroscopy
Reveals Competitive Water and Dinitrogen Coordination to a Manganese­(I)
Carbonyl Complex. Dalton Trans..

[49] Pollak C., Rosa A., Baerends E. J. (1997). Cr–CO
Photodissociation in
Cr­(CO)_6_: Reassessment of the Role of Ligand-Field Excited
States in the Photochemical Dissociation of Metal–Ligand Bonds. J. Am. Chem. Soc..

[50] Paterson M. J., Hunt P. A., Robb M. A., Takahashi O. (2002). Non-Adiabatic
Direct Dynamics Study of Chromium Hexacarbonyl Photodissociation. J. Phys. Chem. A.

[51] Ziegler T., Tschinke V., Ursenbach C. (1987). Thermal Stability and Kinetic Lability
of the Metal Carbonyl Bond. A Theoretical Study on M­(CO)_6_ (M = Chromium, Molybdenum, Tungsten), M­(CO)_5_ (M = Iron,
Ruthenium, Osmium), and M­(CO)_4_ (M = Nickel, Palladium,
Platinum). J. Am. Chem. Soc..

[52] Spirina I. V., Maslennikov V. P. (1994). Thermal, Photolytic, and Oxidative
Reactions of the
Homoligand Carbonyls of Metals in Groups VI-VIII. Russ. Chem. Rev..

[53] Eastwood J. B., Procacci B., Gurung S., Lynam J. M., Hunt N. T. (2024). Understanding
the Vibrational Structure and Ultrafast Dynamics of the Metal Carbonyl
Precatalyst [Mn­(ppy)­(CO)_4_]. ACS Phys.
Chem. Au.

[54] Farmer A. L., Procacci B., Shaw D. J., Gurung S., Fairlamb I. J. S., Lynam J. M., Hunt N. T. (2025). Ultrafast
Vibrational Spectroscopic
Analysis of the Ubiquitous Precatalyst [Mn_2_(CO)_10_] in Different Solvents. J. Chem. Phys..

[55] Heilweil E. J., Cavanagh R. R., Stephenson J. C. (1987). Population Relaxation of CO­(v = 1)
Vibrations in Solution Phase Metal Carbonyl Complexes. Chem. Phys. Lett..

[56] Delor M., Sazanovich I. V., Towrie M., Spall S. J., Keane T., Blake A. J., Wilson C., Meijer A. J. H. M., Weinstein J. A. (2014). Dynamics of Ground and Excited State Vibrational Relaxation
and Energy Transfer in Transition Metal Carbonyls. J. Phys. Chem. B.

[57] Anna J. M., King J. T., Kubarych K. J. (2011). Multiple Structures and Dynamics
of [CpRu­(CO)_2_]_2_ and [CpFe­(CO)_2_]_2_ in Solution Revealed with Two-Dimensional Infrared Spectroscopy. Inorg. Chem..

[58] Hill A. D., Zoerb M. C., Nguyen S. C., Lomont J. P., Bowring M. A., Harris C. B. (2013). Determining Equilibrium Fluctuations Using Temperature-Dependent
2D-IR. J. Phys. Chem. B.

[59] Yan S., Seidel M. T., Zhang Z., Leong W. K., Tan H.-S. (2011). Ultrafast
Vibrational Relaxation Dynamics of Carbonyl Stretching Modes in Os_3_(CO)_12_. J. Chem. Phys..

[60] Feng M., Yang F., Wang J. (2016). Vibrational and Structural Dynamics
of Mn­(CO)_5_Br and Re­(CO)_5_Br Examined Using Nonlinear
Infrared Spectroscopy†. Chin. J. Chem.
Phys..

[61] Heilweil E. J., Cavanagh R. R., Stephenson J. C. (1988). CO­(v =
1) Population Lifetimes of
Metal–Carbonyl Cluster Compounds in Dilute CHCl_3_ Solution. J. Chem. Phys..

[62] Kasyanenko V. M., Tesar S. L., Rubtsov G. I., Burin A. L., Rubtsov I. V. (2011). Structure
Dependent Energy Transport: Relaxation-Assisted 2DIR Measurements
and Theoretical Studies. J. Phys. Chem. B.

[63] Lin Z., Rubtsov I. V. (2012). Constant-Speed Vibrational
Signaling along Polyethyleneglycol
Chain up to 60-Å Distance. Proc. Natl.
Acad. Sci. U. S. A..

[64] Rubtsova N. I., Rubtsov I. V. (2013). Ballistic Energy Transport via Perfluoroalkane Linkers. Chem. Phys..

[65] Rubtsova N. I., Nyby C. M., Zhang H., Zhang B., Zhou X., Jayawickramarajah J., Burin A. L., Rubtsov I. V. (2015). Room-Temperature
Ballistic Energy Transport in Molecules with Repeating Units. J. Chem. Phys..

[66] Qasim L. N., Atuk E. B., Maksymov A. O., Jayawickramarajah J., Burin A. L., Rubtsov I. V. (2019). Ballistic Transport
of Vibrational
Energy through an Amide Group Bridging Alkyl Chains. J. Phys. Chem. C.

[67] Kasyanenko V. M., Lin Z., Rubtsov G. I., Donahue J. P., Rubtsov I. V. (2009). Energy Transport
via Coordination Bonds. J. Chem. Phys..

[68] Keating C. S., McClure B. A., Rack J. J., Rubtsov I. V. (2010). Mode Coupling Pattern
Changes Drastically Upon Photoisomerization in RuII Complex. J. Phys. Chem. C.

[69] Fedoseeva M., Delor M., Parker S. C., Sazanovich I. V., Towrie M., Parker A. W., Weinstein J. A. (2015). Vibrational
Energy Transfer Dynamics in Ruthenium Polypyridine Transition Metal
Complexes. Phys. Chem. Chem. Phys..

[70] Leong T. X., Collins B. K., Dey Baksi S., Mackin R. T., Sribnyi A., Burin A. L., Gladysz J. A., Rubtsov I. V. (2022). Tracking Energy
Transfer across a Platinum Center. J. Phys.
Chem. A.

[71] Shipp J. D., Fernández-Terán R. J., Auty A. J., Carson H., Sadler A. J., Towrie M., Sazanovich I. V., Donaldson P. M., Meijer A. J. H. M., Weinstein J. A. (2024). Two-Dimensional
Infrared Spectroscopy Resolves the Vibrational Landscape in Donor–Bridge–Acceptor
Complexes with Site-Specific Isotopic Labeling. ACS Phys. Chem. Au.

[72] Kurochkin D. V., Naraharisetty S. R. G., Rubtsov I. V. (2007). A Relaxation-Assisted
2D IR Spectroscopy
Method. Proc. Natl. Acad. Sci. U. S. A..

[73] Rubtsova N. I., Rubtsov I. V. (2015). Vibrational Energy
Transport in Molecules Studied by
Relaxation-Assisted Two-Dimensional Infrared Spectroscopy. Annu. Rev. Phys. Chem..

[74] Ward J. S., Lynam J. M., Moir J. W. B., Sanin D. E., Mountford A. P., Fairlamb I. J. S. (2012). A Therapeutically
Viable Photo-Activated Manganese-Based
CO-Releasing Molecule (Photo-CO-RM). Dalton
Trans..

[75] Frisch, M. J. ; Trucks, G. W. ; Schlegel, H. B. ; Scuseria, G. E. ; Robb, M. A. ; Cheeseman, J. R. ; Scalmani, G. ; Barone, V. ; Petersson, G. A. ; Nakatsuji, H. ; Li, X. ; Caricato, M. ; Marenich, A. V. ; Bloino, J. ; Janesko, B. G. ; Gomperts, R. ; Mennucci, B. ; Hratchian, H. P. ; Ortiz, J. V. ; Izmaylov, A. F. ; Sonnenberg, J. L. ; Williams-Young, D. ; Ding, F. ; Lipparini, F. ; Egidi, F. ; Goings, J. ; Peng, B. ; Petrone, A. ; Henderson, T. ; Ranasinghe, D. ; Zakrzewski, V. G. ; Gao, J. ; Rega, N. ; Zheng, G. ; Liang, W. ; Hada, M. ; Ehara, M. ; Toyota, K. ; Fukuda, R. ; Hasegawa, J. ; Ishida, M. ; Nakajima, T. ; Honda, Y. ; Kitao, O. ; Nakai, H. ; Vreven, T. ; Throssell, K. ; Montgomery, J. A., Jr. ; Peralta, J. E. ; Ogliaro, F. ; Bearpark, M. J. ; Heyd, J. J. ; Brothers, E. N. ; Kudin, K. N. ; Staroverov, V. N. ; Keith, T. A. ; Kobayashi, R. ; Normand, J. ; Raghavachari, K. ; Rendell, A. P. ; Burant, J. C. ; Iyengar, S. S. ; Tomasi, J. ; Cossi, M. ; Millam, J. M. ; Klene, M. ; Adamo, C. ; Cammi, R. ; Ochterski, J. W. ; Martin, R. L. ; Morokuma, K. ; Farkas, O. ; Foresman, J. B. ; Fox, D. J. . Gaussian 16, Revision C.02 2016..

[76] Becke A. D. (1988). Density-Functional
Exchange-Energy Approximation with Correct Asymptotic Behavior. Phys. Rev. A:At., Mol., Opt. Phys..

[77] Perdew J. P. (1986). Density-Functional
Approximation for the Correlation Energy of the Inhomogeneous Electron
Gas. Phys. Rev. B.

[78] Perdew J. P., Burke K., Ernzerhof M. (1996). Generalized
Gradient Approximation
Made Simple. Phys. Rev. Lett..

[79] Perdew J. P., Burke K., Ernzerhof M. (1997). Generalized
Gradient Approximation
Made Simple [Phys. Rev. Lett. 77, 3865 (1996)]. Phys. Rev. Lett..

[80] Lee C., Yang W., Parr R. G. (1988). Development
of the Colle-Salvetti
Correlation-Energy Formula into a Functional of the Electron Density. Phys. Rev. B.

[81] Miehlich B., Savin A., Stoll H., Preuss H. (1989). Results Obtained with
the Correlation Energy Density Functionals of Becke and Lee, Yang
and Parr. Chem. Phys. Lett..

[82] Schäfer A., Horn H., Ahlrichs R. (1992). Fully Optimized
Contracted Gaussian
Basis Sets for Atoms Li to Kr. J. Chem. Phys..

[83] Weigend F., Ahlrichs R. (2005). Balanced Basis Sets
of Split Valence, Triple Zeta Valence
and Quadruple Zeta Valence Quality for H to Rn: Design and Assessment
of Accuracy. Phys. Chem. Chem. Phys..

[84] Weigend F. (2006). Accurate Coulomb-Fitting
Basis Sets for H to Rn. Phys. Chem. Chem. Phys..

[85] Schäfer A., Huber C., Ahlrichs R. (1994). Fully Optimized
Contracted Gaussian
Basis Sets of Triple Zeta Valence Quality for Atoms Li to Kr. J. Chem. Phys..

[86] Grimme S., Antony J., Ehrlich S., Krieg H. (2010). A Consistent and Accurate
Ab Initio Parametrization of Density Functional Dispersion Correction
(DFT-D) for the 94 Elements H-Pu. J. Chem. Phys..

[87] Grimme S., Ehrlich S., Goerigk L. (2011). Effect of the Damping Function in
Dispersion Corrected Density Functional Theory. J. Comput. Chem..

[88] Barone V., Cossi M. (1998). Quantum Calculation of Molecular Energies and Energy Gradients in
Solution by a Conductor Solvent Model. J. Phys.
Chem. A.

[89] Cossi M., Rega N., Scalmani G., Barone V. (2003). Energies, Structures,
and Electronic Properties of Molecules in Solution with the C-PCM
Solvation Model. J. Comput. Chem..

[90] Dunlap B. I. (1983). Fitting
the Coulomb Potential Variationally in Xα Molecular Calculations. J. Chem. Phys..

[91] Dunlap B. I. (2000). Robust
and Variational Fitting: Removing the Four-Center Integrals from Center
Stage in Quantum Chemistry. J. Mol. Struct.:THEOCHEM.

[92] Sitkiewicz S. P., Zaleśny R., Ramos-Cordoba E., Luis J. M., Matito E. (2022). How Reliable
Are Modern Density Functional Approximations to Simulate Vibrational
Spectroscopies?. J. Phys. Chem. Lett..

[93] Ciano L., Fey N., Halliday C. J. V., Lynam J. M., Milner L. M., Mistry N., Pridmore N. E., Townsend N. S., Whitwood A. C. (2015). Dispersion,
Solvent and Metal Effects in the Binding of Gold Cations to Alkynyl
Ligands: Implications for Au­(I) Catalysis. Chem.
Commun..

[94] Herzberg, G. Molecular Spectra and Molecular Structure: Infrared and Raman Spectra of Polyatomic Molecules; Molecular Spectra and Molecular Structure; Van Nostrand: New York, 1945.

[95] Herzberg, G. Molecular Spectra and Molecular Structure: Infrared and Raman Spectra of Polyatomic Molecules. In Molecular Spectra and Molecular Structure; R.E. Krieger Publishing Company, 1991.

[96] Mulliken R.
S. (1955). Report
on Notation for the Spectra of Polyatomic Molecules. J. Chem. Phys..

[97] Tuttle W. D., Gardner A. M., Andrejeva A., Kemp D. J., Wakefield J. C. A., Wright T. G. (2018). Consistent Assignment of the Vibrations of Symmetric
and Asymmetric Ortho-Disubstituted Benzenes. J. Mol. Spectrosc..

[98] Hamm, P. ; Zanni, M. Concepts and Methods of 2D Infrared Spectroscopy; Cambridge University Press: Cambridge, 2011 10.1017/CBO9780511675935.

[99] Rubtsov I. V., Wang J., Hochstrasser R. M. (2003). Vibrational Coupling between Amide-I
and Amide-A Modes Revealed by Femtosecond Two Color Infrared Spectroscopy. J. Phys. Chem. A.

[100] Keating C. S., McClure B. A., Rack J. J., Rubtsov I. V. (2010). Sulfoxide
Stretching Mode as a Structural Reporter via Dual-Frequency Two-Dimensional
Infrared Spectroscopy. J. Chem. Phys..

[101] Orear, J. ; Fermi, E. ; Rosenfeld, A. ; Schluter, R. Nuclear Physics: A Course Given by Enrico Fermi at the University of Chicago. In Midway Reprints; University of Chicago Press, Chicago, 1950.

[102] Micklitz T., Morningstar A., Altland A., Huse D. A. (2022). Emergence
of Fermi’s Golden Rule. Phys. Rev. Lett..

[103] Bernshtein V., Oref I. (2006). Energy Transfer between
Polyatomic
Molecules II: Energy Transfer Quantities and Probability Density Functions
in Benzene, Toluene, p-Xylene, and Azulene Collisions. J. Phys. Chem. A.

[104] Kenkre V. M., Tokmakoff A., Fayer M. D. (1994). Theory of Vibrational
Relaxation of Polyatomic Molecules in Liquids. J. Chem. Phys..

[105] Stuchebrukhov A. A., Marcus R. A. (1993). Theoretical Study of Intramolecular
Vibrational Relaxation of Acetylenic CH Vibration for v = 1 and 2
in Large Polyatomic Molecules (CX_3_)_3_YCCH, Where
X = H or D and Y = C or Si. J. Chem. Phys..

[106] Rubtsov I. V., Burin A. L. (2019). Ballistic and Diffusive
Vibrational
Energy Transport in Molecules. J. Chem. Phys..

