# Supplementary material for: Intramolecular Vibrational Energy Transfer in the Precatalyst [Mn(ppy)(CO)4] Tracked by Dual-Frequency 2D Infrared Spectroscopy
Source: J Phys Chem A. 2026 May 18;130(21):4052–65. doi: 10.1021/acs.jpca.6c01573 (PMC13224191; doi:10.1021/acs.jpca.6c01573)
Supplement: Supplementary file 1 [file jp6c01573_si_001.pdf]

Intramolecular Vibrational Energy Transfer in the Precatalyst [Mn(ppy)$(CO)_4$] Tracked by Dual-Frequency 2D Infrared Spectroscopy – Supporting Information

Stefan Flesch,[*] Barbara Procacci, Sabina Gurung,[†] Shweta Choudhary, Ian J. S. Fairlamb, Jason M. Lynam, and Neil T. Hunt

Department of Chemistry, The University of York, Heslington, YO10 5DD, York, UK.

* Email: stefan.flesch@york.ac.uk; Telephone: +44(0) 1904 324525

[†] Current address: Faculty of Science and Engineering, The University of Manchester, Oxford Rd, M13 9PL, Manchester, UK.

Table of Contents

1. Synthesis S2

1.1 Step 1: Synthesis of [Mn(Bn)$(CO)_5$] S2

1.2 Step 2: Synthesis of [Mn(ppy)$(CO)_4$] S2

2. Experimental Setup S3

3. Dual-Frequency 2DIR Spectra S3

3.1 All Dual-Frequency 2DIR Spectra S3

3.2 Singular Value Decomposition S5

4. Kinetic Modelling S6

4.1 Fits of the Estimated Spectra S_A and S_B S6

4.2 CO-Pump/ppy-Probe Spectroscopy S7

4.3 Kinetic Fits of c_A and c_B S7

4.4 Alternative Kinetic Modelling S9

5. Single-Frequency ppy-Pump/ppy-Probe Spectroscopy S12

6. DFT-Calculations S14

6.1 Calculated Anharmonic Frequency Shifts S14

6.2 Optimized Geometries S18

7. References S24

1. Synthesis

1.1 Step 1: Synthesis of [Mn(Bn)(CO)$_5$]

4.16 g (10.7 mmol, 1 eq.) [$Mn_2(CO)_{10}$] was dissolved in 80 mL of dry, deoxygenated tetrahydrofuran (THF) in a nitrogen-purged Schenk tube. 12 mL of mercury were put into a separate Schenk tube under nitrogen. 1.18 g sodium metal (42.8 mmol, 4 eq.) was added in small portions under rapid stirring to the mercury in the second tube. Then, the THF solution from the first tube was transferred via cannula to the sodium amalgam, and the mixture was stirred further for 3 hours. Subsequently, 2.47 ml benzyl chloride (21.4 mmol, 2 eq.) was added to a separate, third Schlenk tube under nitrogen and placed in an ice bath. This Schlenk tube was put under vacuum while stirring for 30 seconds, and afterwards refilled with nitrogen. The THF solution was transferred via cannula filtration to the benzyl chloride at room temperature. The solution was stirred for 20 hours, thereafter filtered through a layer of Celite® and washed with diethyl ether. The crude material was first purified by flash column chromatography (silica gel, petrol). Finally, after removal of benzyl chloride impurities under reduced pressure at 35°C, the product, [Mn(Bn)(CO)$_5$], was obtained as a pale green crystalline solid (4.11 g, 67%). Mp 40–41°C (reference [1] 40°C); R_f 0.33 (petrol); ^{1}H NMR (500 MHz, $CDCl_3$, δ / ppm): 7.24– 7.12 (m, 4H), 7.05–6.94 (m, 1H); ^{13}C NMR (126 MHz, $CDCl_3$, δ / ppm): 212.4, 210.0, 151.9, 128.8, 126.0, 123.6, 11.3; LIFDI-MS m/z (ion, %) 286 $[M]^+$; IR (CH_2Cl_2, solution, $\tilde{\nu}$ / cm^{-1}): 2940, 2874, 2857, 2107, 2011, 1990, 1598, 1489, 1124. The analytical data obtained were in accordance with the literature.[1]

1.2 Step 2: Synthesis of [Mn(ppy)(CO)$_4$]

2.50 g [MnBn(CO)$_5$] (9.15 mmol, 1 eq.), 1.33 ml 2-phenylpyridine (9.15 mmol, 1 eq.) and 60 mL dry, deoxygenated hexane were added to a Schlenk tube under nitrogen. The reaction mixture was heated to reflux and stirred under exclusion from light for 24 hours. Subsequently, the reaction mixture was cooled to room temperature and was filtered through a cotton plug. Any product that precipitated out of solution was dissolved in a small amount of CH_2Cl_2. The solvent was removed under reduced pressure, redissolved in acetonitrile and washed with hexane. The solvent was removed under reduced pressure to yield the product. The crude material was purified by flash column chromatography (hexane, 100%) to afford the product, [Mn(ppy)(CO)$_4$], **1**. Mp 114–115 °C (reference [1] 114°C); R_f 0.26 (petrol/CH_2Cl_2, 8:2, v/v); ^{1}H NMR (500 MHz, $CDCl_3$, δ / ppm): 8.00 (d, J = 7.5 Hz, 1H), 7.89 (d, J = 8.0 Hz, 1H), 7.84–7.74 (m, 2H), 7.29 (dd, J = 7.5, 7.5 Hz, 1H), 7.19 (dd, J = 7.5, 7.5 Hz, 1H), 7.12 (dd, J = 6.5, 6.5 Hz, 1H); ^{13}C NMR (126 MHz, $CDCl_3$, δ / ppm): 220.2, 214.3, 214.1, 174.9, 166.5, 154.0, 146.3, 141.8, 137.9, 130.4, 124.2, 124.1, 122.5, 119.4; LIFDI-MS m/z (ion, %): 321 ($[M]^+$,100); IR (CH_2Cl_2, solution, $\tilde{\nu}$ / cm^{-1}): 2075, 1990, 1976, 1932, 1604, 1578, 1480. The analytical data obtained were in accordance with the literature.[1]

2. Experimental Setup

The 2DIR spectrometer employed in this work consisted of two Yb-based amplified lasers (Pharos 20W and Pharos 10W, Light Conversion) synchronized by a single, common oscillator. The outputs of both lasers were frequency-down converted each by an optical parametric amplifier (OPA, Orpheus Mid-IR, Light Conversion) followed by a difference frequency generation (DFG, Lyra, Light Conversion) to create independently tuneable mIR pulses with bandwidths >200 cm^{-1}, energies of 2.6 and 1.4 μJ/pulse (pump and probe, respectively) at a pulse repetition rate of 50 kHz. Here, the center frequencies of the pulses were set to either 1550 cm^{-1} or 2000 cm^{-1}. For data collection, a 2DQuick spectrometer (Phasetech) was employed. The spectrometer operates under pump-probe geometry,[2–4] utilizes a pulse shaper to set the time delay, τ, between the two pump pulses and an optical delay stage to adjust the waiting time, T_w, between the second pump and the probe pulse. τ was incremented with a step size of 20 fs up to a maximum of 4 ps applying a rotating frame frequency of 1585 cm^{-1} for excitation of the CO modes, or 24 fs, 2.544 ps and 1208 cm^{-1}, respectively, for excitation of the ppy modes. The intensity spectrum of the probe pulse was recorded on a 64-pixel HgCdTe detector. The relative polarization of the pump with regard to the probe pulse field was set via a half-wave plate and a polarizer.

3. Dual-Frequency 2DIR Spectra

3.1 All Dual-Frequency 2DIR Spectra

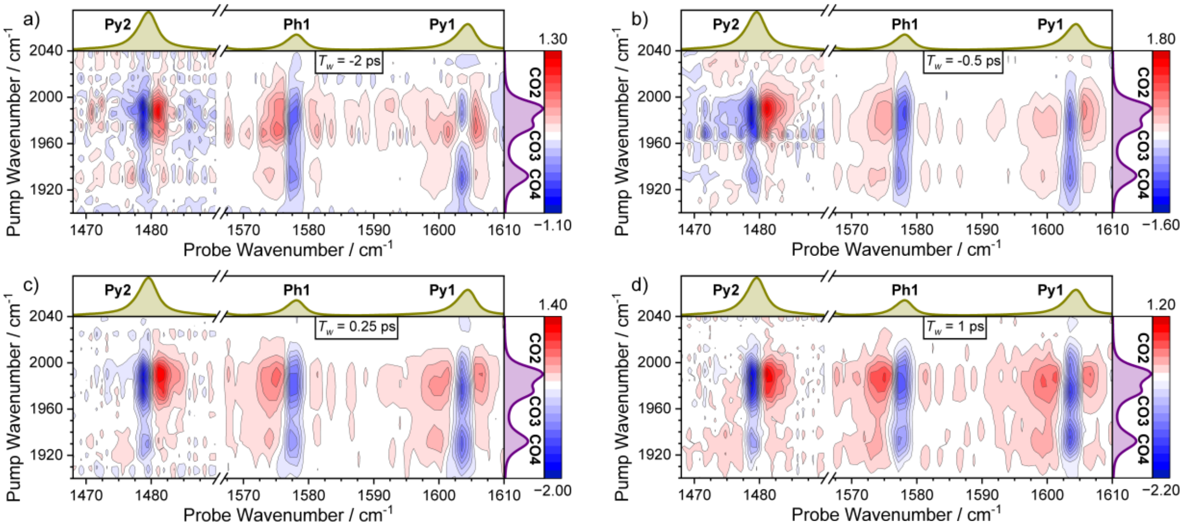

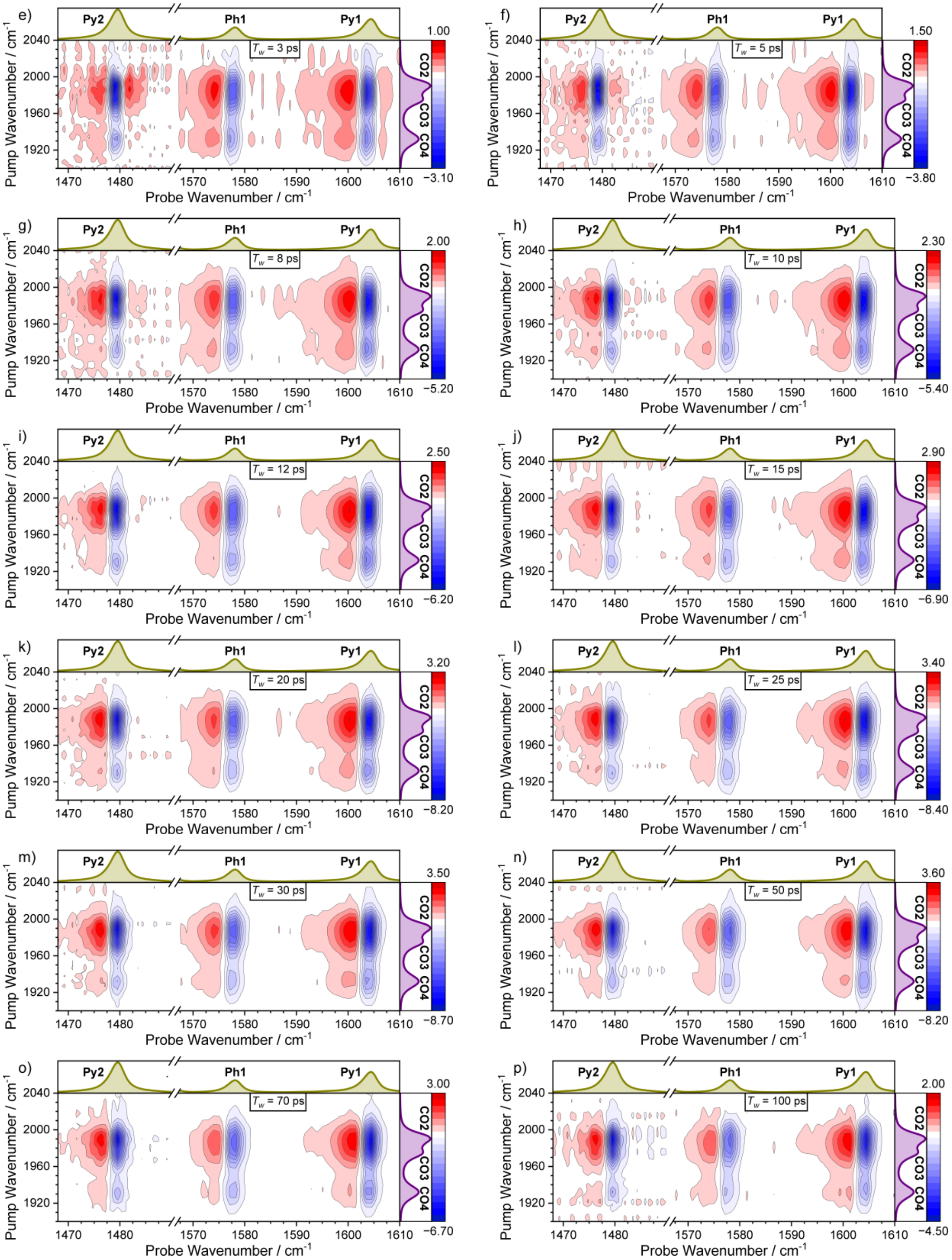

Figure S1. Dual-frequency 2DIR spectra of **1** in room-temperature CH_2Cl_2 solution recorded at the waiting times as specified in the labels and under (ZZYY) pulse polarization. The violet and yellow spectra attached to the right and upper borders are the stationary absorption spectrum of **1** in the displayed pump and probe region, respectively.

3.2 Singular Value Decomposition

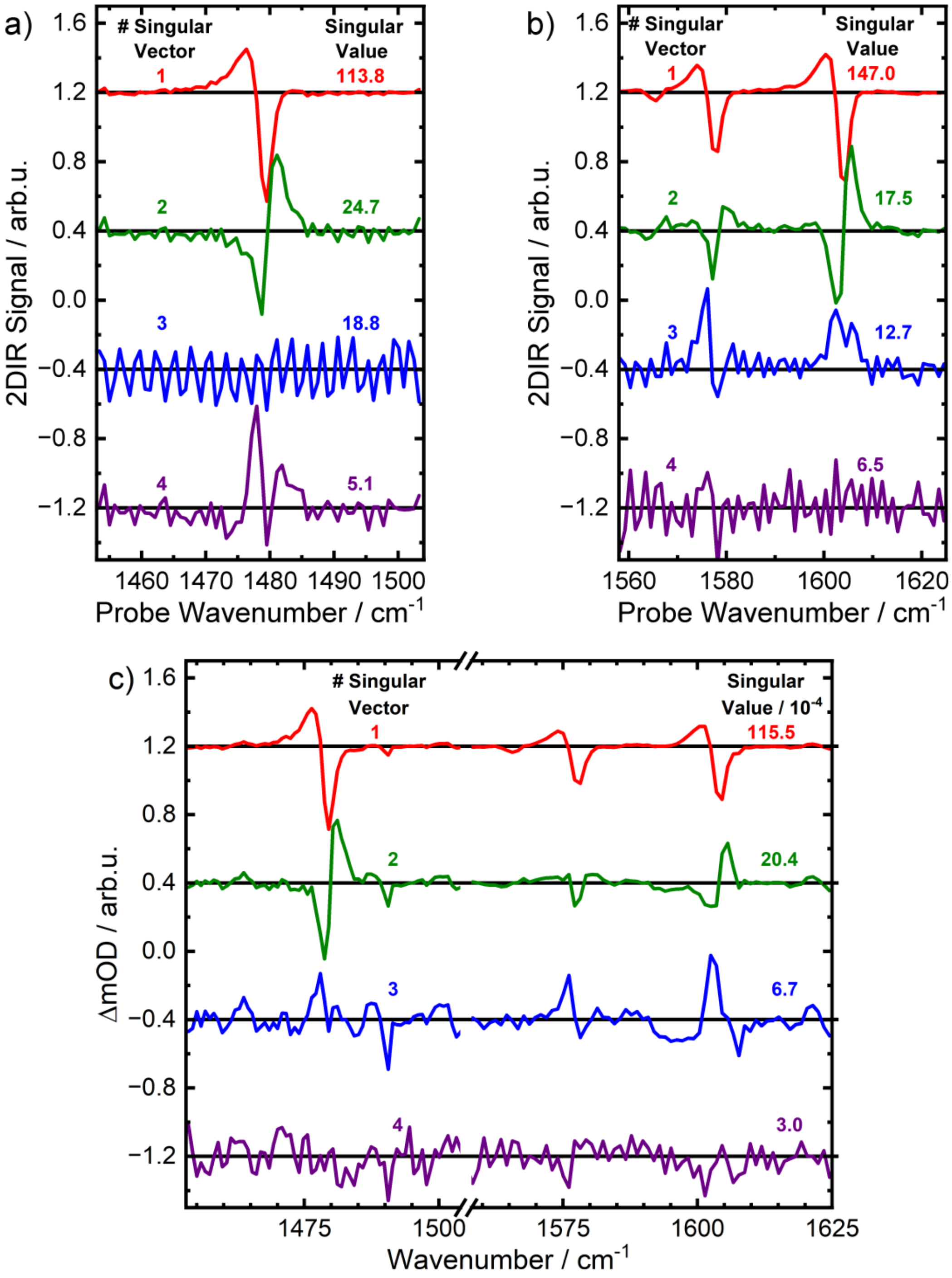

Figure S2. Left singular vectors obtained from a singular value decomposition of all 2DIR probe spectra with pump frequencies of 1920 cm^{-1} < $\tilde{\nu}_1$ < 2010 cm^{-1} in a) the **Py2** and b) the **Ph1** and **Py1** probe region. c) Left singular vectors obtained from a singular value decomposition of the CO-pump/ppy-probe spectra. The numbers on the left-hand side are the number of the singular vector in decreasing order of corresponding singular values. The singular values are given on the right hand-side.

4. Kinetic Modelling

4.1 Fits of the Estimated Spectra S_A and S_B

$$S_{A/B}(\tilde{\nu}_3) = \sum_{i=1}^{6} \frac{2a_i w_i}{\pi} \cdot \frac{1}{4\cdot(\tilde{\nu}_3 - \tilde{\nu}_{3,0i})^2 + w_i^2} \qquad \text{(S1)}$$

Table S1. Optimized parameters of hextuple-Lorentzian fit functions (eq. S1) describing the estimated transient spectra S_A and S_B.

	S_A			S_B		
I	a_i / arb.u.	w_i / cm^{-1}	$\tilde{\nu}_{3,0i}$ / cm^{-1}	a_i / arb.u.	w_i / cm^{-1}	$\tilde{\nu}_{3,0i}$ / cm^{-1}
1	1.88	2.93	1481.3	-3.51	3.96	1479.7
2	-1.68	1.77	1478.9	3.37	5.55	1477.4
3	4.84	4.72	1576.9	-1.22	3.37	1577.6
4	-4.18	2.75	1577.5	0.71	3.62	1573.9
5	331.9	3.66	1603.6	-2.29	2.71	1604.2
6	-333.1	3.62	1603.6	1.77	5.06	1600.7

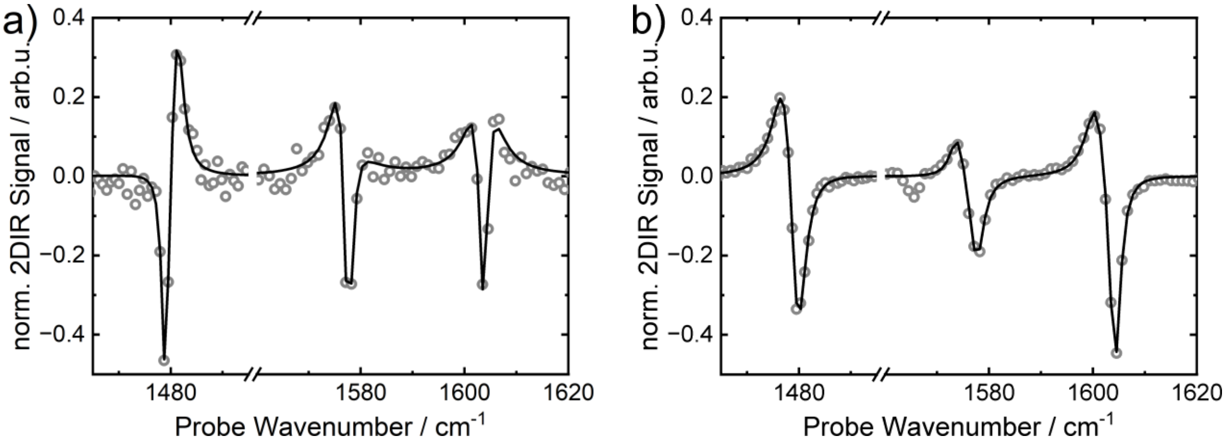

Figure S3. Estimated, square-normalized transient spectra a) S_A and b) S_B. Symbols represent raw spectra and lines hextuple-Lorentzian fit functions (eq. S1).

4.2 CO-Pump/ppy-Probe Spectroscopy

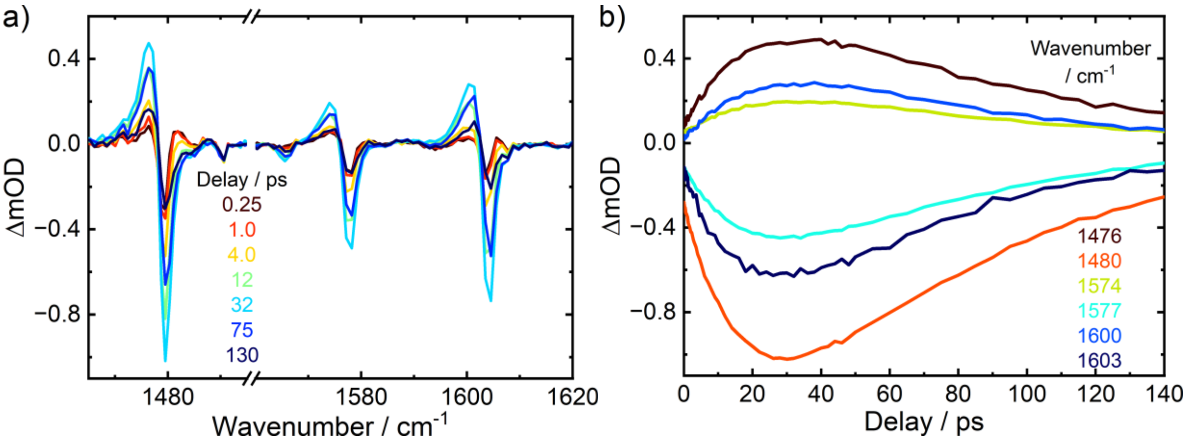

Figure S4. a) Dual-frequency pump/probe spectra of **1** in CH_2Cl_2 solution with excitation in the CO-stretching region of the spectrum and detection in the ppy-ring mode region, at selected pump/probe delays as mentioned in the legend. b) Kinetic traces of the same experiments at selected wavenumbers.

4.3 Kinetic Fits of c_A and c_B

We note that the evolution of c_A cannot be explained under the assumption that S_A originates from only a single state, **A**, other than the **COn** modes. **A** cannot be excited by the pump pulse directly, either because it is not resonant with the pump pulse or, if it is resonant, because it is a dark state. Only an IR active transition in the spectral range from 1900 cm^{-1} to 2020 cm^{-1} can be excited directly in this experiment. However, such a transition would also manifest itself as an additional signal in the stationary IR absorption spectrum, which is not observed.

Hence, **A** had to be populated in an ultrafast fashion from the excited **COn** modes to cause the nonzero signal strength at $T_w = 0$. If that was the case and this initial transfer was irreversible, the second slower energy transfer on the ps timescale necessary to explain the posterior rise of c_A was impossible, as the **COn** modes were already depopulated at that point. If the initial transfer was reversible, on the other hand, the equilibrium between **COn** and **A** would be reached immediately. In that case, any further energy transfer to **A** *via* a different, slower channel would not increase its population either, because the excess energy would be transferred back to **COn** quickly to restore the equilibrium.

Thus, we conclude that c_A comprises the evolution of two different (sets of) states: First, the **COn** modes, whose population is nonzero at $T_w = 0$, decays single-exponentially and gives rise to the spectrum S_A as

originally defined. Second, another (set of) states, whose population is zero at $T_w = 0$, but increases initially and causes spectral features very similar to S_A, so that the fitting procedure described in the main text returns a rise of S_A.

Table S2. Parameters of double-exponential rise-decay functions optimized to describe the time-dependent coefficients c_A and c_B in dependence on the pump frequency.

$\tilde{\nu}_1$ / cm^{-1}	$A_1(c_B)$ / arb.u.	$1/k_1(c_B)$ / ps	$A_2(c_B)$ / arb.u.	$1/k_2(c_B)$ / ps	$A_1(c_{A,corr})$ / arb.u.	$1/k_1(c_{A,corr})$ / ps	$A_2(c_{A,corr})$ / arb.u.	$1/k_2(c_{A,corr})$ / ps
1921.3	13.7±1.0	52[a]	-12.9±1.0	25.0±1.5	4.4±3.6	48.3±17.4	-4.4±3.6	20.8±7.5
1924.0	16.8±1.0	52[a]	-15.8±1.0	24.9±1.2	4.4±2.0	53.9±13.8	-4.3±2.0	19.1±4.9
1926.8	19.3±1.0	52[a]	-18.3±1.0	25.0±1.1	4.4±1.4	58.5±12.1	-4.3±1.3	17.8±3.6
1929.6	21.6±1.0	52[a]	-20.5±1.0	25.2±1.0	5.0±1.5	58.3±11.2	-4.9±1.5	18.3±3.4
1932.3	25.6±19.6	49.5±12.4	-24.5±19.6	26.6±6.6	5.9±2.4	54.1±11.8	-5.8±2.4	20.2±4.3
1935.1	20.9±11.1	53.3±11.4	-19.8±11.1	25.1±5.3	8.1±6.5	47.3±13.9	-8.0±6.5	23.4±6.8
1937.9	15.6±6.1	58.3±11.6	-14.6±6.0	23.0±4.5	5.4±0.3	54.0±3.1	-5.4±0.3	20 [a]
1940.6	11.7±3.5	65.1±12.4	-10.7±3.4	21.0±4.0	5.1±0.3	52.5±3.2	-5.1±0.4	20 [a]
1943.4	8.9±1.9	72.5±13.0	-8.0±1.9	18.3±3.2	4.6±0.3	53.7±3.7	-4.6±0.4	20 [a]
1946.2	6.9±1.1	82.4±13.7	-6.2±1.0	15.3±2.5	3.9±0.3	56.7±4.4	-3.9±0.3	20 [a]
1948.9	6.7±1.0	82.0±13.1	-6.1±0.9	14.6±2.3	3.7±0.3	57.9±4.7	-3.6±0.3	20 [a]
1951.7	7.1±1.2	76.9±12.4	-6.6±1.1	15.3±2.4	3.7±0.3	55.6±4.6	-3.6±0.3	20[a]
1954.5	9.4±1.9	68.6±11.0	-8.8±1.8	17.6±2.7	5.0±4.0	49.6±17.5	-4.9±4.0	21.8±7.6
1957.2	12.0±2.6	64.4±9.7	-11.4±2.6	19.0±2.8	4.0±1.5	57.5±13.5	-3.9±1.5	18.1±4.2
1960.0	16.6±3.7	60.4±8.1	-16.0±3.7	20.5±2.6	4.7±1.3	59.1±11.0	-4.6±1.3	17.6±3.2
1962.8	22.3±5.1	57.2±6.9	-21.7±5.1	21.7±2.5	5.9±1.6	56.5±9.5	-5.9±1.6	18.4±3.0
1965.5	28.5±5.7	56.4±5.7	-27.9±5.6	22.1±2.1	8.1±2.3	52.9±8.1	-8.1±2.3	19.7±2.9
1968.3	33.9±5.4	56.8±4.7	-33.4±5.4	22.0±1.7	10.1±2.7	51.1±6.8	-10.1±2.7	20.1±2.6

1971.1	37.9±4.9	58.1±4.1	-37.6±4.9	21.7±1.4	11.5±2.6	50.9±5.9	-11.5±2.6	19.9±2.2
1973.8	40.2±4.4	59.9±3.7	-39.9±4.3	21.4±1.2	12.4±2.4	51.5±5.2	-12.5±2.4	19.6±1.9
1976.6	40.8±3.9	61.5±3.6	-40.6±3.9	21.1±1.1	13.5±2.7	51.0±5.0	-13.6±2.6	20.0±1.9
1979.3	43.2±4.0	61.7±3.4	-43.0±4.0	21.3±1.1	14.6±2.9	50.6±4.9	-14.7±2.8	20.3±1.9
1982.1	48.2±4.7	59.8±3.3	-48.2±4.7	21.9±1.1	14.1±2.4	51.7±4.6	-14.3±2.4	19.6±1.7
1984.9	53.8±5.6	58.2±3.1	-54.0±5.5	22.5±1.1	13.5±2.0	53.1±4.3	-13.7±1.9	18.8±1.5
1987.6	56.4±6.0	57.5±3.1	-56.9±6.0	22.8±1.1	13.4±2.0	53.0±4.4	-13.5±2.0	18.8±1.5
1990.4	56.2±5.9	57.8±3.1	-56.8±5.9	22.8±1.1	13.6±2.2	52.4±4.6	-13.8±2.2	19.4±1.6
1993.2	54.4±6.3	57.5±3.3	-55.0±6.2	23.0±1.2	13.4±2.7	51.2±5.1	-13.6±2.7	20.1±1.9
1995.9	50.1±6.4	57.7±3.7	-50.6±6.4	23.2±1.4	15.0±4.7	47.8±6.1	-15.2±4.6	21.9±2.7
1998.7	41.7±6.2	58.4±4.4	-42.1±6.2	23.2±1.6	13.8±5.6	46.8±7.3	-13.9±5.6	22.5±3.4
2001.5	30.6±5.0	61.4±5.6	-30.8±4.9	22.6±1.9	11.5±5.3	47.3±8.6	-11.5±5.3	22.4±3.9
2004.2	25.4±6.6	59.1±7.7	-25.5±6.6	23.8±2.8	8.5±4.2	48.4±10.1	-8.6±4.1	21.6±4.4
2007.0	20.2±0.5	58[a]	-20.2±0.5	24.7±0.5	5.7±3.0	49.7±12.4	-5.8±2.9	20.2±4.9
2009.8	15.5±0.5	58[a]	-15.5±0.5	25.1±0.7	3.4±1.3	56.4±14.7	-3.4±1.3	17.3±4.4
pump/ probe	9.9±0.4	55.7±0.9	-9.6±0.4	25.1±0.5	1.9±0.2	46.2±2.5	-1.8±0.2	19.0±0.7

[a] fixed value. Free optimization of all parameters results in unreasonably large standard errors

4.4 Alternative Kinetic Modelling

In addition to the general description of the dual-frequency 2DIR and pump/probe spectra as a superposition of two spectral components via equation 1 (main text), the spectra were modelled using enforced time-dependences of the components. In all following cases, c_B, the time evolution of S_B, is described using a double-exponential rise-and-decay function. In equation S2, c_A, the time-dependent coefficient of spectrum S_A, is forced to follow a mono-exponential decay function of with increasing waiting time T_w. This model was used a) with a fixed rate constant $k_A = 1/58.8\ ps^{-1}$ and b) with k_A as freely optimized parameter. Equation S3 allows a double-exponential evolution of c_A instead (as suggested by the results obtained from modelling via equation 1), with two freely optimized rate constants, $k_{A,1}$ and $k_{A,2}$. Again all free parameters and the spectrum S_B were optimized in an iterative fashion. Naturally, the quality of the models decreases in the order eq. 1 > eq. S3 > eq. S2b > eq. S2a, as the number of free parameters is continuously reduced.

$$S(\tilde{\nu}_1, \tilde{\nu}_3, T_w) = S_{\boldsymbol{A}}(\tilde{\nu}_3)\, A_{\boldsymbol{A}}(\tilde{\nu}_1)\, exp(-k_{\boldsymbol{A}}(\tilde{\nu}_1)\, T_w) + S_{\boldsymbol{B}}(\tilde{\nu}_3) \cdot \left[A_{\boldsymbol{B},1}(\tilde{\nu}_1)\, exp\left(-k_{\boldsymbol{B},1}(\tilde{\nu}_1)\, T_w\right) + A_{\boldsymbol{B},2}(\tilde{\nu}_1)\, exp\left(-k_{\boldsymbol{B},2}(\tilde{\nu}_1)\, T_w\right)\right] \quad \text{(S2)}$$

$$S(\tilde{\nu}_1, \tilde{\nu}_3, T_w) = S_{\boldsymbol{A}}(\tilde{\nu}_3) \cdot \left[A_{\boldsymbol{A},1}(\tilde{\nu}_1) exp\left(-k_{\boldsymbol{A},1}(\tilde{\nu}_1)\, T_w\right) + A_{\boldsymbol{A},2}(\tilde{\nu}_1)\, exp\left(-k_{\boldsymbol{A},2}(\tilde{\nu}_1)\, T_w\right)\right] + S_{\boldsymbol{B}}(\tilde{\nu}_3) \cdot \left[A_{\boldsymbol{B},1}(\tilde{\nu}_1)\, exp\left(-k_{\boldsymbol{B},1}(\tilde{\nu}_1)\, T_w\right) + A_{\boldsymbol{B},2}(\tilde{\nu}_1)\, exp\left(-k_{\boldsymbol{B},2}(\tilde{\nu}_1)\, T_w\right)\right] \quad \text{(S3)}$$

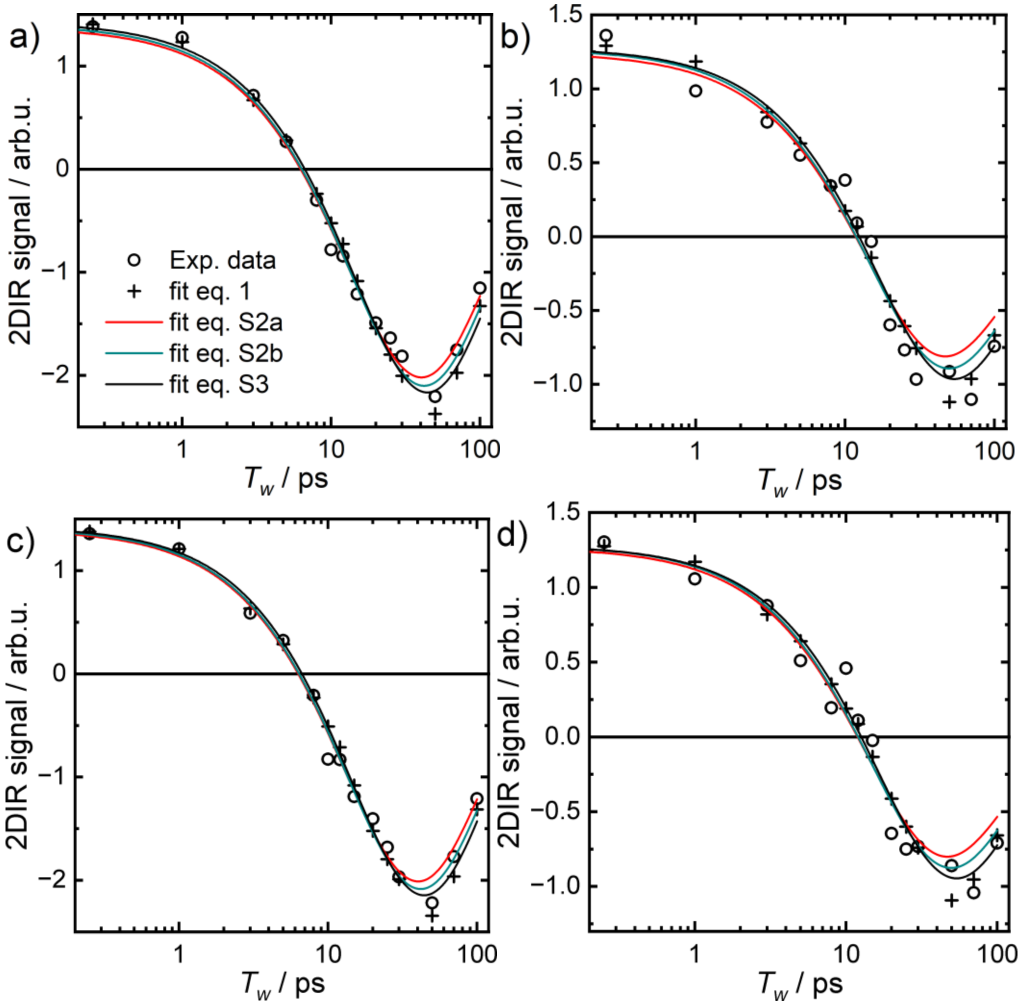

Figure S5. Kinetic traces of the experimental dual-frequency pump/probe spectra of 1 in CH_2Cl_2 solution and fitted points/curves based on equations 1, S2a, S2b and S3, as explained in the legend. Each subfigure represents data for a different combination of pump and probe frequency: a) $\tilde{\nu}_1$ = 1990 cm^{-1}, $\tilde{\nu}_3$ = 1481 cm^{-1}, b) $\tilde{\nu}_1$ = 1990 cm^{-1}, $\tilde{\nu}_3$ = 1482 cm^{-1}, c) $\tilde{\nu}_1$ = 1987 cm^{-1}, $\tilde{\nu}_3$ = 1481 cm^{-1}, d) $\tilde{\nu}_1$ = 1987 cm^{-1}, $\tilde{\nu}_3$ = 1482 cm^{-1}.

In order to illustrate the importance of the exponential rise function in the description of c_A, Fig. S5 displays a comparison of the experimental data and fit curves obtained from the different models. In detail, kinetic traces of the 2DIR spectra with $\tilde{\nu}_1$ = 1987 cm^{-1} or 1990 cm^{-1} and $\tilde{\nu}_3$ = 1481 cm^{-1} or 1482 cm^{-1}, *i.e.* in the region in which S_A is most distinguishable from S_B, are considered. As the figure demonstrates, the fit curves based on eq. S2a,b systematically underestimate the absolute 2DIR signal strength at both early

and late waiting times. In particular, the peak of the bleaching signal at ca. 30 ps is badly reproduced using a mono-exponential constrain of c_A. It might appear counterintuitive that the removal of the rise process of the spectral component with a positive contribution to the 2DIR spectra at these pump and probe frequencies causes a negative signal to be too weak. However, it has to be kept in mind that an optimization procedure will try to compensate for any inaccuracy in the description of c_A by adapting the parameters defining c_B and S_B to improve the overall quality of the fit. Moreover, since there are more data point available at early than at late waiting times, the fits can be expected to reproduce the points in the former range better than those in the latter.

Another argument against a mono-exponential description of c_A can be gathered from the rate constant k_A in eq. S2b. In fact, the optimized values of this parameter fall in the entire range from 1/38 ps^{-1} to 1/107 ps^{-1}, depending on $\tilde{\nu}_1$. Besides the inconsistency, values close to the expected value of 1/59 ps^{-1} are rare. Hence, the best possible model using a mono-exponential evolution of c_A contradicts the results of the CO-pump/CO-probe measurements. Therefore, we reject this model in favor of an alternative description allowing an increase of c_A.

5. Single-Frequency ppy-Pump/ppy-Probe Spectroscopy

2DIR spectra were recorded under (ZZZZ) and (ZZYY) polarization. Exemplary spectra from the measurements under (ZZYY) polarization are shown in the Fig. S6a,b. Pump/probe spectra and kinetic traces shown represent measurements under magic angle conditions. Pump and probe pulses of two different center frequencies were used: 1660 and 1550 cm^{-1}, for the higher- and lower-frequency spectral window, respectively. The 2DIR spectra were stitched together from these four independent measurements. Pump/probe spectra were only recorded with pump and probe pulses of identical center frequencies (*i.e.* two series of measurements).

Table S3. Anharmonic frequency shifts of the ppy-ring modes of **1** extracted from the single-frequency 2DIR spectra, and vibrational relaxation times obtained from the pump/probe measurements (c.f. Fig. S6).

	**Py2**	**Ph1**	**Py1**
$\Delta\tilde{\nu}$(**Py2**) / cm^{-1}, T_w = 0.25 ps	-1.6	-3.2	n.a.
$\Delta\tilde{\nu}$(**Ph1**) / cm^{-1}, T_w = 0.25 ps	-1.0	-2.8	-2.0
$\Delta\tilde{\nu}$(**Py1**) / cm^{-1}, T_w = 0.25 ps	n.a.	-0.3	-3.0
$\Delta\tilde{\nu}$(**Py2**) / cm^{-1}, T_w = 20 ps	-0.5	-2.2	-2.1
$\Delta\tilde{\nu}$(**Ph1**) / cm^{-1}, T_w = 20 ps	-0.8	-2.7	-2.0
$\Delta\tilde{\nu}$(**Py1**) / cm^{-1}, T_w = 20 ps	-0.6	-2.2	-2.1
τ_1 / ps	0.5	2.6	1.7
τ_2 / ps	4.1	21.2	13.0

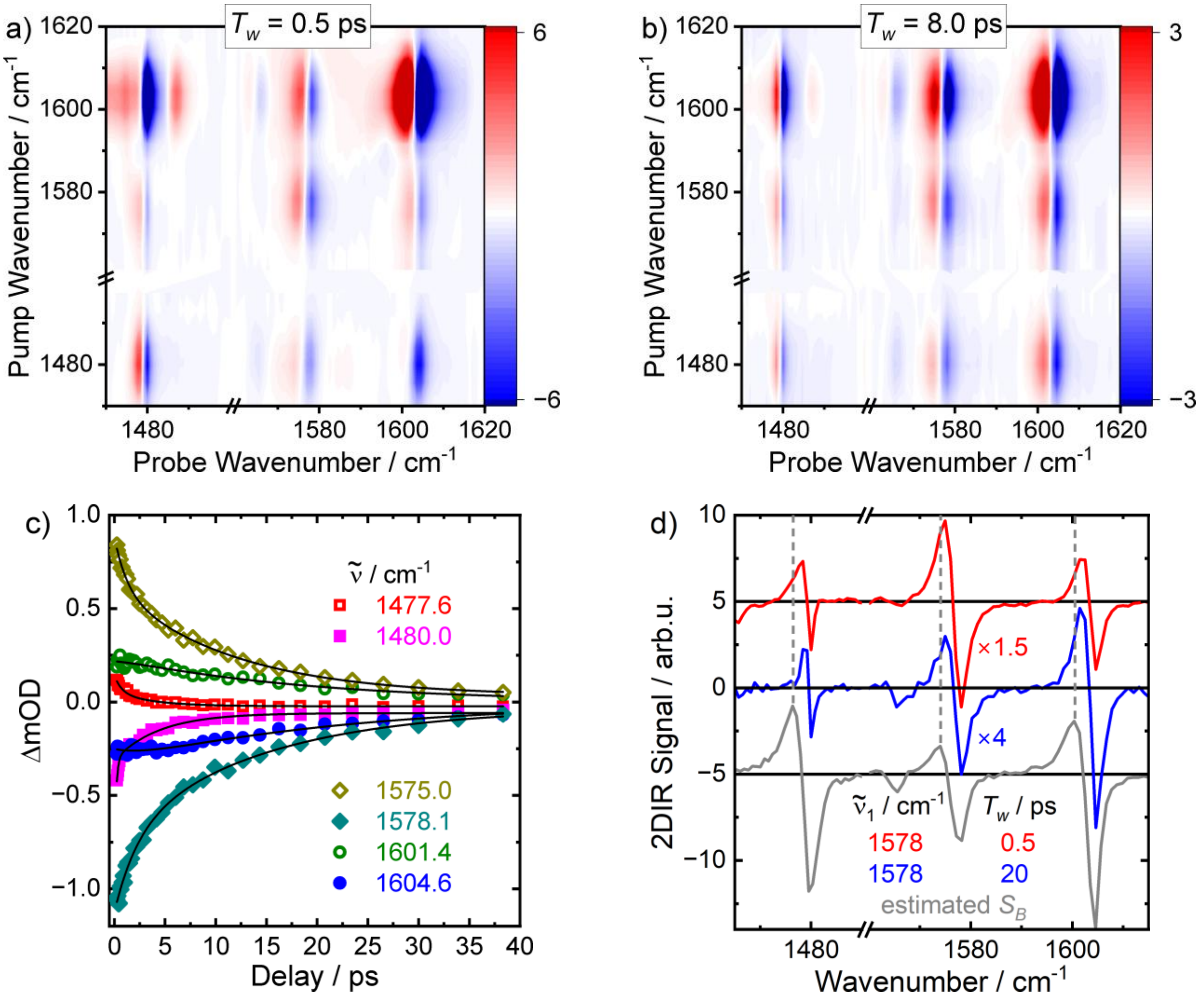

Figure S6. Single-frequency 2DIR spectra of **1** in CH_2Cl_2 solution in the spectral region from 1470 to 1620 cm^{-1} recorded at waiting times of a) 0.5 ps and b) 8.0 ps. c) IR-pump/IR-probe kinetic traces at selected probe frequencies as specified in the legend (symbols) and double-exponential fit functions (solid lines). d) Comparison of probe spectra from these experiments at two selected waiting times after **Ph1** excitation and the estimated spectrum S_B extracted from the dual-frequency 2DIR spectra after **COn** excitation presented in the main text. Dashed gray lines emphasize the peak positions of the transient absorptions from the latter spectrum.

6. DFT-Calculations

6.1 Calculated Anharmonic Frequency Shifts

Table S4. DFT-calculated, anharmonic frequencies of the vibrational modes, m, of **1**, anharmonic frequency shifts of the ppy-ring modes monitored in this work (BP86/def2-SVP), and localization of the vibrational modes on the 2-phenylpyridine and the metal carbonyl moiety (contributions determined from the squared displacement vectors).

$\tilde{\nu}$(anharm) / cm^{-1}	$\Delta\tilde{\nu}$(**Py1**,m) / cm^{-1}	$\Delta\tilde{\nu}$(**Ph1**,m) / cm^{-1}	$\Delta\tilde{\nu}$(**Py2**,m) / cm^{-1}	ppy-contribution / %	MCO-contribution / %
3012	-6.36	0.78	3.28	100.00	0.00
2972	-0.15	-0.20	-0.57	100.00	0.00
2996	4.05	-0.67	3.75	100.00	0.00
2980	-0.04	-7.47	0.25	100.00	0.00
2956	-2.95	-0.22	2.39	100.00	0.00
2989	0.09	5.11	1.66	100.00	0.00
2971	0.12	-0.65	0.86	100.00	0.00
2944	0.16	3.64	1.61	100.00	0.00
2053	-0.02	-0.07	0.04	0.15	99.85
1988	0.09	-0.02	0.10	0.02	99.98
1978	0.02	-0.05	0.03	0.06	99.94
1950	-0.01	-0.07	0.02	0.42	99.58
1566	0.03	-0.09	-11.09	100.00	0.00
1539	-0.09	0.81	-3.51	99.99	0.01
1533	2.27	1.55	-3.77	100.00	0.00
1519	2.52	3.71	-3.10	100.00	0.00
1447	-11.09	-3.51	-5.42	100.00	0.00
1418	-6.85	-12.39	-3.61	100.00	0.00
1387	-4.14	-8.57	-2.85	100.00	0.00
1361	-4.87	-9.78	-2.59	100.00	0.00
1338	-0.94	-5.26	-1.59	99.99	0.01
1304	-5.92	-0.54	-3.26	100.00	0.00
1281	-2.94	-4.50	-4.34	100.00	0.00
1233	-5.28	-1.21	-3.03	100.00	0.00
1184	-0.97	-4.51	-0.68	100.00	0.00
1122	-0.52	-2.90	-0.94	100.00	0.00
1136	-2.74	-0.59	-1.55	100.00	0.00
1094	-1.44	-3.57	-1.15	100.00	0.00
1074	-3.15	-1.34	-0.94	100.00	0.00
1035	-2.55	-0.76	-1.57	100.00	0.00
1025	-0.83	-2.53	-0.79	100.00	0.00
997	-0.97	-3.10	-0.78	99.94	0.06
990	-5.11	-0.33	-1.29	99.92	0.08
979	-1.12	-2.62	-0.49	99.89	0.11
971	-0.93	-0.81	-0.20	100.00	0.00
936	-0.72	-1.02	-0.43	100.00	0.00
925	-2.00	-0.13	-0.51	100.00	0.00
887	-0.13	-2.33	-0.43	100.00	0.00
859	-1.70	-0.50	-0.21	100.00	0.00
826	-0.25	-1.39	-0.22	99.98	0.02
782	-0.80	-0.73	-0.97	100.00	0.00
745	-1.20	-1.21	-1.00	99.90	0.10
724	-1.05	-0.29	-0.32	99.90	0.10
757	-0.70	-0.97	0.78	99.96	0.04

682	-0.06	-1.00	0.04	99.71	0.29
674	-0.02	-0.13	0.04	8.18	91.82
647	-0.01	-0.26	0.04	35.44	64.56
653	-0.06	-0.16	-0.09	17.60	82.40
634	-1.53	-0.99	-0.74	98.06	1.94
616	-1.29	-1.54	-0.61	99.71	0.29
610	-2.92	-1.60	-0.52	98.15	1.85
557	0.01	-0.15	0.00	1.62	98.38
550	-0.47	-0.49	-0.78	99.92	0.08
550	-0.04	-0.13	0.01	3.79	96.21
490	0.04	-0.19	0.05	7.63	92.37
492	-0.01	-0.34	-0.07	63.41	36.59
487	-0.17	-0.20	-0.28	45.19	54.81
467	-0.06	-0.17	-0.11	24.64	75.36
456	-0.06	-0.36	0.29	77.68	22.32
453	-0.32	-0.48	-0.07	88.03	11.97
441	-0.50	-0.82	-0.75	80.21	19.79
425	0.03	-0.15	0.04	0.09	99.91
429	-0.13	-0.52	0.11	82.65	17.35
400	-0.20	-0.87	-0.04	89.76	10.24
400	-0.18	-0.31	0.05	67.77	32.23
354	-0.64	-0.85	-0.65	99.62	0.38
275	0.00	-0.40	-0.04	85.81	14.19
246	-0.25	-0.64	-0.02	99.04	0.96
225	0.15	0.42	0.11	81.83	18.17
187	0.37	-0.61	-0.07	91.94	8.06
180	0.20	-0.42	0.20	89.57	10.43
113	0.30	-0.72	0.41	96.74	3.26
100	0.22	-0.62	0.21	49.11	50.89
90	0.29	-0.51	0.35	31.32	68.68
93	0.31	-0.72	0.36	39.93	60.07
84	0.28	-0.52	0.32	15.98	84.02
86	0.23	-0.73	0.54	91.27	8.73
76	0.59	-0.51	0.62	11.61	88.39
57	0.92	-0.48	0.89	12.81	87.19
55	0.48	-0.79	0.56	79.27	20.73
28	0.31	-1.57	0.58	66.66	33.34

In order to explore the effect of the details of the solvation model on the coupling constants, the calculations were repeated with parameters to model the solvents water, acetonitrile, methanol, acetone, toluene and heptane at the same density of integration points, and for CH_2Cl_2 using densities of integration points equivalent to 5.0, 10.0, 15.0, 20.0, 30.0, 40.0 and 50.0. The effect of the solvent (dielectric constant in the CPCM) on the coupling constants is shown in Fig. S7. Changing the average number of integration points of the CPCM surface (keyword: “PDens”) in the considered range has a vanishingly small effect on

the harmonicity constants. We found mean absolute deviations smaller than 0.006 cm^{-1} and root-mean-square deviations smaller than 0.022 cm^{-1} for each pair of calculations with different values of the "PDens" parameter.

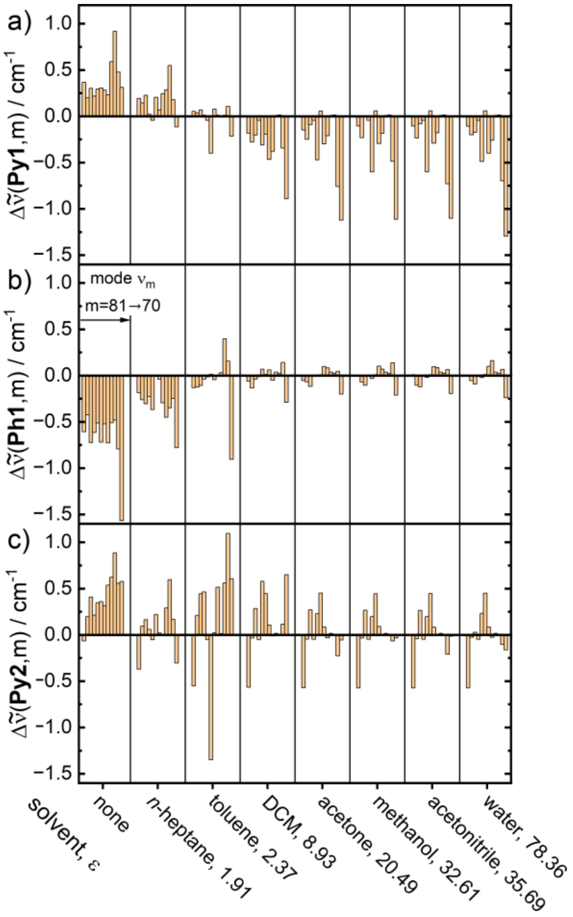

Figure S7. Calculated (BP86/def2-SVP) anharmonic frequency shifts of the a) **Py1**, b) **Ph1** and c) **Py2** mode upon excitation of low-frequency modes of **1** in dependence on the chosen solvent / dielectric constant, ε, in the CPCM settings. In each segment, the bars correspond to the normal modes 81-70 (numeration according to fundamental frequencies in decreasing order) from left to right.

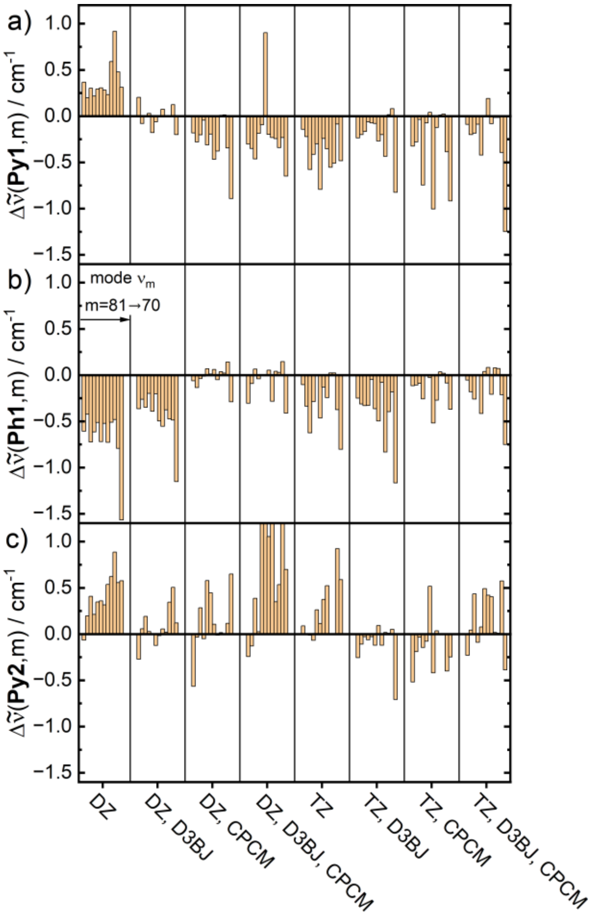

Figure S8. Calculated anharmonic frequency shifts of the a) **Py1**, b) **Ph1** and c) **Py2** upon excitation of low-frequency modes of **1** in dependence on the basis set, the presence of D3BJ dispersion correction and the presence of CPCM(CH_2Cl_2) solvent model using the BP86 density functional. In each segment, the bars correspond to the normal modes 81-70 (numeration according to fundamental frequencies in decreasing order) from left to right. ("DZ" = def2-SVP, "TZ" = def2-TZVP)

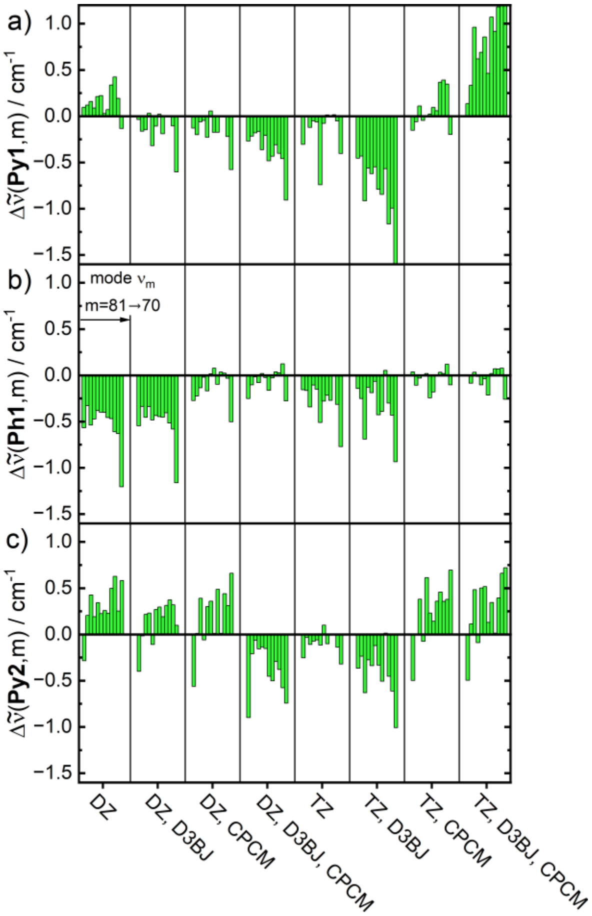

Figure S9. Calculated anharmonic frequency shifts of the a) **Py1**, b) **Ph1** and c) **Py2** mode upon excitation of low-frequency modes of **1** in dependence on the basis set, the presence of D3BJ dispersion correction and the presence of CPCM(CH_2Cl_2) solvent model using the PBE density functional. In each segment, the bars correspond to the normal modes 81-70 (numeration according to fundamental frequencies in decreasing order) from left to right. (“DZ” = def2-SVP, “TZ” = def2-TZVP)

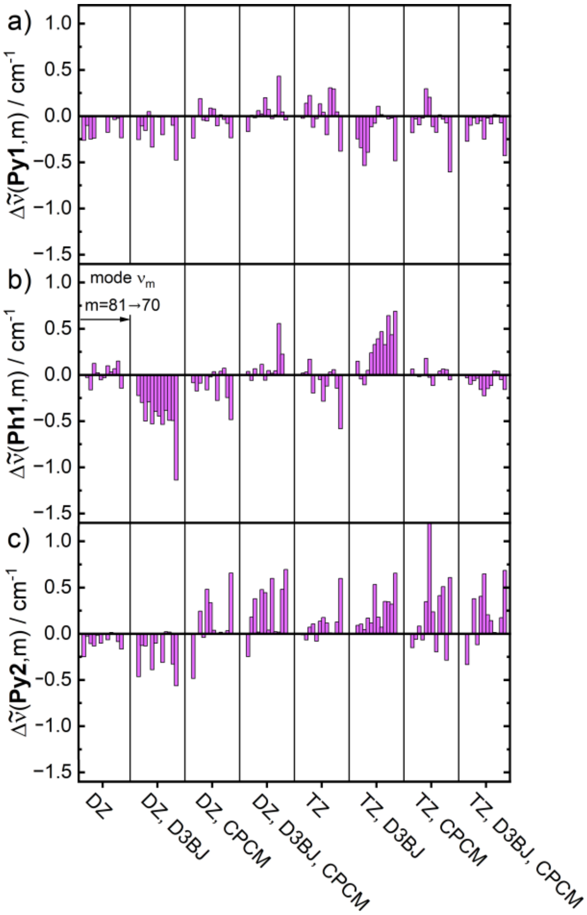

Figure S10. Calculated anharmonic frequency shifts of the a) **Py1**, b) **Ph1** and c) **Py2** mode upon excitation of low-frequency modes of **1** in dependence on the basis set, the presence of D3BJ dispersion correction and the presence of CPCM(CH_2Cl_2) solvent model using the BLYP density functional. In each segment, the bars correspond to the normal modes 81-70 (numeration according to fundamental frequencies in decreasing order) from left to right. (“DZ” = def2-SVP, “TZ” = def2-TZVP)

6.2 Optimized Geometries

DFT-optimized structure of [Mn(ppy)$(CO)_4$] in dependence on the computational model.

BP86/def2-SVP

29
Energy = -2082.5645614 Eh

Mn	0.057701	-1.835428	0.000000
N	1.351330	-0.194811	0.000000
C	2.701927	-0.277059	0.000000
C	3.532733	0.848099	0.000000
C	2.935321	2.121371	0.000000
C	1.541364	2.212335	0.000000
C	0.749553	1.039338	0.000000
C	-0.716657	1.011142	-0.000001
C	-1.514040	2.182392	0.000000
C	-2.909917	2.082513	0.000000
C	-3.512043	0.809294	0.000000
C	-2.721465	-0.354597	0.000000
C	-1.312433	-0.285431	0.000000
H	3.130050	-1.290247	0.000000
H	4.624804	0.718900	0.000000
H	3.552667	3.033258	0.000001
H	1.048147	3.194397	0.000000
H	-1.045500	3.179714	0.000000
H	-3.529494	2.992799	0.000001
H	-4.611047	0.721656	0.000000
H	-3.229151	-1.332634	0.000001
C	1.391343	-3.077923	0.000000
O	2.227247	-3.892131	0.000000
C	-1.225692	-3.092633	0.000000
O	-2.070899	-3.895464	0.000000
C	-0.059172	-1.703255	-1.835096
O	-0.163752	-1.606170	-2.985623
C	-0.059172	-1.703255	1.835095
O	-0.163752	-1.606169	2.985623

BP86-D3BJ/def2-SVP

29
Energy = -2082.6414800 Eh

Mn	0.061821	-1.830142	0.000000
N	1.349027	-0.203142	0.000000
C	2.697929	-0.291012	0.000000
C	3.529527	0.832581	0.000000
C	2.934806	2.106948	0.000000
C	1.541471	2.201552	0.000000
C	0.749476	1.030921	0.000000
C	-0.714012	1.002968	0.000000
C	-1.512441	2.171404	0.000000
C	-2.907509	2.066236	0.000000
C	-3.505968	0.791439	0.000000
C	-2.713157	-0.369987	0.000000
C	-1.306646	-0.292977	0.000000
H	3.121272	-1.305569	0.000000
H	4.621069	0.701899	0.000000
H	3.554282	3.016939	0.000001
H	1.048634	3.183398	0.000000
H	-1.045362	3.168969	0.000000
H	-3.530142	2.973978	0.000001
H	-4.604401	0.701392	0.000000
H	-3.214442	-1.350667	0.000001
C	1.394530	-3.067082	0.000000
O	2.235951	-3.875345	0.000000
C	-1.219666	-3.084699	0.000000
O	-2.069927	-3.881743	0.000000
C	-0.066047	-1.668090	-1.826095
O	-0.182014	-1.531039	-2.971206
C	-0.066047	-1.668090	1.826094
O	-0.182015	-1.531039	2.971206

BP86/def2-SVP CPCM(CH_2Cl_2,PDens=25)

29
Energy = -2082.5713105 Eh

Mn	0.059619	-1.836375	0.000000
N	1.352585	-0.194101	0.000000
C	2.703517	-0.278024	0.000000
C	3.533129	0.848297	0.000000
C	2.936139	2.121307	0.000000
C	1.541118	2.213009	0.000000
C	0.752059	1.039740	0.000000
C	-0.716931	1.010455	0.000000
C	-1.515086	2.181204	0.000000
C	-2.912609	2.080471	0.000000
C	-3.515884	0.807600	0.000000
C	-2.723166	-0.356498	0.000000
C	-1.313973	-0.285343	0.000000
H	3.134431	-1.289432	0.000000
H	4.625015	0.719416	-0.000001
H	3.553198	3.032885	0.000000
H	1.050716	3.196335	0.000000
H	-1.047188	3.178379	0.000000
H	-3.531495	2.991315	0.000000
H	-4.614892	0.719599	0.000000
H	-3.230758	-1.334834	0.000000
C	1.387079	-3.083932	0.000000
O	2.207820	-3.914397	0.000000
C	-1.219069	-3.093524	0.000000
O	-2.061833	-3.901742	0.000000
C	-0.054990	-1.695398	-1.832204
O	-0.161781	-1.590505	-2.982943
C	-0.054990	-1.695398	1.832204
O	-0.161781	-1.590506	2.982943

BP86-D3BJ/def2-SVP
CPCM(CH_2Cl_2,PDens=25)

29
Energy = -2082.6482491 Eh

Mn	0.063449	-1.831776	0.000000
N	1.350772	-0.202828	0.000000
C	2.699925	-0.292137	0.000000
C	3.530218	0.832718	0.000000
C	2.935689	2.106712	0.000000
C	1.541299	2.201833	0.000000
C	0.752192	1.030805	0.000000
C	-0.713965	1.001820	0.000000
C	-1.513068	2.169774	0.000000
C	-2.909770	2.063793	0.000000
C	-3.509477	0.789393	0.000000
C	-2.714627	-0.372291	0.000000
C	-1.308017	-0.293237	0.000000
H	3.126283	-1.304819	0.000000
H	4.621576	0.702481	0.000000
H	3.554689	3.016508	0.000000
H	1.051152	3.184866	0.000000
H	-1.046614	3.167168	0.000000
H	-3.531611	2.972142	0.000001
H	-4.607895	0.699042	0.000000
H	-3.215787	-1.353238	0.000000
C	1.390740	-3.072861	0.000000
O	2.217896	-3.896721	0.000000
C	-1.213304	-3.086241	0.000000
O	-2.060742	-3.889112	0.000000
C	-0.063108	-1.659330	-1.822772
O	-0.182392	-1.512568	-2.967436
C	-0.063109	-1.659329	1.822772
O	-0.182393	-1.512568	2.967435

BP86/def2-TZVP

29
Energy = -2083.7562146 Eh

Mn	0.062935	-1.826977	0.000000
N	1.353097	-0.182692	0.000000
C	2.700530	-0.258181	0.000000
C	3.522151	0.862870	0.000000
C	2.924092	2.127072	0.000000
C	1.538307	2.214149	0.000000
C	0.752214	1.046972	0.000000
C	-0.708458	1.016084	0.000000
C	-1.502119	2.180985	0.000000
C	-2.890151	2.082219	0.000000
C	-3.490111	0.816745	0.000000
C	-2.704075	-0.341191	0.000000
C	-1.303312	-0.271775	0.000000
H	3.126496	-1.260858	0.000000
H	4.604752	0.738527	-0.000001
H	3.533759	3.032266	0.000001
H	1.048452	3.187202	0.000000
H	-1.038242	3.169992	0.000000
H	-3.503827	2.984570	0.000000
H	-4.579412	0.730870	0.000000
H	-3.206893	-1.310875	0.000001
C	1.388040	-3.085371	0.000000
O	2.201460	-3.914630	0.000000
C	-1.205305	-3.102898	0.000000
O	-2.028721	-3.920760	-0.000001
C	-0.079003	-1.716139	-1.837793
O	-0.218827	-1.641018	-2.980877
C	-0.079003	-1.716139	1.837794
O	-0.218826	-1.641018	2.980877

BP86-D3BJ/def2-TZVP

29
Energy = -2083.8331976 Eh

Mn	0.067539	-1.822222	-0.000001
N	1.351115	-0.191987	-0.000001
C	2.696930	-0.273087	0.000000
C	3.519283	0.846496	0.000000
C	2.923883	2.111847	-0.000001
C	1.538658	2.202560	-0.000001
C	0.752290	1.037761	0.000000
C	-0.705585	1.006980	0.000000
C	-1.500613	2.168943	0.000001
C	-2.887879	2.064529	0.000002
C	-3.483957	0.797294	0.000001
C	-2.695397	-0.358036	-0.000001
C	-1.297160	-0.280430	-0.000001
H	3.118198	-1.277084	-0.000002
H	4.601395	0.720886	0.000004
H	3.535708	3.015136	-0.000002
H	1.049275	3.175467	-0.000001
H	-1.038511	3.158346	0.000001
H	-3.504824	2.964199	0.000001
H	-4.572675	0.708886	0.000004
H	-3.191667	-1.330444	-0.000003
C	1.393202	-3.073951	0.000000
O	2.212976	-3.896667	0.000002
C	-1.200870	-3.093675	-0.000001
O	-2.031579	-3.903675	0.000000
C	-0.086006	-1.678708	-1.828633
O	-0.238857	-1.560330	-2.966151
C	-0.086008	-1.678707	1.828632
O	-0.238859	-1.560328	2.966150

BP86/def2-TZVP CPCM(CH_2Cl_2,PDens=25)

29
Energy = -2083.7633989 Eh

Mn	0.064694	-1.828004	0.000000
N	1.354824	-0.182219	0.000000
C	2.702505	-0.259606	0.000000
C	3.523032	0.862645	0.000000
C	2.925485	2.126630	0.000000
C	1.538546	2.214505	0.000000
C	0.755225	1.047139	0.000000
C	-0.708300	1.015339	0.000000
C	-1.502921	2.179612	0.000000
C	-2.892706	2.079900	0.000000
C	-3.493865	0.814674	0.000000
C	-2.705466	-0.343483	0.000000
C	-1.304600	-0.271462	0.000000
H	3.130991	-1.260563	0.000000
H	4.605392	0.738424	0.000000
H	3.534857	3.031469	0.000000
H	1.051494	3.188770	0.000000
H	-1.039778	3.168474	0.000000
H	-3.505671	2.982816	0.000000
H	-4.583129	0.728290	0.000000
H	-3.207923	-1.313548	0.000000
C	1.383122	-3.091389	0.000000
O	2.180632	-3.937005	0.000000
C	-1.196353	-3.105853	0.000000
O	-2.014023	-3.932327	0.000000
C	-0.077133	-1.705977	-1.834331
O	-0.220900	-1.620639	-2.977337
C	-0.077133	-1.705977	1.834331
O	-0.220900	-1.620639	2.977337

BP86-D3BJ/def2-TZVP CPCM(CH_2Cl_2,PDens=25)

29
Energy = -2083.8404139 Eh

Mn	0.069399	-1.823386	-0.000001
N	1.352839	-0.191382	-0.000001
C	2.698775	-0.274405	0.000000
C	3.519991	0.846407	0.000001
C	2.925049	2.111412	0.000000
C	1.538598	2.202890	-0.000001
C	0.755246	1.037845	-0.000001
C	-0.705520	1.006000	-0.000001
C	-1.501388	2.167297	0.000000
C	-2.890360	2.061896	0.000002
C	-3.487640	0.795043	0.000001
C	-2.696753	-0.360526	0.000000
C	-1.298640	-0.280193	-0.000001
H	3.122617	-1.276668	-0.000001
H	4.601856	0.720967	0.000004
H	3.536499	3.014370	-0.000001
H	1.052245	3.177060	-0.000002
H	-1.040125	3.156562	0.000000
H	-3.506465	2.962196	0.000002
H	-4.576270	0.706116	0.000004
H	-3.192701	-1.333244	-0.000001
C	1.387696	-3.080609	0.000000
O	2.191093	-3.920444	0.000001
C	-1.192241	-3.095882	0.000000
O	-2.017350	-3.914681	0.000000
C	-0.083657	-1.668212	-1.824854
O	-0.239567	-1.539110	-2.962017
C	-0.083659	-1.668211	1.824853
O	-0.239569	-1.539107	2.962015

PBE/def2-SVP

29
Energy = -2081.0808439 Eh

Mn	0.059424	-1.832151	0.000000
N	1.351493	-0.194313	0.000000
C	2.699852	-0.277467	0.000000
C	3.529941	0.846084	0.000000
C	2.933698	2.117828	0.000000
C	1.541522	2.209128	0.000000
C	0.750768	1.037653	0.000000
C	-0.713417	1.009296	-0.000001
C	-1.511301	2.177838	0.000000
C	-2.905241	2.076134	0.000000
C	-3.505153	0.803913	0.000000
C	-2.714508	-0.357538	0.000000
C	-1.307509	-0.285865	0.000000
H	3.127387	-1.290366	0.000000
H	4.621240	0.716598	0.000000
H	3.550937	3.028915	0.000001
H	1.048706	3.190591	0.000000
H	-1.044443	3.175095	0.000000
H	-3.525563	2.984937	0.000001
H	-4.603272	0.715190	0.000000
H	-3.220123	-1.335923	0.000001
C	1.390989	-3.071273	0.000000
O	2.228998	-3.882115	0.000000
C	-1.221207	-3.086247	0.000000
O	-2.068759	-3.885477	0.000000
C	-0.066139	-1.697589	-1.830424
O	-0.181093	-1.597643	-2.978932
C	-0.066139	-1.697589	1.830424
O	-0.181092	-1.597642	2.978932

PBE-D3BJ/def2-SVP

29
Energy = -2081.1266277 Eh

Mn	0.062089	-1.829392	0.000000
N	1.350277	-0.199438	0.000000
C	2.697669	-0.285704	0.000000
C	3.528111	0.836999	0.000000
C	2.933403	2.109320	0.000000
C	1.541617	2.202619	0.000000
C	0.750882	1.032489	0.000000
C	-0.711692	1.004274	0.000000
C	-1.510112	2.171172	0.000000
C	-2.903553	2.066434	0.000000
C	-3.501323	0.793343	0.000000
C	-2.709413	-0.366686	0.000000
C	-1.303907	-0.290514	0.000000
H	3.122337	-1.299401	0.000000
H	4.619052	0.706688	0.000000
H	3.551795	3.019293	0.000001
H	1.048720	3.183746	0.000000
H	-1.043859	3.168394	0.000000
H	-3.525604	2.973721	0.000001
H	-4.599051	0.703212	0.000000
H	-3.211131	-1.346653	0.000001
C	1.393487	-3.064711	0.000000
O	2.235501	-3.871234	0.000000
C	-1.217492	-3.081726	0.000000
O	-2.068484	-3.877018	0.000000
C	-0.070901	-1.676766	-1.824907
O	-0.193758	-1.552848	-2.970106
C	-0.070901	-1.676765	1.824907
O	-0.193758	-1.552847	2.970106

PBE/def2-SVP CPCM(CH_2Cl_2,PDens=25)

29
Energy = -2081.0874494 Eh

Mn	0.061279	-1.833193	0.000000
N	1.352766	-0.193682	0.000000
C	2.701431	-0.278478	0.000000
C	3.530333	0.846225	0.000000
C	2.934469	2.117703	0.000000
C	1.541261	2.209733	0.000000
C	0.753233	1.037960	0.000000
C	-0.713671	1.008508	0.000000
C	-1.512332	2.176546	0.000000
C	-2.907878	2.073963	0.000000
C	-3.508923	0.802072	0.000000
C	-2.716164	-0.359570	0.000000
C	-1.309005	-0.285897	0.000000
H	3.131770	-1.289622	0.000000
H	4.621489	0.717086	0.000000
H	3.551438	3.028524	0.000000
H	1.051212	3.192479	0.000000
H	-1.046042	3.173666	0.000000
H	-3.527538	2.983330	0.000000
H	-4.607059	0.713001	0.000000
H	-3.221653	-1.338267	0.000000
C	1.386588	-3.077456	0.000000
O	2.209489	-3.904581	0.000000
C	-1.214637	-3.087313	0.000000
O	-2.059750	-3.891894	0.000000
C	-0.062028	-1.689383	-1.827535
O	-0.179024	-1.581039	-2.976151
C	-0.062028	-1.689383	1.827535
O	-0.179025	-1.581039	2.976151

PBE-D3BJ/def2-SVP CPCM(CH_2Cl_2,PDens=25)

29
Energy = -2081.1332444 Eh

Mn	0.063692	-1.831033	0.000000
N	1.351969	-0.199160	0.000000
C	2.699602	-0.286862	0.000000
C	3.528754	0.837096	0.000000
C	2.934232	2.109046	0.000000
C	1.541417	2.202876	0.000000
C	0.753549	1.032337	0.000000
C	-0.711659	1.003089	0.000000
C	-1.510751	2.169513	0.000000
C	-2.905792	2.063969	0.000000
C	-3.504804	0.791269	0.000000
C	-2.710886	-0.369019	0.000000
C	-1.305268	-0.290836	0.000000
H	3.127299	-1.298704	0.000000
H	4.619556	0.707235	0.000000
H	3.552182	3.018863	0.000001
H	1.051178	3.185228	0.000000
H	-1.045029	3.166574	0.000000
H	-3.527065	2.971887	0.000001
H	-4.602542	0.700857	0.000000
H	-3.212482	-1.349263	0.000000
C	1.389655	-3.070545	0.000000
O	2.217569	-3.892503	0.000000
C	-1.211156	-3.083368	0.000000
O	-2.059461	-3.884300	0.000000
C	-0.067974	-1.667932	-1.821701
O	-0.193905	-1.534192	-2.966531
C	-0.067975	-1.667931	1.821700
O	-0.193906	-1.534192	2.966530

PBE/def2-TZVP

29

Energy = -2082.2642422 Eh

Mn	0.065771	-1.823115	0.000000
N	1.353895	-0.182199	0.000000
C	2.699422	-0.258476	0.000000
C	3.520680	0.861334	0.000000
C	2.923634	2.124446	0.000000
C	1.539129	2.211730	0.000000
C	0.754105	1.045620	0.000000
C	-0.704854	1.014438	0.000000
C	-1.499284	2.176867	0.000000
C	-2.885829	2.075948	0.000000
C	-3.483476	0.810918	0.000000
C	-2.696864	-0.344827	0.000000
C	-1.297827	-0.272371	0.000000
H	3.125251	-1.261069	0.000000
H	4.602905	0.736500	-0.000001
H	3.533352	3.029242	0.000001
H	1.049469	3.184544	0.000000
H	-1.037157	3.166343	0.000000
H	-3.500688	2.977085	0.000000
H	-4.572320	0.723598	0.000000
H	-3.197679	-1.315341	0.000001
C	1.387696	-3.079385	0.000000
O	2.201048	-3.908138	0.000000
C	-1.198509	-3.096889	0.000000
O	-2.023179	-3.913014	-0.000001
C	-0.087753	-1.709858	-1.832533
O	-0.241593	-1.632036	-2.973332
C	-0.087753	-1.709858	1.832533
O	-0.241592	-1.632036	2.973332

PBE-D3BJ/def2-TZVP

29

Energy = -2083.3100632 Eh

Mn	0.068824	-1.820701	0.000002
N	1.352758	-0.187602	0.000004
C	2.697345	-0.267024	-0.000001
C	3.518935	0.852001	-0.000002
C	2.923365	2.115701	-0.000003
C	1.539210	2.204958	0.000001
C	0.754169	1.040169	0.000003
C	-0.703157	1.008930	0.000003
C	-1.498356	2.169616	0.000001
C	-2.884411	2.065407	-0.000002
C	-3.479713	0.799371	-0.000001
C	-2.691622	-0.354832	0.000000
C	-1.294103	-0.277656	0.000003
H	3.120347	-1.270543	-0.000002
H	4.600945	0.726454	-0.000004
H	3.534293	3.019498	-0.000007
H	1.049467	3.177583	0.000003
H	-1.037024	3.159283	0.000003
H	-3.501231	2.965017	-0.000004
H	-4.568274	0.710477	-0.000001
H	-3.188476	-1.327099	-0.000003
C	1.390387	-3.073612	0.000002
O	2.207991	-3.898013	0.000000
C	-1.195977	-3.091226	0.000000
O	-2.026550	-3.901061	-0.000006
C	-0.091570	-1.688308	-1.827034
O	-0.252997	-1.584237	-2.964546
C	-0.091573	-1.688311	1.827038
O	-0.253002	-1.584240	2.964550

PBE/def2-TZVP
CPCM(CH_2Cl_2,PDens=25)

29

Energy = -2082.2713720 Eh

Mn	0.067846	-1.824465	0.000000
N	1.356099	-0.181968	0.000000
C	2.701823	-0.260027	0.000000
C	3.521896	0.861045	0.000000
C	2.925212	2.123886	-0.000001
C	1.539541	2.211835	0.000000
C	0.757445	1.045421	0.000000
C	-0.704349	1.013257	0.000000
C	-1.499755	2.175031	0.000001
C	-2.888008	2.073169	0.000001
C	-3.486907	0.808433	0.000000
C	-2.697955	-0.347510	0.000000
C	-1.298942	-0.272337	0.000000
H	3.130332	-1.260865	0.000000
H	4.603908	0.736407	0.000000
H	3.534547	3.028407	-0.000001
H	1.052681	3.185869	0.000000
H	-1.038373	3.164379	0.000001
H	-3.502095	2.974913	0.000001
H	-4.575700	0.720606	0.000001
H	-3.198349	-1.318433	-0.000001
C	1.383480	-3.085222	0.000001
O	2.181204	-3.930060	0.000001
C	-1.188860	-3.100432	-0.000001
O	-2.007606	-3.925366	-0.000001
C	-0.087444	-1.698705	-1.828762
O	-0.247111	-1.609283	-2.969065
C	-0.087445	-1.698704	1.828762
O	-0.247114	-1.609282	2.969064

PBE-D3BJ/def2-TZVP
CPCM(CH_2Cl_2,PDens=25)

29

Energy = -2082.3172126 Eh

Mn	0.070659	-1.822001	-0.000001
N	1.354753	-0.187284	-0.000001
C	2.699461	-0.268670	0.000000
C	3.519957	0.851575	0.000001
C	2.924769	2.114996	0.000000
C	1.539459	2.205077	-0.000001
C	0.757254	1.040022	-0.000001
C	-0.702813	1.007863	-0.000001
C	-1.498936	2.167871	0.000000
C	-2.886692	2.062633	0.000001
C	-3.483200	0.796903	0.000001
C	-2.692799	-0.357519	0.000000
C	-1.295315	-0.277536	-0.000001
H	3.124998	-1.270348	0.000000
H	4.601629	0.726239	0.000002
H	3.535275	3.018400	0.000001
H	1.052686	3.178867	-0.000001
H	-1.038400	3.157301	0.000000
H	-3.502676	2.962746	0.000002
H	-4.571589	0.707510	0.000002
H	-3.189242	-1.330020	0.000000
C	1.385802	-3.079512	0.000000
O	2.187556	-3.920406	0.000000
C	-1.187126	-3.094198	0.000000
O	-2.011040	-3.913718	0.000000
C	-0.090469	-1.676576	-1.823232
O	-0.256745	-1.561821	-2.960183
C	-0.090470	-1.676575	1.823231
O	-0.256746	-1.561819	2.960182

BLYP/def2-SVP

29

Energy = -2082.1606595 Eh

Mn	0.049818	-1.860273	0.000000
N	1.361185	-0.188954	0.000000
C	2.715227	-0.264919	0.000000
C	3.542458	0.865267	0.000000
C	2.937576	2.137254	0.000000
C	1.541316	2.222738	0.000000
C	0.750463	1.045137	0.000000
C	-0.721324	1.015366	0.000000
C	-1.517343	2.191582	0.000000
C	-2.915696	2.098595	0.000000
C	-3.525538	0.827016	0.000000
C	-2.738214	-0.341621	0.000000
C	-1.326745	-0.280009	0.000000
H	3.148834	-1.273625	0.000000
H	4.633809	0.742124	-0.000001
H	3.549782	3.050892	0.000000
H	1.048772	3.202766	0.000000
H	-1.047914	3.186265	0.000000
H	-3.529505	3.011157	0.000000
H	-4.623590	0.744832	-0.000001
H	-3.250995	-1.315011	0.000001
C	1.403251	-3.114647	0.000000
O	2.247549	-3.920145	0.000000
C	-1.255757	-3.124637	0.000000
O	-2.109782	-3.918296	0.000000
C	-0.048834	-1.731805	-1.859333
O	-0.134984	-1.637622	-3.011828
C	-0.048834	-1.731806	1.859333
O	-0.134984	-1.637622	3.011828

BLYP-D3BJ/def2-SVP

29

Energy = -2082.2559312 Eh

Mn	0.054905	-1.852071	0.000000
N	1.357070	-0.199744	0.000000
C	2.708851	-0.282700	0.000000
C	3.537225	0.845441	0.000000
C	2.935896	2.118717	0.000000
C	1.540515	2.208703	0.000000
C	0.749535	1.034186	0.000000
C	-0.718382	1.004879	0.000000
C	-1.515355	2.177603	0.000000
C	-2.912627	2.078281	0.000000
C	-3.517781	0.804829	0.000000
C	-2.727631	-0.360731	0.000000
C	-1.319456	-0.289587	0.000000
H	3.136372	-1.293080	0.000000
H	4.627839	0.720408	-0.000001
H	3.550808	3.029874	0.000000
H	1.048182	3.188238	0.000000
H	-1.047282	3.172287	0.000000
H	-3.530181	2.987646	0.000000
H	-4.615057	0.719661	0.000000
H	-3.232468	-1.337325	0.000000
C	1.406984	-3.097953	0.000000
O	2.259297	-3.894699	0.000000
C	-1.249831	-3.110092	0.000000
O	-2.112390	-3.893905	0.000000
C	-0.054708	-1.689899	-1.847555
O	-0.152812	-1.549533	-2.994077
C	-0.054708	-1.689899	1.847555
O	-0.152813	-1.549533	2.994077

BLYP/def2-SVP
CPCM(CH_2Cl_2,PDens=25)
29
Energy = -2082.1669059 Eh

Mn	0.051646	-1.861022	0.000000
N	1.362044	-0.188409	0.000000
C	2.716368	-0.265979	0.000000
C	3.542453	0.865329	0.000000
C	2.938103	2.137034	0.000000
C	1.540802	2.223074	0.000000
C	0.752718	1.045364	0.000000
C	-0.721861	1.014678	0.000000
C	-1.518452	2.190311	0.000000
C	-2.918444	2.096738	0.000000
C	-3.529407	0.825572	0.000000
C	-2.740053	-0.343289	0.000000
C	-1.328429	-0.279851	0.000000
H	3.152249	-1.273107	0.000000
H	4.633527	0.742363	-0.000001
H	3.549972	3.050310	0.000000
H	1.050552	3.204062	0.000000
H	-1.049330	3.184621	0.000000
H	-3.531476	3.009824	0.000000
H	-4.627413	0.743028	-0.000001
H	-3.252552	-1.317058	0.000001
C	1.399289	-3.119913	0.000000
O	2.229101	-3.941284	0.000000
C	-1.249532	-3.124399	0.000000
O	-2.102084	-3.922586	0.000000
C	-0.043825	-1.724622	-1.856615
O	-0.131070	-1.623084	-3.009370
C	-0.043825	-1.724622	1.856615
O	-0.131070	-1.623085	3.009370

BLYP-D3BJ/def2-SVP
CPCM(CH_2Cl_2,PDens=25)
29
Energy = -2082.2622046 Eh

Mn	0.056863	-1.853055	0.000000
N	1.358311	-0.199219	0.000000
C	2.710324	-0.283708	0.000000
C	3.537529	0.845547	0.000000
C	2.936588	2.118503	0.000000
C	1.540193	2.208991	0.000000
C	0.752011	1.034288	0.000000
C	-0.718589	1.004003	0.000000
C	-1.516191	2.176090	0.000000
C	-2.915068	2.076039	0.000000
C	-3.521285	0.802972	0.000000
C	-2.729050	-0.362744	0.000000
C	-1.320816	-0.289560	0.000000
H	3.140150	-1.292520	0.000000
H	4.627875	0.720785	-0.000001
H	3.551104	3.029342	0.000000
H	1.050101	3.189453	0.000000
H	-1.048492	3.170427	0.000000
H	-3.531913	2.985887	0.000000
H	-4.618505	0.717388	0.000000
H	-3.233436	-1.339783	0.000000
C	1.403348	-3.103171	0.000000
O	2.240985	-3.916366	0.000000
C	-1.243231	-3.110359	0.000000
O	-2.103916	-3.899243	0.000000
C	-0.050930	-1.681819	-1.844448
O	-0.151515	-1.533174	-2.990717
C	-0.050930	-1.681819	1.844449
O	-0.151515	-1.533174	2.990717

BLYP/def2-TZVP

29
Energy = -2083.3748187 Eh

Mn	0.053352	-1.854084	0.000000
N	1.363300	-0.176915	0.000000
C	2.714121	-0.246304	0.000000
C	3.531795	0.879617	0.000000
C	2.926096	2.142216	0.000000
C	1.538296	2.223865	0.000000
C	0.752957	1.052199	0.000000
C	-0.713458	1.019862	0.000000
C	-1.505922	2.189807	0.000000
C	-2.896227	2.098232	0.000000
C	-3.504083	0.834754	0.000000
C	-2.721389	-0.327812	0.000000
C	-1.318353	-0.266211	0.000000
H	3.145586	-1.243220	0.000000
H	4.612572	0.761875	0.000000
H	3.529836	3.048247	0.000000
H	1.050147	3.194196	0.000000
H	-1.041762	3.175172	0.000000
H	-3.503169	3.002080	0.000000
H	-4.591192	0.754282	0.000000
H	-3.229105	-1.291623	0.000000
C	1.398481	-3.124589	0.000000
O	2.220025	-3.945554	0.000000
C	-1.235761	-3.138971	0.000000
O	-2.065475	-3.950433	0.000000
C	-0.068598	-1.741747	-1.862044
O	-0.186736	-1.663598	-3.007344
C	-0.068598	-1.741747	1.862044
O	-0.186735	-1.663597	3.007344

BLYP-D3BJ/def2-TZVP

29
Energy = -2083.4701899 Eh

Mn	0.059781	-1.846104	0.000000
N	1.359597	-0.188746	0.000000
C	2.708175	-0.265230	0.000000
C	3.527085	0.858666	0.000000
C	2.925036	2.122704	0.000000
C	1.538024	2.208936	0.000000
C	0.752404	1.040418	0.000000
C	-0.710045	1.008406	0.000000
C	-1.504010	2.174554	0.000000
C	-2.893273	2.075994	0.000000
C	-3.495985	0.810306	0.000000
C	-2.709952	-0.348887	0.000000
C	-1.310272	-0.277042	0.000000
H	3.133565	-1.263847	0.000000
H	4.607136	0.739027	0.000000
H	3.531568	3.026200	0.000000
H	1.050217	3.178867	0.000000
H	-1.041722	3.160179	0.000000
H	-3.504358	2.976375	0.000000
H	-4.582283	0.726472	0.000000
H	-3.209363	-1.316133	0.000000
C	1.404559	-3.107399	0.000000
O	2.234896	-3.919173	0.000001
C	-1.230682	-3.122476	0.000000
O	-2.071305	-3.922054	0.000000
C	-0.075582	-1.696584	-1.849710
O	-0.208814	-1.568422	-2.988578
C	-0.075582	-1.696584	1.849710
O	-0.208815	-1.568421	2.988578

BLYP/def2-TZVP
CPCM(CH_2Cl_2,PDens=25)
29
Energy = -2083.3819207 Eh

Mn	0.055625	-1.854278	0.000000
N	1.364384	-0.176261	0.000000
C	2.715445	-0.247551	0.000000
C	3.532072	0.879548	0.000000
C	2.927093	2.141939	0.000000
C	1.538154	2.224323	0.000000
C	0.755633	1.052634	0.000000
C	-0.713652	1.019499	0.000000
C	-1.506936	2.188695	0.000000
C	-2.898980	2.096206	0.000000
C	-3.507840	0.832930	0.000000
C	-2.722805	-0.329825	0.000000
C	-1.319632	-0.265594	0.000000
H	3.148989	-1.242875	0.000000
H	4.612555	0.761847	0.000000
H	3.530588	3.047543	0.000001
H	1.052391	3.195641	-0.000001
H	-1.043274	3.173784	0.000000
H	-3.505278	3.000523	0.000001
H	-4.594896	0.751904	0.000000
H	-3.229997	-1.294086	0.000000
C	1.393201	-3.130427	0.000000
O	2.197619	-3.969324	0.000000
C	-1.226713	-3.139766	0.000000
O	-2.051675	-3.959222	0.000000
C	-0.065189	-1.732830	-1.858571
O	-0.185847	-1.646074	-3.004122
C	-0.065189	-1.732830	1.858571
O	-0.185847	-1.646073	3.004121

BLYP-D3BJ/def2-TZVP
CPCM(CH_2Cl_2,PDens=25)
29
Energy = -2083.4773325 Eh

Mn	0.062135	-1.846374	0.000000
N	1.361048	-0.187964	0.000000
C	2.709806	-0.266329	0.000000
C	3.527682	0.858678	0.000000
C	2.926237	2.122482	0.000000
C	1.538117	2.209447	0.000000
C	0.755310	1.040863	0.000000
C	-0.709909	1.007974	0.000000
C	-1.504709	2.173338	0.000000
C	-2.895676	2.073786	0.000000
C	-3.499388	0.808318	0.000000
C	-2.710989	-0.351004	0.000000
C	-1.311320	-0.276384	0.000000
H	3.137225	-1.263416	0.000000
H	4.607438	0.739122	0.000000
H	3.532487	3.025572	0.000000
H	1.052712	3.180354	-0.000001
H	-1.042977	3.158695	0.000000
H	-3.506136	2.974622	0.000001
H	-4.585618	0.723904	0.000001
H	-3.209748	-1.318748	0.000000
C	1.399826	-3.112866	0.000000
O	2.213184	-3.942859	0.000000
C	-1.221148	-3.123945	0.000000
O	-2.056406	-3.932346	0.000000
C	-0.073469	-1.687038	-1.845898
O	-0.211122	-1.550423	-2.984445
C	-0.073470	-1.687038	1.845898
O	-0.211123	-1.550422	2.984445

BP86/def2-SVP
CPCM(CH_2Cl_2,PDens=5)
29
Energy = -2082.5713105 Eh

Mn	0.059619	-1.836375	0.000000
N	1.352585	-0.194101	0.000000
C	2.703517	-0.278024	0.000000
C	3.533129	0.848297	0.000000
C	2.936139	2.121307	0.000000
C	1.541118	2.213009	0.000000
C	0.752059	1.039740	0.000000
C	-0.716931	1.010455	0.000000
C	-1.515086	2.181204	0.000000
C	-2.912609	2.080471	0.000000
C	-3.515884	0.807600	0.000000
C	-2.723166	-0.356498	0.000000
C	-1.313973	-0.285343	0.000000
H	3.134431	-1.289432	0.000000
H	4.625015	0.719416	-0.000001
H	3.553198	3.032885	0.000000
H	1.050716	3.196335	0.000000
H	-1.047188	3.178379	0.000000
H	-3.531495	2.991315	0.000000
H	-4.614892	0.719599	0.000000
H	-3.230758	-1.334834	0.000000
C	1.387079	-3.083932	0.000000
O	2.207820	-3.914397	0.000000
C	-1.219069	-3.093524	0.000000
O	-2.061833	-3.901742	0.000000
C	-0.054990	-1.695398	-1.832204
O	-0.161781	-1.590505	-2.982943
C	-0.054990	-1.695398	1.832204
O	-0.161781	-1.590506	2.982943

BP86/def2-SVP
CPCM(CH_2Cl_2,PDens=10)
29
Energy = -2082.5713105 Eh

Mn	0.059619	-1.836375	0.000000
N	1.352585	-0.194101	0.000000
C	2.703517	-0.278024	0.000000
C	3.533129	0.848297	0.000000
C	2.936139	2.121307	0.000000
C	1.541118	2.213009	0.000000
C	0.752059	1.039740	0.000000
C	-0.716931	1.010455	0.000000
C	-1.515086	2.181204	0.000000
C	-2.912609	2.080471	0.000000
C	-3.515884	0.807600	0.000000
C	-2.723166	-0.356498	0.000000
C	-1.313973	-0.285343	0.000000
H	3.134431	-1.289432	0.000000
H	4.625015	0.719416	-0.000001
H	3.553198	3.032885	0.000000
H	1.050716	3.196335	0.000000
H	-1.047188	3.178379	0.000000
H	-3.531495	2.991315	0.000000
H	-4.614892	0.719599	0.000000
H	-3.230758	-1.334834	0.000000
C	1.387079	-3.083932	0.000000
O	2.207820	-3.914397	0.000000
C	-1.219069	-3.093524	0.000000
O	-2.061833	-3.901742	0.000000
C	-0.054990	-1.695398	-1.832204
O	-0.161781	-1.590505	-2.982943
C	-0.054990	-1.695398	1.832204
O	-0.161781	-1.590506	2.982943

BP86/def2-SVP
CPCM(CH_2Cl_2,PDens=15)
29
Energy = -2082.5713105 Eh

Mn	0.059619	-1.836375	0.000000
N	1.352585	-0.194101	0.000000
C	2.703517	-0.278024	0.000000
C	3.533129	0.848297	0.000000
C	2.936139	2.121307	0.000000
C	1.541118	2.213009	0.000000
C	0.752059	1.039740	0.000000
C	-0.716931	1.010455	0.000000
C	-1.515086	2.181204	0.000000
C	-2.912609	2.080471	0.000000
C	-3.515884	0.807600	0.000000
C	-2.723166	-0.356498	0.000000
C	-1.313973	-0.285343	0.000000
H	3.134431	-1.289432	0.000000
H	4.625015	0.719416	-0.000001
H	3.553198	3.032885	0.000000
H	1.050716	3.196335	0.000000
H	-1.047188	3.178379	0.000000
H	-3.531495	2.991315	0.000000
H	-4.614892	0.719599	0.000000
H	-3.230758	-1.334834	0.000000
C	1.387079	-3.083932	0.000000
O	2.207820	-3.914397	0.000000
C	-1.219069	-3.093524	0.000000
O	-2.061833	-3.901742	0.000000
C	-0.054990	-1.695398	-1.832204
O	-0.161781	-1.590505	-2.982943
C	-0.054990	-1.695398	1.832204
O	-0.161781	-1.590506	2.982943

BP86/def2-SVP
CPCM(CH_2Cl_2,PDens=20)
29
Energy = -2082.5713105 Eh

Mn	0.059619	-1.836375	0.000000
N	1.352585	-0.194101	0.000000
C	2.703517	-0.278024	0.000000
C	3.533129	0.848297	0.000000
C	2.936139	2.121307	0.000000
C	1.541118	2.213009	0.000000
C	0.752059	1.039740	0.000000
C	-0.716931	1.010455	0.000000
C	-1.515086	2.181204	0.000000
C	-2.912609	2.080471	0.000000
C	-3.515884	0.807600	0.000000
C	-2.723166	-0.356498	0.000000
C	-1.313973	-0.285343	0.000000
H	3.134431	-1.289432	0.000000
H	4.625015	0.719416	-0.000001
H	3.553198	3.032885	0.000000
H	1.050716	3.196335	0.000000
H	-1.047188	3.178379	0.000000
H	-3.531495	2.991315	0.000000
H	-4.614892	0.719599	0.000000
H	-3.230758	-1.334834	0.000000
C	1.387079	-3.083932	0.000000
O	2.207820	-3.914397	0.000000
C	-1.219069	-3.093524	0.000000
O	-2.061833	-3.901742	0.000000
C	-0.054990	-1.695398	-1.832204
O	-0.161781	-1.590505	-2.982943
C	-0.054990	-1.695398	1.832204
O	-0.161781	-1.590506	2.982943

BP86/def2-SVP
CPCM(CH_2Cl_2,PDens=30)
29
Energy = -2082.5713105 Eh

Mn	0.059619	-1.836375	0.000000
N	1.352585	-0.194101	0.000000
C	2.703517	-0.278024	0.000000
C	3.533129	0.848297	0.000000
C	2.936139	2.121307	0.000000
C	1.541118	2.213009	0.000000
C	0.752059	1.039740	0.000000
C	-0.716931	1.010455	0.000000
C	-1.515086	2.181204	0.000000
C	-2.912609	2.080471	0.000000
C	-3.515884	0.807600	0.000000
C	-2.723166	-0.356498	0.000000
C	-1.313973	-0.285343	0.000000
H	3.134431	-1.289432	0.000000
H	4.625015	0.719416	-0.000001
H	3.553198	3.032885	0.000000
H	1.050716	3.196335	0.000000
H	-1.047188	3.178379	0.000000
H	-3.531495	2.991315	0.000000
H	-4.614892	0.719599	0.000000
H	-3.230758	-1.334834	0.000000
C	1.387079	-3.083932	0.000000
O	2.207820	-3.914397	0.000000
C	-1.219069	-3.093524	0.000000
O	-2.061833	-3.901742	0.000000
C	-0.054990	-1.695398	-1.832204
O	-0.161781	-1.590505	-2.982943
C	-0.054990	-1.695398	1.832204
O	-0.161781	-1.590506	2.982943

BP86/def2-SVP
CPCM(CH_2Cl_2,PDens=40)
29
Energy = -2082.5713105 Eh

Mn	0.059619	-1.836375	0.000000
N	1.352585	-0.194101	0.000000
C	2.703517	-0.278024	0.000000
C	3.533129	0.848297	0.000000
C	2.936139	2.121307	0.000000
C	1.541118	2.213009	0.000000
C	0.752059	1.039740	0.000000
C	-0.716931	1.010455	0.000000
C	-1.515086	2.181204	0.000000
C	-2.912609	2.080471	0.000000
C	-3.515884	0.807600	0.000000
C	-2.723166	-0.356498	0.000000
C	-1.313973	-0.285343	0.000000
H	3.134431	-1.289432	0.000000
H	4.625015	0.719416	-0.000001
H	3.553198	3.032885	0.000000
H	1.050716	3.196335	0.000000
H	-1.047188	3.178379	0.000000
H	-3.531495	2.991315	0.000000
H	-4.614892	0.719599	0.000000
H	-3.230758	-1.334834	0.000000
C	1.387079	-3.083932	0.000000
O	2.207820	-3.914397	0.000000
C	-1.219069	-3.093524	0.000000
O	-2.061833	-3.901742	0.000000
C	-0.054990	-1.695398	-1.832204
O	-0.161781	-1.590505	-2.982943
C	-0.054990	-1.695398	1.832204
O	-0.161781	-1.590506	2.982943

BP86/def2-SVP
CPCM(CH_2Cl_2,PDens=50)
29
Energy -2082.5713105 Eh

Mn	0.059619	-1.836375	0.000000
N	1.352585	-0.194101	0.000000
C	2.703517	-0.278024	0.000000
C	3.533129	0.848297	0.000000
C	2.936139	2.121307	0.000000
C	1.541118	2.213009	0.000000
C	0.752059	1.039740	0.000000
C	-0.716931	1.010455	0.000000
C	-1.515086	2.181204	0.000000
C	-2.912609	2.080471	0.000000
C	-3.515884	0.807600	0.000000
C	-2.723166	-0.356498	0.000000
C	-1.313973	-0.285343	0.000000
H	3.134431	-1.289432	0.000000
H	4.625015	0.719416	-0.000001
H	3.553198	3.032885	0.000000
H	1.050716	3.196335	0.000000
H	-1.047188	3.178379	0.000000
H	-3.531495	2.991315	0.000000
H	-4.614892	0.719599	0.000000
H	-3.230758	-1.334834	0.000000
C	1.387079	-3.083932	0.000000
O	2.207820	-3.914397	0.000000
C	-1.219069	-3.093524	0.000000
O	-2.061833	-3.901742	0.000000
C	-0.054990	-1.695398	-1.832204
O	-0.161781	-1.590505	-2.982943
C	-0.054990	-1.695398	1.832204
O	-0.161781	-1.590506	2.982943

BP86/def2-SVP
CPCM(*n*-heptane,PDens=25)
29
Energy = -2082.5679214 Eh

Mn	0.058399	-1.836127	0.000000
N	1.352024	-0.194569	0.000000
C	2.702756	-0.277605	0.000000
C	3.533005	0.848145	0.000000
C	2.935726	2.121306	0.000000
C	1.541238	2.212574	0.000000
C	0.750785	1.039372	-0.000001
C	-0.716758	1.010716	-0.000001
C	-1.514419	2.181806	0.000000
C	-2.911124	2.081548	0.000000
C	-3.513855	0.808481	0.000000
C	-2.722314	-0.355578	0.000000
C	-1.313204	-0.285438	0.000000
H	3.132243	-1.289920	0.000000
H	4.624986	0.719060	0.000000
H	3.552872	3.033091	0.000001
H	1.049307	3.195213	-0.000001
H	-1.046177	3.179045	0.000000
H	-3.530356	2.992100	0.000001
H	-4.612835	0.720654	0.000000
H	-3.230018	-1.333687	0.000001
C	1.388998	-3.080856	0.000000
O	2.218058	-3.902573	0.000000
C	-1.222599	-3.093561	0.000000
O	-2.066088	-3.899434	0.000000
C	-0.057494	-1.699251	-1.833625
O	-0.162831	-1.597630	-2.984204
C	-0.057494	-1.699251	1.833624
O	-0.162831	-1.597629	2.984204

BP86/def2-SVP
CPCM(toluene,PDens=25)
29
Energy = -2082.5687128 Eh

Mn	0.059015	-1.835772	0.000000
N	1.352008	-0.194226	0.000000
C	2.702826	-0.277538	0.000000
C	3.532981	0.848263	0.000000
C	2.935850	2.121434	0.000000
C	1.541258	2.212909	0.000000
C	0.751059	1.039732	0.000000
C	-0.716827	1.010888	0.000000
C	-1.514675	2.181859	0.000000
C	-2.911546	2.081383	0.000000
C	-3.514291	0.808327	0.000000
C	-2.722410	-0.355665	0.000000
C	-1.313297	-0.285242	0.000000
H	3.132575	-1.289675	0.000000
H	4.624942	0.719188	0.000000
H	3.553067	3.033119	0.000000
H	1.049725	3.195723	0.000000
H	-1.046567	3.179107	0.000000
H	-3.530820	2.991931	0.000000
H	-4.613277	0.720394	0.000000
H	-3.230066	-1.333848	0.000000
C	1.388519	-3.081540	0.000000
O	2.215200	-3.905816	0.000000
C	-1.221520	-3.093151	0.000000
O	-2.064998	-3.899432	0.000000
C	-0.056737	-1.698738	-1.833340
O	-0.162628	-1.597438	-2.984083
C	-0.056737	-1.698738	1.833340
O	-0.162628	-1.597439	2.984083

BP86/def2-SVP
CPCM(acetone,PDens=25)
29
Energy = -2082.5718841 Eh

Mn	0.059755	-1.836523	0.000000
N	1.352715	-0.194087	0.000000
C	2.703670	-0.278143	0.000000
C	3.533152	0.848300	0.000000
C	2.936197	2.121267	0.000000
C	1.541078	2.213017	0.000000
C	0.752282	1.039733	0.000000
C	-0.716957	1.010343	0.000000
C	-1.515186	2.181035	0.000000
C	-2.912854	2.080250	0.000000
C	-3.516254	0.807428	0.000000
C	-2.723343	-0.356693	0.000000
C	-1.314131	-0.285381	0.000000
H	3.134855	-1.289385	0.000000
H	4.625022	0.719472	-0.000001
H	3.553218	3.032819	0.000000
H	1.050938	3.196462	0.000000
H	-1.047331	3.178193	0.000000
H	-3.531639	2.991170	0.000000
H	-4.615272	0.719421	0.000000
H	-3.230913	-1.335074	0.000000
C	1.386798	-3.084479	0.000000
O	2.206180	-3.916331	0.000000
C	-1.218526	-3.093575	0.000000
O	-2.061206	-3.902172	0.000000
C	-0.054554	-1.694625	-1.831954
O	-0.161571	-1.588908	-2.982685
C	-0.054554	-1.694626	1.831954
O	-0.161571	-1.588909	2.982685

BP86/def2-SVP
CPCM(methanol,PDens=25)
29
Energy = -2082.5720518 Eh

Mn	0.059794	-1.836567	0.000000
N	1.352752	-0.194084	0.000000
C	2.703715	-0.278179	0.000000
C	3.533158	0.848300	0.000000
C	2.936213	2.121254	0.000000
C	1.541066	2.213019	0.000000
C	0.752346	1.039731	0.000000
C	-0.716965	1.010310	0.000000
C	-1.515216	2.180984	0.000000
C	-2.912926	2.080184	0.000000
C	-3.516364	0.807377	0.000000
C	-2.723396	-0.356751	0.000000
C	-1.314177	-0.285393	0.000000
H	3.134980	-1.289371	0.000000
H	4.625023	0.719489	-0.000001
H	3.553224	3.032799	0.000000
H	1.051004	3.196498	0.000000
H	-1.047374	3.178137	0.000000
H	-3.531680	2.991127	0.000000
H	-4.615384	0.719369	0.000000
H	-3.230958	-1.335145	0.000000
C	1.386719	-3.084640	0.000000
O	2.205701	-3.916898	0.000000
C	-1.218367	-3.093588	0.000000
O	-2.061028	-3.902291	0.000000
C	-0.054423	-1.694397	-1.831881
O	-0.161507	-1.588438	-2.982609
C	-0.054423	-1.694397	1.831882
O	-0.161507	-1.588438	2.982609

BP86/def2-SVP
CPCM(acetonitrile,PDens=25)
29
Energy = -2082.5720763 Eh

Mn	0.059800	-1.836573	0.000000
N	1.352758	-0.194083	0.000000
C	2.703722	-0.278185	0.000000
C	3.533159	0.848300	0.000000
C	2.936216	2.121252	0.000000
C	1.541064	2.213019	0.000000
C	0.752356	1.039731	0.000000
C	-0.716966	1.010305	0.000000
C	-1.515221	2.180976	0.000000
C	-2.912937	2.080174	0.000000
C	-3.516380	0.807369	0.000000
C	-2.723404	-0.356759	0.000000
C	-1.314184	-0.285395	0.000000
H	3.134998	-1.289369	0.000000
H	4.625023	0.719491	-0.000001
H	3.553225	3.032796	0.000000
H	1.051013	3.196504	0.000000
H	-1.047380	3.178129	0.000000
H	-3.531686	2.991121	0.000000
H	-4.615401	0.719362	0.000000
H	-3.230965	-1.335156	0.000000
C	1.386708	-3.084664	0.000000
O	2.205631	-3.916981	0.000000
C	-1.218344	-3.093589	0.000000
O	-2.061002	-3.902308	0.000000
C	-0.054404	-1.694364	-1.831871
O	-0.161497	-1.588369	-2.982598
C	-0.054404	-1.694364	1.831871
O	-0.161497	-1.588369	2.982598

BP86/def2-SVP
CPCM(water,PDens=25)
29
Energy = -2082.5722186 Eh

Mn	0.059834	-1.836611	0.000000
N	1.352790	-0.194081	0.000000
C	2.703760	-0.278215	0.000000
C	3.533164	0.848300	0.000000
C	2.936229	2.121241	0.000000
C	1.541053	2.213020	0.000000
C	0.752411	1.039728	0.000000
C	-0.716972	1.010276	0.000000
C	-1.515247	2.180932	0.000000
C	-2.912999	2.080118	0.000000
C	-3.516474	0.807325	0.000000
C	-2.723449	-0.356808	0.000000
C	-1.314224	-0.285405	0.000000
H	3.135104	-1.289358	0.000000
H	4.625023	0.719506	-0.000001
H	3.553229	3.032779	0.000000
H	1.051068	3.196534	0.000000
H	-1.047416	3.178080	0.000000
H	-3.531722	2.991085	0.000000
H	-4.615498	0.719318	0.000000
H	-3.231004	-1.335217	0.000000
C	1.386642	-3.084802	0.000000
O	2.205223	-3.917464	0.000000
C	-1.218209	-3.093599	0.000000
O	-2.060854	-3.902407	0.000000
C	-0.054291	-1.694169	-1.831809
O	-0.161441	-1.587967	-2.982534
C	-0.054291	-1.694169	1.831809
O	-0.161441	-1.587968	2.982534

DFT-optimized structure of [$Mn_2(CO)_{10}$] on BP86/def2-SVP level of theory.

22
Energy= -3434.8366863 Eh

Mn	-3.183769	-0.047707	-0.341211
Mn	-0.317568	0.366639	0.248733
C	-4.928897	-0.300009	-0.700386
O	-6.055473	-0.462897	-0.932254
C	1.427545	0.618957	0.607965
O	2.554111	0.781854	0.839874
C	-2.707009	-1.764754	-0.833726
O	-2.462360	-2.851067	-1.154347
C	-3.337839	-0.603225	1.415431
O	-3.489390	-0.960065	2.507413
C	-3.421326	1.703932	0.200528
O	-3.625322	2.796124	0.529506
C	-2.790502	0.542381	-2.048631
O	-2.598310	0.905081	-3.132253
C	-0.378286	-1.281734	1.083790
O	-0.361625	-2.309068	1.619545
C	-0.883339	1.171295	1.814161
O	-1.183879	1.684601	2.808605
C	-0.495992	1.980422	-0.635569
O	-0.553249	3.001874	-1.179705
C	0.008948	-0.472615	-1.365943
O	0.268812	-0.991811	-2.368756

7. References

(1) Ward, J. S.; Lynam, J. M.; Moir, J. W. B.; Sanin, D. E.; Mountford, A. P.; Fairlamb, I. J. S. A Therapeutically Viable Photo-Activated Manganese-Based CO-Releasing Molecule (Photo-CO-RM). *Dalton Trans.* **2012**, *41* (35), 10514–10517. https://doi.org/10.1039/C2DT31588B.

(2) DeFlores, L. P.; Nicodemus, R. A.; Tokmakoff, A. Two-Dimensional Fourier Transform Spectroscopy in the Pump-Probe Geometry. *Opt. Lett.* **2007**, *32* (20), 2966–2968. https://doi.org/10.1364/OL.32.002966.

(3) Shim, S.-H.; Zanni, M. T. How to Turn Your Pump–Probe Instrument into a Multidimensional Spectrometer: 2D IR and Vis Spectroscopiesvia Pulse Shaping. *Phys. Chem. Chem. Phys.* **2009**, *11* (5), 748–761. https://doi.org/10.1039/B813817F.

(4) Hamm, P.; Zanni, M. *Concepts and Methods of 2D Infrared Spectroscopy*; Cambridge University Press: Cambridge, 2011. https://doi.org/10.1017/CBO9780511675935.
